# Pathogenic variants damage cell composition and single cell transcription in cardiomyopathies

**DOI:** 10.1126/science.abo1984

**Published:** 2022-08-05

**Authors:** Daniel Reichart, Eric L. Lindberg, Henrike Maatz, Antonio M.A. Miranda, Anissa Viveiros, Nikolay Shvetsov, Anna Gärtner, Emily R. Nadelmann, Michael Lee, Kazumasa Kanemaru, Jorge Ruiz-Orera, Viktoria Strohmenger, Daniel M. DeLaughter, Giannino Patone, Hao Zhang, Andrew Woehler, Christoph Lippert, Yuri Kim, Eleonora Adami, Joshua M. Gorham, Sam N. Barnett, Kemar Brown, Rachel J. Buchan, Rasheda A. Chowdhury, Chrystalla Constantinou, James Cranley, Leanne E. Felkin, Henrik Fox, Ahla Ghauri, Jan Gummert, Masatoshi Kanda, Ruoyan Li, Lukas Mach, Barbara McDonough, Sara Samari, Farnoush Shahriaran, Clarence Yapp, Caroline Stanasiuk, Pantazis I. Theotokis, Fabian J. Theis, Antoon van den Bogaerdt, Hiroko Wakimoto, James S. Ware, Catherine L. Worth, Paul J.R. Barton, Young-Ae Lee, Sarah A. Teichmann, Hendrik Milting, Michela Noseda, Gavin Y. Oudit, Matthias Heinig, Jonathan G. Seidman, Norbert Hubner, Christine E. Seidman

**Affiliations:** 1. Department of Genetics, Harvard Medical School, Boston MA, 02115 USA; 2. Cardiovascular Division, Brigham and Women’s Hospital Boston MA, 02115 USA; 3. Department of Medicine I, University Hospital, LMU Munich, 80336 Munich, Germany; 4. Cardiovascular and Metabolic Sciences, Max Delbrück Center for Molecular Medicine in the Helmholtz Association (MDC), 13125 Berlin, Germany; 5. DZHK (German Centre for Cardiovascular Research), Partner Site Berlin, 10785 Berlin, Germany; 6. National Heart and Lung Institute, Imperial College London, London SW3 6LY, UK; 7. British Heart Foundation Centre for Research Excellence and Centre for Regenerative Medicine, Imperial College London WC2R 2LS, UK; 8. Division of Cardiology, Department of Medicine, Faculty of Medicine and Dentistry, University of Alberta, Edmonton, Alberta T6G 2R3, Canada; 9. Mazankowski Alberta Heart Institute, Faculty of Medicine and Dentistry, University of Alberta, Edmonton, Alberta T6G 2R3, Canada; 10. Erich and Hanna Klessmann Institute, Heart and Diabetes Center NRW, University Hospital of the Ruhr-University Bochum, 32545 Bad Oeynhausen, Germany; 11. Cellular Genetics Programme, Wellcome Sanger Institute, Wellcome Genome Campus, Hinxton CB10 1SA, UK; 12. Walter-Brendel-Centre of Experimental Medicine, Ludwig-Maximilian University of Munich, 81377 Munich, Germany; 13. Howard Hughes Medical Institute, Bethesda MD, 20815-6789, USA; 14. Systems Biology Imaging Platform, Berlin Institute for Medical Systems Biology (BIMSB), Max-Delbrück-Center for Molecular Medicine in the Helmholtz Association (MDC), 10115 Berlin, Germany; 15. Digital Health-Machine Learning group, Hasso Plattner Institute for Digital Engineering, University of Potsdam, 14482 Potsdam, Germany; 16. Hasso Plattner Institute for Digital Health, Icahn School of Medicine at Mount Sinai, NY 10029, USA; 17. Cardiac Unit, Massachusetts General Hospital, Boston, MA 02114, USA; 18. Royal Brompton and Harefield Hospitals, Guy's and St. Thomas' NHS Foundation Trust, London SW3 6NR, UK; 19. Heart and Diabetes Center NRW, Clinic for Thoracic and Cardiovascular Surgery, University Hospital of the Ruhr-University, 32545 Bad Oeynhausen, Germany; 20. Max Delbrück Center for Molecular Medicine in the Helmholtz Association (MDC), 13125 Berlin, Germany; 21. Department of Rheumatology and Clinical Immunology, Sapporo Medical University School of Medicine, Sapporo 060-8556, Japan; 22. Computational Health Center, Helmholtz Zentrum München Deutsches Forschungszentrum für Gesundheit und Umwelt (GmbH), 85764 Neuherberg, Germany; 23. Laboratory of Systems Pharmacology, Harvard Medical School, Boston, MA 02115, USA; 24. MRC London Institute of Medical Sciences, Imperial College London, London W12 0NN, UK; 25. ETB-Bislife Foundation, POB 309, 2300 AH Leiden, The Netherlands.; 26. Clinic for Pediatric Allergy, Experimental and Clinical Research Center, Charité-Universitätsmedizin Berlin, 13125 Berlin, Germany; 27. Department of Physics, Cavendish Laboratory, University of Cambridge, Cambridge CB3 0HE, UK; 28. Department of Informatics, Technische Universitaet Muenchen (TUM), 85748 Munich, Germany; 29. DZHK (German Centre for Cardiovascular Research), Munich Heart Association, Partner Site Munich, 10785 Berlin, Germany; 30. Charité-Universitätsmedizin Berlin, 10117 Berlin, Germany

## Abstract

Pathogenic variants in genes that cause dilated (DCM) and arrhythmogenic cardiomyopathies (ACM) convey high risks for the development of heart failure through unknown mechanisms. Using single nucleus RNA sequencing (snRNAseq), we characterized the transcriptome of 880,000 nuclei from 18 control and 61 failing, non-ischemic human hearts with pathogenic variants in DCM and ACM genes or idiopathic disease. We performed genotype-stratified analyses of the ventricular cell lineages and transcriptional states. The resultant DCM and ACM ventricular cell atlas demonstrated distinct right and left ventricular responses, highlighting genotype-associated pathways, intercellular interactions, and differential gene expression at single cell resolution. Together these data illuminate both shared and distinct cellular and molecular architectures of human heart failure and suggest candidate therapeutic targets.

## One-Sentence Summary

Single nucleus analyses define ventricular responses in human cardiomyopathies with pathogenic variants.

## Introduction

Dilated cardiomyopathy (DCM), a prevalent disorder occurring in 1:250 individuals, is characterized by left ventricular (LV) dilatation, cardiomyocyte loss with fibrotic replacement, and impaired contractility (1). Arrhythmogenic cardiomyopathy (ACM) similarly incites ventricular dysfunction, often with more prominent right ventricular (RV) involvement, high arrhythmogenic burden, and fibrofatty accumulations (2). Both disorders can arise from genetic causes (1, 2). DCM genes encode proteins involved in contractility (titin; *TTN*, troponin T; *TNNT2*, troponin C; *TNNC1*, tropomyosin; *TPM1*, and filamin C; *FLNC*), that regulate cardiac splicing (RNA-binding motif protein; *RBM20*) or calcium sequestration (phospholamban; *PLN)*, and maintain cytoskeletal (desmin; *DES*) or nuclear (lamin A/C; *LMNA)* integrity. ACM genes often encode desmosome proteins including plakophilin-2 (*PKP2*) and desmoplakin (*DSP*). The cardiomyocyte-specific expression and damaging effects of pathogenic variants (PVs) in many DCM and ACM genes propel the development of arrhythmias and heart failure (HF), a highly morbid condition affecting 23 million individuals worldwide (3).

We hypothesized that PVs in different genes would evoke distinct single-cell molecular phenotypes. To address this, we studied the molecular signals underlying HF pathogenesis using snRNAseq of human hearts with advanced DCM and ACM compared to non-failing donor (control) hearts. We revealed differences in the cellular landscape and transcriptional changes between several DCM and ACM genotypes. By leveraging machine learning approaches we illuminated genotype-specific molecular responses, as validated by reconstructing the underlying PVs using snRNAseq data.

## Results

### Study Cohort

**Figure 1:**
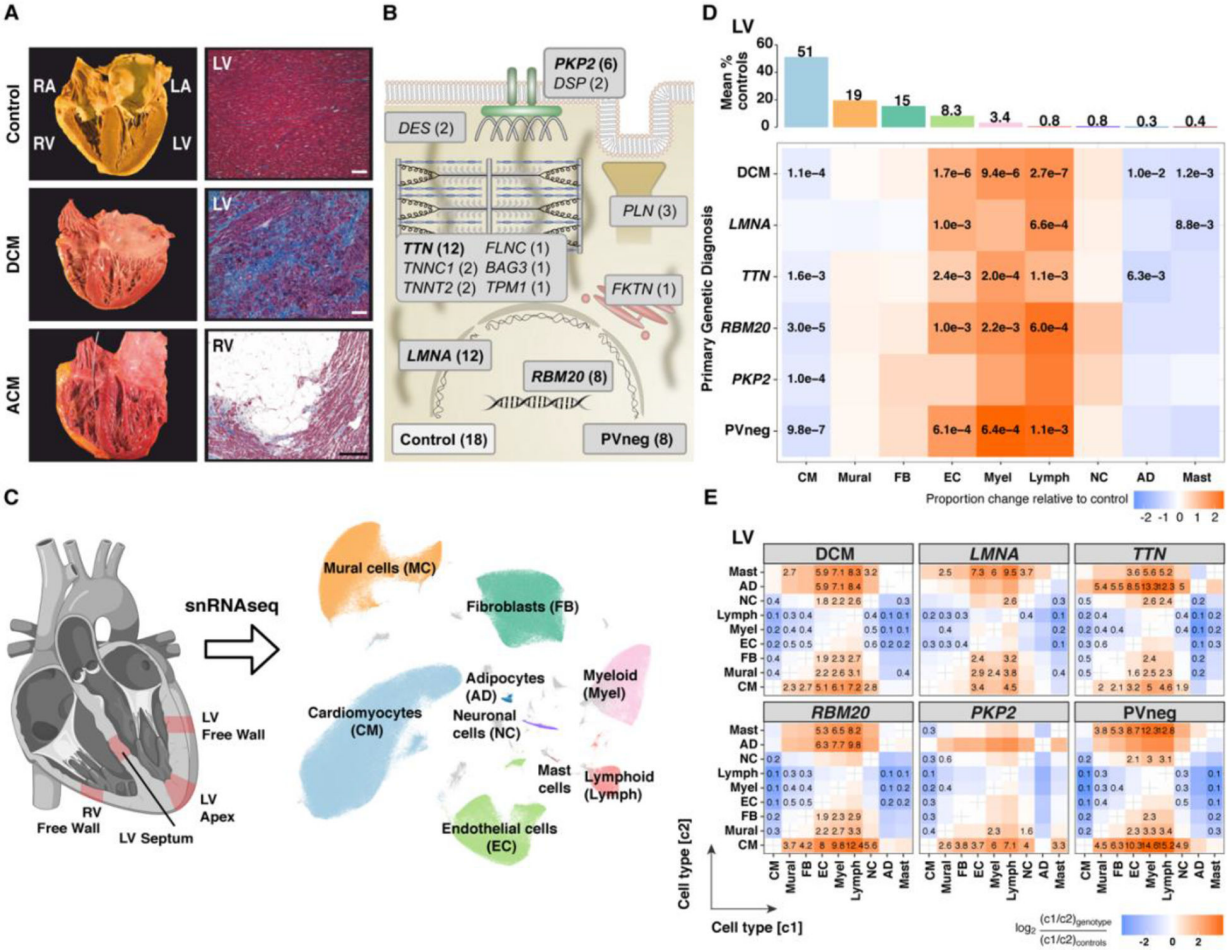
PVs and unexplained causes of DCM and ACM alter cardiac morphology, histopathology, and cellular compositions. (**A**) Comparisons of normal cardiac anatomy and histology to DCM, demonstrating LV dilation with fibrosis, and to ACM, showing RV dilation with fibrofatty degeneration (Masson trichrome staining, magnification 100x, bar 10μm). (**B**) Schematic depiction of the functions of DCM and ACM genes with PVs (number indicates unique genotypes, bolded denotes ≥6 patients) in studied tissues. (**C**) Single nuclei isolated from transmural LV (free wall, apex or septum) and RV sections were processed using 10X Chromium 3’ chemistry. UMAP embedding of 881,081 nuclei delineated ten cell types and unassigned populations (gray). (**D**) Upper panel: Mean abundance (%) of cell types in control LVs. Lower panel: Proportional changes of cell types in specified genotypes or aggregated across DCM genotypes. Proportional changes are scaled by color: increased (red) or decreased (blue) in disease versus control. *p*-values are indicated for significant proportional changes, FDR≤0.05. (**E**) Pairwise cell type abundance ratios in specified genotypes or aggregated DCM genotypes in LVs relative. Color scale, FDR, significance depicted as in (D).

We studied LV and RV tissues (Fig. 1A), obtained prior to any mechanical support in 61 patients and 18 controls (Tables S1, S2) including 12 controls previously reported (4). Thirty-eight independent PVs were identified in three DCM genes (*LMNA;* n=12, *RMB20*; n=8, *TTN;* n=12) and in the ACM gene *PKP2* (n=6), while no PVs were detected in eight DCM patients (PVneg) (Fig. 1B). Analyses were performed for these five genotypes individually (n=46), for aggregated DCM genotypes (*LMNA, RBM20, TTN*, PVneg), ACM (*PKP2*), and controls. Additionally, we generated data from 15 PVs across *PLN, BAG3, DES, FLNC, FKTN, TNNC1, TNNT2, TMP1*, and *DSP* (Table S1, S2). Due to few recurrent PVs, these genes were excluded in downstream analyses, except where indicated.

Males predominated among patients (60%) and controls (72%) (Fig. S1A). The mean age of patients was 48.4±4.3 years exclusive of *RBM20* (mean age 32.9±14.6 years). Clinical manifestations indicated similar LV dysfunction in *LMNA, TTN*, and PVneg patients, but greater LV dilatation and reduced systolic contraction in *RBM20*, and preserved LV with reduced RV function in *PKP2*. *LMNA* and *TTN* patients received more pacemaker/resynchronization therapies than those with other genotypes (Table S1).

### Disease-associated compositional changes of cell types

Nuclei were isolated as described (4) from full thickness LV free wall (FW), apex (AP), septum (S) and RV free wall (RV) (Fig. 1C). We compared ~500,000 high-quality nuclei from patients’ ventricular tissues and ~380,000 nuclei from controls (Fig. S1B-E). After pre-processing and quality control filtering, data were integrated across samples using Harmony prior to constructing manifolds using Uniform Manifold Approximation and Projections (UMAPs). Clustering identified ten major cardiac cell types encompassing ventricular cardiomyocytes (CM), fibroblasts (FB), adipocytes (AD), pericytes and smooth muscle cells (mural, MC), endothelial (EC), myeloid and lymphoid (immune), neuronal (NC) and mast cells (Figs. 1C) with 71 distinct cell states. States of the same cell type shared a transcriptional profile but also expressed distinct genes, which implied biological differences.

Cell type abundances in sample replicates were highly correlated (Pearson coefficient 0.74 - 0.99) (Figs. S2A, B). Cell composition, states, and transcript counts across the FW, AP, and S showed high similarities, and therefore are reported grouped together as LV (Figs. S2C, D).

Using center log ratio- (CLR) transformed abundance of cell types, we considered the effects of sex on LV and RV cell compositions in DCM (10 female, 29 male) and control (7 female, 11 male) hearts (Fig. S2E). Myeloid cells showed a modest sex-specific difference (FDR=0.016). Only *LMNA* tissues (from 7 males, 5 females) were sufficient for genotype-specific, sex-associated analyses. Female LV myeloid cells were increased (FDR 0.05), paralleling the findings in DCM vs controls. In addition, RV endothelial cells showed a modest female-specific increase between *LMNA* patients and controls (FDR 0.048).

The proportions of LV nuclei across the genotypes demonstrated depletion of CMs except in *LMNA*, and increased ECs and immune cells except in *PKP2* (Figs. 1D, S3). In RVs (Fig. S4), CMs were depleted in the DCM subgroup except for *TTN*, while ECs were increased in *LMNA*, *TTN*, and *RBM20*, and immune cells were not changed. FBs were not increased in LVs and RVs (Figs. 1D), despite histopathological findings of fibrosis, which implied the acquisition of a secretory rather than a proliferative phenotype.

Individual-level abundances for cell types (LV, Table S3; RV, Table S4) and cell states (Table S5) are provided for controls and patients. Different cell type abundances in disease compared to control LVs remained significant using a linear model adjusting for age. Pairwise cell type ratios in disease LVs compared to controls confirmed loss of CMs and showed accompanying increased ECs, lymphoid and myeloid cells, and altered ratios (FB and mural compared to CM) with quantitative, genotype-specific differences (Figs. 1E). *RBM20* and PVneg, respectively showed greatest shifts in EC:CM (8- and 10.3-fold), myeloid:CM (9.8- and 14.6-fold), and lymphoid:CM (12.4- and 15.2-fold). By contrast, pairwise ratios of all cell types compared to CM were modest or unchanged in *LMNA*.

### Genotypes diversify cell types and states

#### Cardiomyocytes

**Figure 2:**
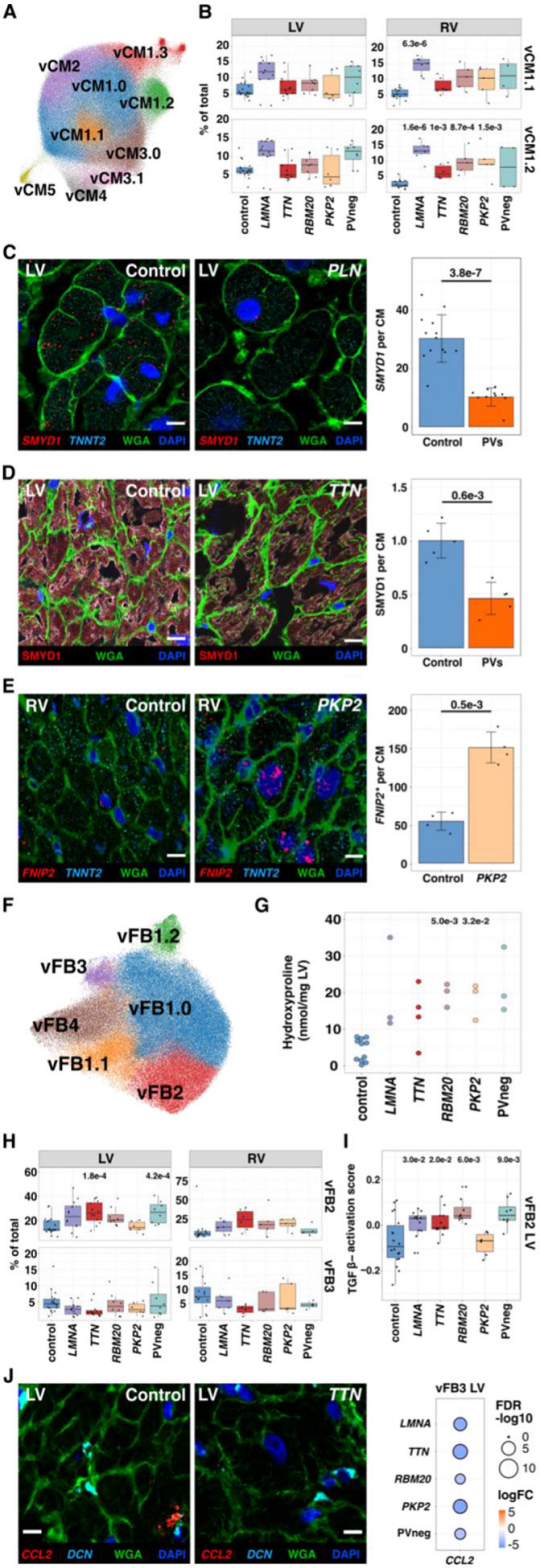
Cardiomyocytes and fibroblast states in control, DCM, and ACM ventricles. (**A**) UMAP depicting CM states in all tissues. (**B**) Control and disease LVs and RVs abundance analyses for vCM1.1 (upper panel) and vCM1.2 (lower panel). (**C**) Single-molecule RNA fluorescent *in situ* hybridization exemplifies decreased *SMYD1* (red) expression in CMs (identified by *TNNT2* transcripts, cyan) within a DCM heart with a PV in *PLN* (phospholamban). Cell boundaries, WGA-stained (green); nuclei DAPI-stained (blue); bar 10μm. Quantified expression (spots per CM) and *p*-values from four independent control and disease LVs with PVs were assessed. (**D**) Immunohistochemistry validated decreased SMYD1 (red) protein in CMs (identified by troponin T staining, Fig. S6E) in TTN LV section. Cell boundaries, WGA-stained (green); nuclei DAPI-stained (blue); bar 10μm. Quantified protein levels (intensity per CM) and *p*-values were assessed from five independent control and DCM LVs with PVs. (**E**) Single-molecule RNA fluorescent *in situ* hybridization demonstrated increased expression of *FNIP2* (red). CMs, nuclei, and cell boundaries are labeled as in C; bar 10μm. Quantified expression of *FNIP2* (spots per CM and H-score; Methods) and *p*-values reflect analyses of two independent control and *PKP2* samples. (**F**) UMAP depicting FB states. (**G**) Hydroxyproline assay (HPA) quantifies cardiac collagen content for each genotype. (**H**) Control and disease LVs and RVs abundance analyses for vFB2 (upper panel) and vFB3 (lower panel). (**I**) Pathway score of TGFβ activation in LV vFB2. (**J**) Single-molecule RNA fluorescent *in situ* hybridization shows decreased expression of *CCL2* (red) in vFB3 (DCN, cyan) in disease compared to controls. WGA-stained (green); Nuclei DAPI-stained (blue); bar 10μm. Dot plot illustrating fold-change (log2FC) and significance (−log10(FDR)) of *CCL2* expression in LV vFB3 across genotypes.

Disease and control ventricles exhibited the previously described (vCM1.0-5; (4)) and four new cardiomyocyte states: vCM1.1, 1.2, 1.3, 3.1 (Fig. 2A-B, Table S6). Across all vCM cell states (Fig. S5), differentially expressed genes (DEGs) in disease connoted increased stress (for example, *NPPB)* and apoptosis (Tables S7-12, Fig. S6A). While these findings implied late-stage transcriptional convergence, 20-40% of DEGs were genotype-specific (Fig. S5). Only PVneg reduced *MYH6* expression, increasing *MYH7:MYH6* ratios (Fig. S6B). Conversely, only vCMs with PVs downregulated *SMYD1* (Figs. 2C, S6C-E, Table S13), a cardioprotective muscle-specific histone methyltransferase that regulates sarcomere assembly and mitochondrial energetics (5, 6), and *ADRB1* (β1-adrenergic receptor; Fig. S6C) indicative of adrenergic activation, which is therapeutically targeted by β-blockade medicines (7, 8). Genotype-selective responses included upregulation of *FNIP2* (Fig. 2D), inhibiting AMP-activated protein kinase activity and oxidative metabolism (9-11) in *LMNA* and *PKP2*, and *CPEB4*, an RNA-binding protein that activates glycolysis and stimulates fibrosis (12) in *LMNA, RBM20*, and *PKP2* (Fig. S6F).

Proportional differences in vCM states (Figs. S6G, S7, S8) varied among genotypes and controls and were more prominent in RVs than LVs, perhaps reflecting hemodynamic differences. PVs generally increased vCM1.1 and 1.2, with accompanying decreased vCM3.1 in *LMNA* and *TTN* (Figs. 2B, S7A, S8A-D). State-enriched DEGs for vCM1.1 included *MYO18B*, required for sarcomere formation (13), *XPR1*, regulating phosphate homeostasis (14), and *IGF1R* and *ROR1*, involved in cell survival (8). vCM1.2 had heightened expression of electrophysiologic genes including *PCDH7* that enables intercellular contacts (15, 16), but when overexpressed, impedes synaptic currents (17), *CAMK2D* modulating excitation-contraction coupling (18), and *ANK2*, harboring arrhythmogenic PVs (19, 20). vCM1.3 was modestly increased in *RBM20*, associated with the largest numbers of DEGs in all genotypes, and enriched for *BMP* receptors (*BMPR1A, BMPR1B* (21, 22); Figs. S7B, D) and *GPC5* that regulates BMP signaling (23). vCM3.1, reduced in *LMNA* and *TTN*, expressed early-adaptive transcriptional regulators of stress responses (*ATF3, ATF4)* and cardioprotection *(NR4A3;* Fig. S7E) (24). Only PVneg depleted vCM2, enriched for *SH3RF2*, an anti-apoptosis regulator of the *JNK* pathway (25). As DCM and ACM genes are highly and often selectively expressed in CMs, these disease-associated vCM states and DEGs defined intrinsic responses to PVs as well as cell-cell responses to CM death, depressed contractile performance, and arrhythmogenicity.

#### Fibroblasts

We identified four previously characterized (4) and two new ventricular FB states (vFB1.1, vFB1.2; Fig. 2E, Table S14). Across all vFB states, upregulated DEGs included genes involved in extracellular matrix (ECM) remodeling (Figs. S9-11, Table S15-21). *LMNA*, *TTN*, and *PKP2* increased fibrogenic signaling receptor, *EGFR* and *PKP2* also increased *AGTR1* (Fig. S10A) that enables EGFR-transactivation (26, 27). The expression of profibrotic *TGFβ2* was universally increased (Fig. S10B). DCM hearts were enriched for *PCOLCE2*, promoting insoluble collagen formation (28), and *LMNA, TTN*, and PVneg downregulated metalloproteinase inhibitors *TIMP1 and 3* (Fig. S10C). Notably, collagen genes showed genotype-specific expression. *COL4A1* and *COL4A2* were upregulated in *LMNA, TTN* and *PKP2* while *COL4A5* and *COL24A1* were enriched in PVneg. Thus, although ECM upregulation and collagen deposition (Figs. 2F, S10D-F) was a shared feature, genotype and chamber influenced composition and organization.

FB state abundance was also altered across genotypes (Figs. S11A, S12A-D). vFB1.1 and vFB1.2 expressed canonical vFB1.0 genes but vFB1.1 also up-regulated *APOD, APOE* and *APOO*, typifying lipogenic fibroblasts (29) and *CST3* (Fig. S13A), involved in matrix remodeling. vFB1.2-enriched genes are related to actin filament assembly (*DAAM1*) and chondrocyte differentiation (*GPC6*) (Fig. S13B). vFB2 expressed prominent profibrotic genes including *TGFβ*-targets (increased in DCM LVs) and fibrogenic *IL11* (30) (highest in *RBM20* LVs) (Figs. 2H, S14A-C). vFB2 was increased in *TTN* and PVneg and modestly in other DCM LVs (Figs. 2G-H, S11). vFB3, diminished in hearts with PVs, expressed proinflammatory cytokine genes (*CCL2*) and genes related to *OSM* signaling *(IL6ST, OSMR*, and oncostatin M-target genes) (Figs. 2I, S14D). The resultant increased ratio of vFB2:vFB3 and altered DEGs suggested dysregulation of fibroblast-to-macrophage interactions that would promote deleterious ECM remodeling, particularly in *TTN* LV and RV (Figs. S12C, D).

### Smooth muscle cells and pericytes

**Figure 3:**
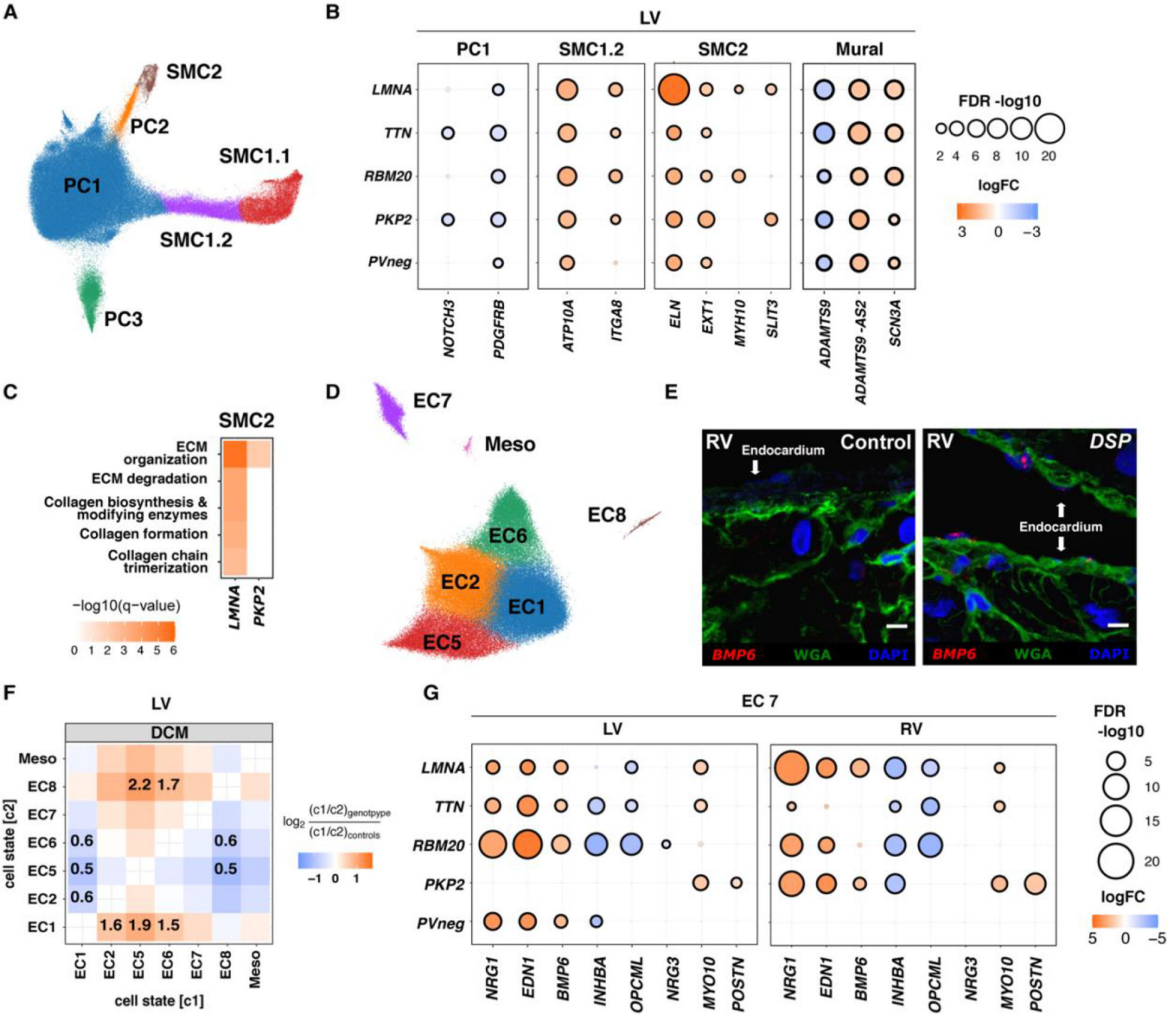
Mural and endothelial cell states in control, DCM, and ACM hearts. (**A**) UMAP depicting pericyte (PC) and smooth muscle cell (SMC) states in all tissues. (**B**) Dot plots illustrate levels (fold-change; logFC) and significance (−log10(FDR)) of selected DEGs in LV PC1, SMC1.2, SMC2, and MC (PC and SMC) across genotypes. (**C**) KEGG pathway analysis of DEGs in SMC2 among genotypes with ≥1 enriched pathways. Color intensity denotes enrichment significance (−log10(FDR)). (**D**) UMAP depicting EC states in all tissues. (**E**) Single-molecule RNA fluorescent *in situ* hybridization exemplifies *BMP6* (red) expression in disease endocardium from a *DSP* (desmoplakin) compared to control RVs. Cell boundaries, WGA-stained (green); Nuclei DAPI-stained (blue); Bar 10μm. (**F**) Pairwise cell state abundance ratios in DCM LVs relative to controls. Proportional changes are scaled by color: increased (red) or decreased (blue) in disease versus control. *p*-values are indicated for significant proportional changes, FDR<0.05. (**G**) Dot plots illustrate LV and RV levels (fold-change; logFC) and significance (−log10(FDR)) of selected DEGs in EC7 across genotypes. Dot size and color are defined in (B).

Three previously described pericyte (PC1-3) and three smooth muscle cell states (SMC1.1, SMC1.2, SMC2, Fig. 3A, Table S22) were identified. DEGs across all states (Fig. S15, Tables S23-28) showed upregulation of the sodium channel *SCN3A*, with unknown vascular functions, and the noncoding antisense *ADAMTS9-AS2*, with concordant downregulation of *ADAMTS9*, a metalloprotease involved in ECM remodeling (31, 32) (Figs. 3B, S16A). Overall DEGs implied that disease evoked signals to synthesize specific ECM and integrin components.

DEGs in PC states included downregulation of two central signaling receptors, *NOTCH3* and *PDGFRB*, in *PKP2* and *TTN* PC1 (Fig. 3B). *NOTCH3* is required for SMC maturation and deficiency causes pericyte dysfunction and arteriovenous malformations. Notch signaling regulates *PDGFRB*, necessary for angiogenesis and PC recruitment (33-37).

DEGs in SMCs subdivided the previously identified canonical SMC1 (4) into two states. SMC1.1, had higher expression of *ACTA2, MYH11*, and *TAGLN*. SMC1.2, strongly expressed *ITGA8*, required for maintenance of SMC contractile phenotype (38, 39), and *ATP10A*, suggesting vascular stiffness and increasing diastolic dysfunction (Fig. 3B) Methylation of the *ATP10A* locus in SMC decreases with age and atherosclerosis (40). SMC1.2 was enriched in *LMNA, TTN* and PVneg RVs (Figs. S17, S18). SMC2 expressed high levels of genes involved in collagen and elastic fiber formation (*ELN, LAMA2*) and *MYH10*, demarcating dedifferentiated SMCs with secretory properties (Figs. S16B, S17C). *LMNA* and *PKP2* SMC2 upregulated *SLIT3*, a stimulator of fibroblast activity (41), and ECM synthesis and collagen formation genes (Figs. 3B-C). Collectively, disease remodeling of MC indicated modulation of PDGF and NOTCH signaling receptors and synthesis of selective ECM and integrin components.

### Endothelial cells

Characterization of ECs identified seven previously described cell states (4) (EC1, 2, 5-8 and Mesothelial) (Figs. 3D, S19-21, Table S29). Shared DEGs occurred across all and between genotypes (Table S30-35) with clear RV and LV differences (Fig. S19). Dysregulation of genes encoding factors involved in EC fate, blood vessel development and angiogenesis (*NOTCH4, FLT1, FGFR1, RGCC*) (42-44) in disease LVs indicated that vascular remodeling was a common HF feature.

EC composition in DCM RVs and LVs increased EC5 in comparison to controls (Fig. S21A), while genotype-specific cell state ratios in LVs generally decreased EC1 relative to EC2, EC5, and EC6 (Figs. 3F, S21C). Assessment of selective gene enrichment scores for proliferating cells, endothelial-to-mesenchymal transformation (EMT), and cell death showed no difference in disease compared to controls (Fig. S22; Table S36).

EC7 had the most DEGs and highest proportion of unique DEGs across all genotypes. Although initially defined as atrial-enriched (4) EC7 expressed endocardial-enriched genes (*SMOC1* (45), *NPR3* (46), and *POSTN* (47) (Figs. S20B-C), which prompted a revised annotation to endocardial cells. Interestingly, upregulated genes in EC7 from DCM LVs and *PKP2* RVs (Fig. 3G) encoded secreted proteins involved in myocardial stress-adaptation (*NRG1*) (48), CM force production (*EDN1*) (49, 50), and endocardial expansion during development or after cardiac injury (*BMP6)* (51, 52). Notably, *BMP6* was selectively upregulated in the endocardium of DCM LVs and ACM RVs (Fig. 3E). Conversely, *INHBA*, a TGFB superfamily member involved in atrioventricular canal development (53), and cell adhesion molecule *OPCML* were downregulated. In *PKP2*, an unconventional myosin promoting cell adhesion (*MYO10*), and *POSTN*, were upregulated in both ventricles. These data highlighted the involvement of the endocardium in chamber-specific remodeling of cardiomyopathies.

### Myeloid cells

**Figure 4:**
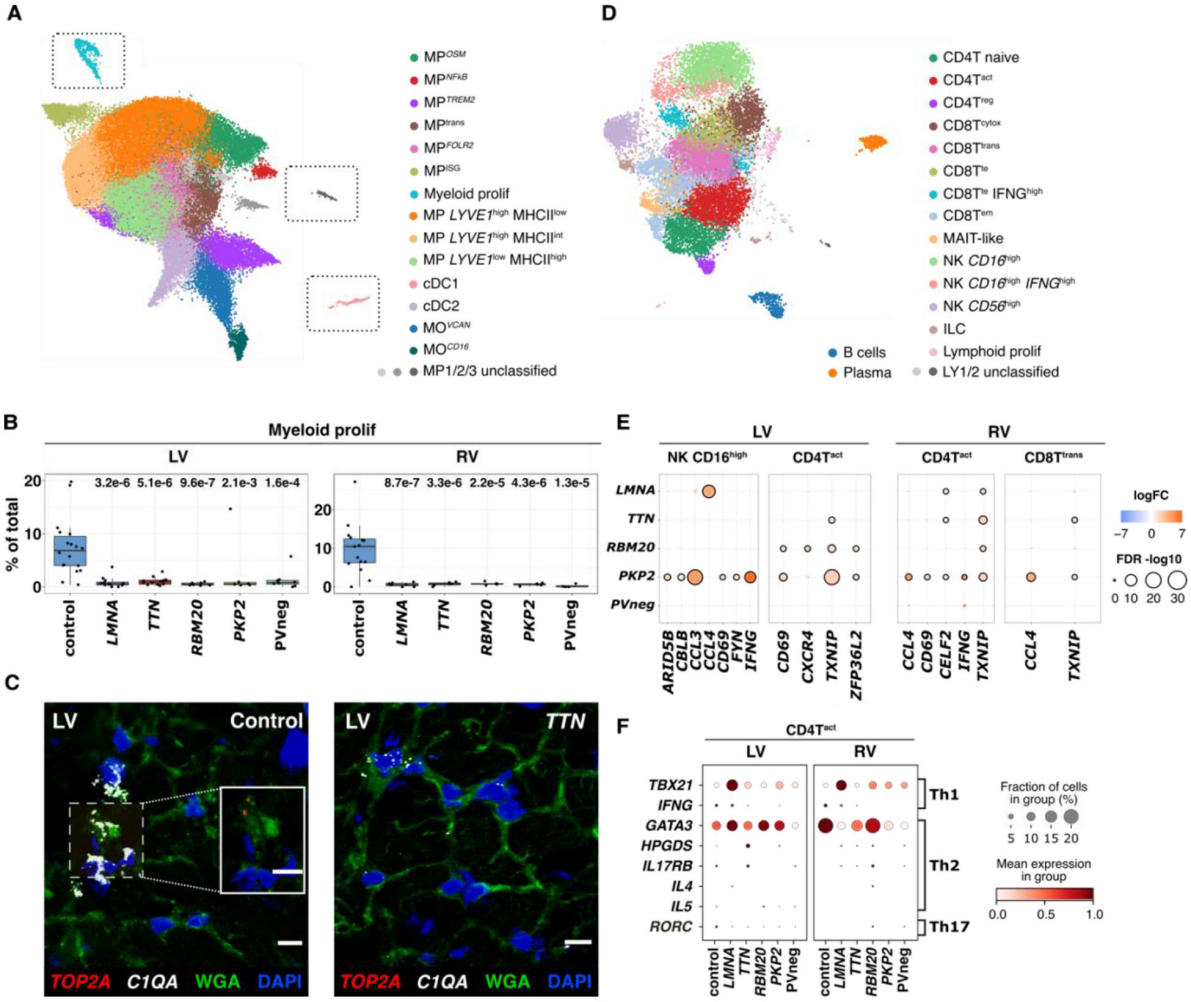
Immune cell states in control, DCM, and ACM hearts. (**A**) UMAP depicting myeloid states in all tissues. Unclassified MP1, 2, and 3 require future characterization. Gray boxes enclosing proliferating (prolif) MPs, unclassified MPs, and cDC1s indicate that these were manually juxtaposed toward other states for ease of representation. The unmodified UMAP is in Fig. S23. (**B**) Myeloid prolif have higher abundance (% total myeloids) in controls versus disease. *p*-values indicate significant differences in abundances. (**C**) Single-molecule RNA fluorescent *in situ* hybridization validated increased expression of *TOP2A* (red) and *C1QA* (white) in controls versus disease. Cell boundaries, WGA-stained (green); Nuclei DAPI-stained (blue); Bar 10μm. (**D**) UMAP depicting lymphoid cell states in all tissues. Unclassified LY1 and 2 require future characterization. (**E**) Dot plots show the level of fold-change (logFC) and significance (−log10(FDR)) of selected genes in LV NK CD16^hi^, LV and RV CD4T^act^, and RV CD8T^trans^ across genotypes. (**F**) Dot plots highlight gene expression levels of the Th1, 2 and 17 signatures in CD4T^act^ cells. Dot size, fraction (%) of expressing cells; color, mean expression level.

We classified 14 subclusters of myeloid cells comprising macrophages (MP), monocytes (MO), conventional dendritic (cDC1 and cDC2) and proliferating myeloids (Figs. 4A, S23-27, Tables S37-45). Distinct gene sets were unique to each genotype and were particularly pronounced in PVneg and *PKP2* in LVs (Fig. S24).

Although disease increased myeloid cells, the proportions of proliferating myeloids (Figs. 4B-C, S26-27) were consistently lower compared to controls, implying monocyte-infiltration. Tissue resident MPs *LYVE1*^high^MHCII^low^ and *LYVE1*^low^MHCII^high^ (Fig. S25C) were the most abundant myeloid cells (Figs. S27A-B). MP *LYVE1*^low^MHCII^high^ were increased in the RV of *LMNA* with similar trends in other genotypes (Fig. S27B). Proportions of MP^*OSM*^ were modestly decreased with downregulation of *OSM* in *TTN* ventricles (Fig. S28A), which would attenuate the MP^*OSM*^-vFB3 signaling axis (4), and mirrored the decreased OSM pathway activation score observed in vFB3 (Fig. S14B).

Prominent antigen presenting activities, were evident in MO^*VCAN*^, MO^*CD16*^, cDC1 and cDC2, also occurred in MP^*FOLR2*^, akin to tumor-associated MP (54). Across antigen-presenting MP, *RBM20* LVs showed the highest presentation of antigens based on MHCII genes (Figs. S28B-C) and more abundant cDC2 compared to the other genotypes (Fig. S27). *PKP2* LVs upregulated MP^ISG^ (Fig. S27) with interferon-stimulated genes (ISG) (55), perhaps contributing to inflammatory *PKP2* phenotypes (56).

### Lymphoids

We classified 15 lymphoid cell states, including T- and natural killer (NK)-cell subsets, innate lymphoid cells (ILC), B-cells, plasma cells plus proliferating lymphoids (Figs. 4D, S29-32, Table S46). Our experimental design did not enrich for immune cells, and few (<40) genotype specific DEGs were identified (Fig. S29). Proliferating lymphoids were rare and abundance was unchanged in disease (Figs. S30, 32A-D, Table S47-52).

The cardiac complexity of CD4+ and CD8+T-cells included naive (CD4T^naive^), activated (CD4T^act^), regulatory (CD4T^reg^) and cytotoxic (CD8T^cytox^), transitional (CD8T^trans^), terminal effector (CD8T^te^) and effector memory (CD8T^em^) cells (Fig. S31B) (57). We detected increased CD4T^act^ only within DCM samples (Figs. S32A-D). However, DEGs indicated lymphocyte activation (cytokines *IFNG, CCL3, CCL4* and signaling molecules *CBLB, FYN, TXNIP*), and maturation (cell surface receptors *CD69, CXCR4*), particularly in *PKP2* NK−, CD4+T, and CD8+T-cells (Fig. 4E).

CD4+helper T-cells are critical drivers in cardiomyopathy and myocarditis pathogenesis (58, 59). In *LMNA* CD4T^act^ we identified upregulation of *TBX21*, important for Th1 polarization. Conversely, Th2-polarization transcription factor *GATA3* was downregulated in PVneg (Fig. 4F).

### Neuronal cells

**Figure 5:**
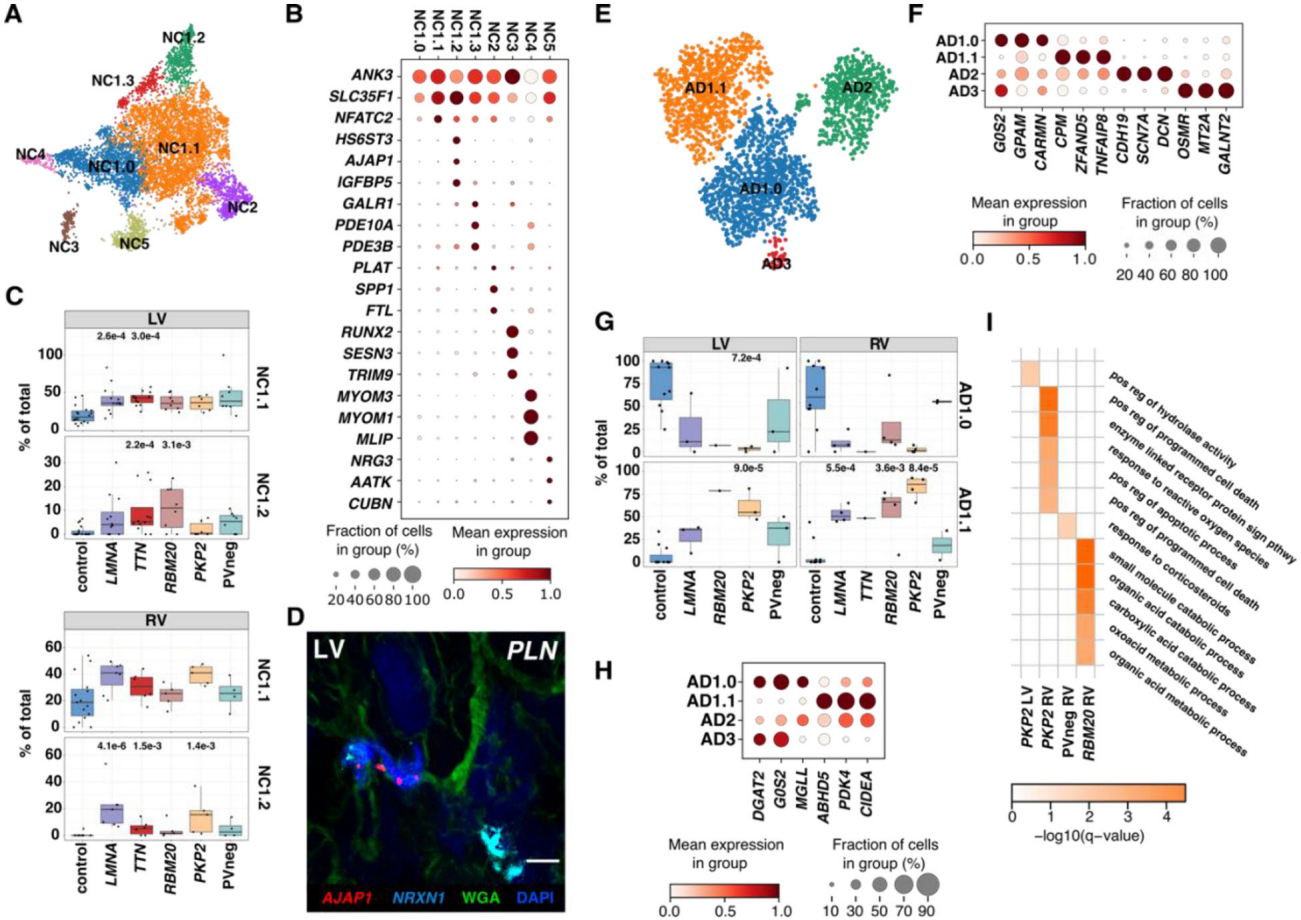
Neuronal and Adipocyte cell states in control, DCM, and ACM hearts. (**A**) UMAP depicting NC states in all tissues. (**B**) Dot plot highlights top marker genes for NC states. (**C**) LV and RV abundance analyses of NC1.1 and 1.2 in controls versus disease. *p*-values are indicated for significant proportional changes, FDR≤0.05. (**D**) Single-molecule RNA fluorescent *in situ* hybridization shows colocalization *AJAP1* (red), and *NRXN1* (cyan) in disease (exemplified in a DCM LV with a PV in *PLN* (phospholamban), demarcating the NC1.2 state. Cell boundaries, WGA-stained (green); Nuclei DAPI-stained (blue), Bar 10μm. (**E**) UMAP depicting AD states in all tissues. (**F**) Dot plot highlights top marker genes for AD states. (**G**) LVs and RVs abundance analyses demonstrate decreased AD1.0 and increased AD1.1 in disease versus control. (**H**) Dot plot of DEGs shows expression differences between AD states. (**I**) Heatmap of significantly enriched Gene Ontology Biological Processes terms based on significantly upregulated genes in diseased versus control ADs. Dot plots: Dot size, fraction (%) of expressing cells; color, mean expression level. *p*-values indicate significant differences; FDR≤0.05.

Analysis of cardiac NCs was limited by rarity of this cell type (Figs. 5A, S33, Table S53-57). Across all NC states, DEGs indicated increased *NFATC2* in LVs with PVs and *PKP2* RVs, and genotype-selective enrichment of *LRRK2*, an activator of a neurotoxic cascade (60). Other upregulated DEGs function in proteoglycan synthesis for neuronal myelination and axon regeneration (*XYLT1* and *HS3ST4*), and a complement inhibitor (*SUSD4)* that impacts neural function and morphology (61, 62). Genotype-specific DEGs were highest in *PKP2*.

We identified previously described (4) and three new NC states (Figs. 5B-C, S34, S35) that were genotype and chamber-selective: NC1.1 in *LMNA* and *TTN* LVs; NC1.2 in *TTN* and *RBM20* LVs and in *LMNA*, *TTN*, and *PKP2* RVs. NC1.1 was distinguished by the highest upregulation of *NFATC2*. NC1.2 upregulated genes associated with electrocardiogram intervals (*SLC35F1* and *AJAP1*; Fig. 5D), *IGFBP5*, involved in neuronal apoptosis and autophagy, and the ion channel and heart rate regulator *KCNK1* (63-65). NC1.3 was enriched for the neuromodulator receptor *GALR1*, and phosphodiesterases *PDE10A* and *PDE3B* that participate in neuroprotection and signaling (66-68). Dysregulated expression of genes involved in stress-responses and electrophysiology may account for characteristic life-threatening arrhythmias in DCM and PVs in *PKP2* (1, 2).

### Adipocytes

Similar to NCs, limited adipocytes were captured. We identified three adipocyte states previously found in controls: canonical AD1.0, expressing lipid metabolism genes; stromal AD2, expressing ECM genes; and immune AD3, expressing *OSMR* and cytokine responsive genes (Figs. 5E-F, S36, Table S60-64). While AD3 was not detected in disease, a fourth identified state, AD1.1 was almost exclusive to disease hearts (Figs. 5G, S37). Compositional analysis identified increased proportions of AD1.1 in *PKP2* LVs and RVs concurrent with decreased LV proportions of AD1.0. *LMNA* and *RBM20* RVs, unlike LVs, also increased proportions of AD1.1 (Figs. S38A-D).

DEGs between AD1.1 and AD1.0 revealed changes in fatty acid metabolism pathways (Fig. 5H). AD1.1 showed downregulation of *DGAT2*, encoding a triglyceride forming enzyme (69), and *G0S2* and *MGLL*, encoding lipolysis regulators (70). Conversely *ABHD5*, a positive lipolysis regulator (70, 71), *PDK4*, a kinase promoting the shift from glucose to fatty acid metabolism, and *CIDEA*, a regulator of adipose tissue energy expenditure, were upregulated. *PKP2* RVs which typically display fibrofatty replacement also showed an enrichment of Gene Ontology biological processes for apoptosis and cell death (Fig. 5I). These data implied genotype-specific state transitions or replacement of canonical adipocytes in DCM and ACM.

### Differential expression of GWAS genes in cardiomyopathies

Genome-wide association studies (GWAS) have identified common genetic variants associated with DCM. We selected candidate genes from 15 previously identified DCM loci (Table S67) and examined expression across cell types and genotypes (Fig. S39). Overall, GWAS genes were more often DEGs in LV and RV than expected by chance in snRNAseq data (LV: OR=7.0, P=0.0007; RV: OR=6.1, P=0.0009, one-sided Fisher’s exact test). Multiple genes showed cell type specific expressions, with the majority highly enriched in CM (*ALPK3, BAG3, FHOD3, FLNC, HSPB7, MLIP, SLC6A6, SMARCB1, SVIL* and *TTN*). Among these we observed both genotype- and chamber-specific expression differences. Heat shock protein *HSBP7* was reduced in TTN LVs and in *LMNA* and *PKP2* RVs. *SLC6A6*, a taurine and amino acid transporter with cardioprotective effects, was increased in both LV and RV across all genotypes except for *PKP2* and PVneg. *CDKN1A*, a cell cycle regulator and modulator of apoptosis (72) was increased in LMNA CMs from LVs but not RVs (72).

GWAS genes that were more broadly expressed across cell types included *MTSS1*, encoding a putative actin-cytoskeletal interactor. *MTSS1* showed highest and unchanged expression in myeloids and was widely increased in mural cells as well as in FBs in *LMNA*, *TTN*, and *PKP2* (Fig. S39), suggesting influences beyond direct effects on CM function (73, 74). *SLC39A8*, a lowly expressed cardiac solute carrier, was unchanged in CMs but increased in *LMNA* and *TTN* LV ECs. We suggest that cell-specific expression changes of GWAS genes may improve interpretation of their biologic effects.

### Predicted and altered cell-cell interactions across genotypes

By examining the expression of genes encoding for receptors and ligands, we inferred intercellular signaling and communication (75). We initially quantified the probability of cell-cell interactions and compared signaling between cell states, and then aggregated information to produce cell-specific and across-all-cell-types data, for each genotype relative to controls. This sequential approach accounted for differential abundances of cell states.

**Figure 6:**
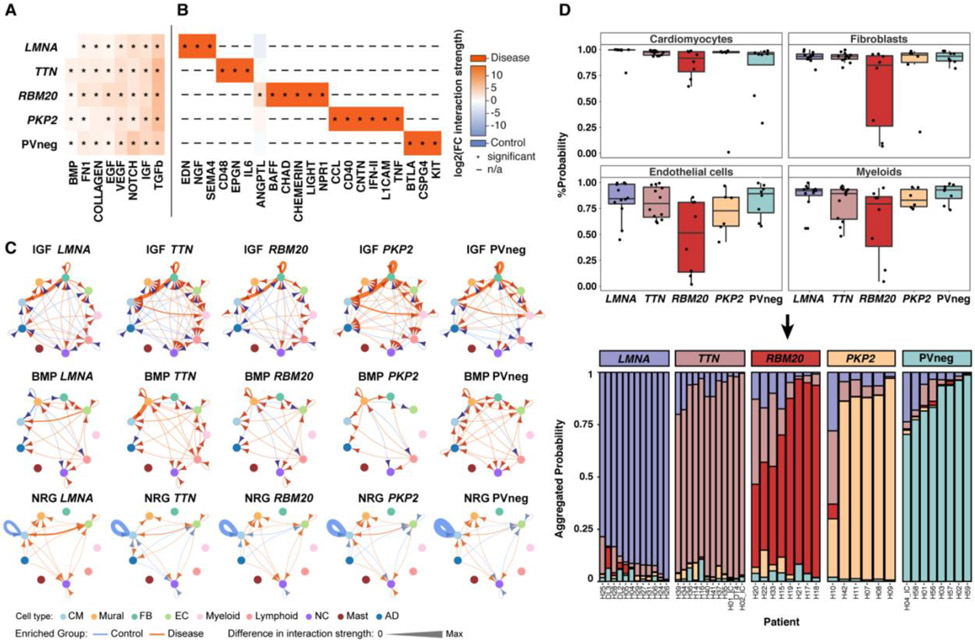
Altered cell-cell interactions and recognition of genotype-specific transcriptional responses. Heatmaps depict shared (**A**) and unique (**B**) signaling pathways in LVs, with significantly different expression in genotypes compared to controls. Signaling pathways are defined in the CellChat database (75). Changes in interaction strength (log2(fold-change)), scaled by color intensity (red, increased; blue, decreased). *denotes significance; adjusted *p*-values≤0.05; n/a denotes expression not detected in control or disease. (**C**) Circle plots of significant (adjusted *p*-value≤0.05) cell-cell communication depict differentially regulated IGF, BMP, and NRG pathways and interactions in disease LVs. The line thickness denotes interaction strength of signals from sending and receiving cell; with color scaled from zero to maximum in disease versus controls (orange, increased; blue, decreased). Arrows indicate directionality. (**D**) (Top) Genotype prediction probability from graph attention networks (GAT) per cell type. (Bottom) Stacked bar plots represent the likelihood (% aggregated probability) of individual patient genotypes by GAT prediction. The vast majority of established genotypes were predicted with high probability, with lower prediction probability only in H10 (*PKP2*), H20 (*RBM20*), H22 (*RBM20*) and H33 (*RBM20*).

We detected aberrant intercellular signaling across disease (Figs. 6A, S40), including upregulation of BMP, FN1, Collagen, EGF, IGF and TGF pathways that promote fibrosis. Signaling dependent on VEGF, NOTCH and ANGPT was also increased in disease, implying vascular remodeling. Strikingly, genotype-selective increases in intercellular signaling pathways were also identified in LVs (Figs. 6B, S40A, B): EDN in *LMNA*, the proinflammatory IL6 in *TTN*, BAFF/LIGHT (denoting TNF signaling) in *RBM20*, pro-inflammatory CCL and TNF in *PKP2*, and the immune modulator BTLA in PVneg. Some of these intercellular signaling pathways were similarly dysregulated in RV, but chamber-specific changes were also observed (Figs. S40C, D, S41).

We also identified genotype-specific differences in the cells sending and receiving signals in disease pathways (Figs. 6C). For example, IGF signaling (Fig. S42), crucial in cell growth and CM hypertrophy, showed increased FB autocrine (highest in *PKP2*) and paracrine FB to CM signaling, paralleling findings in experimental models (76, 77). In addition, IGF signaling from myeloid cells to CMs only occurred in *TTN*, *PKP2* and PVneg, an interaction that might promote muscle repair (78).

The source of BMP signaling changed in a genotype-specific manner (Figs. 6C, S43). BMP signaling from MC to CM was increased in *TTN* LVs and RVs but was downregulated in PVneg and *LMNA* LVs and other RVs (Figs. S41, S43). Notably, BMP signaling in ECs originated solely from EC7, likely depending on *BMP6* upregulation (Figs. 3E, G). EDN signaling from EC7 to CM and MC was highly genotype-selective, occurring in *LMNA* LV and *PKP2* RV (Fig. S44).

NRG signaling (comprising *NRG1-3* and *ERBB* receptors) showed multifaceted changes. Disease LVs markedly attenuated autocrine NRG signaling in CMs, while EC and CM signaling was upregulated (highest in *LMNA*) in all genotypes except *RBM20* (Fig. 6C, S45). Additionally, NRG3-ERBB4 interactions identified in controls shifted in disease to NRG1-ERBB4 and NRG1-ERBB3 in a genotype-specific manner, consistent with changes in *NRG1/3* expression in EC7 (Figs. 3G, S46) (79). This predicted NRG/ERBB shift may provide compensatory responses to adverse remodeling in cardiomyopathies (48).

### Graph attention networks recognized genotype-specific expression patterns

We applied machine learning approaches to snRNAseq data to further advance the recognition of cell and genotype-specific transcriptional patterns. Cell-specific neighborhood graphs showed more connectivities among single-nuclei transcriptomes from PVneg hearts and hearts with PVs in the same gene, compared to PVs from different genes (Fig. S47). Subsequently, we generated a graph attention network (GAT) for multinomial classification of genotypes trained on four major informative LV cell types: CMs, FBs, ECs, and myeloids (Fig. S47A-B). The GAT predicted *LMNA*, *TTN*, *RBM20*, *PKP2*, and PVneg genotypes with high accuracy. Among LV samples, the genotype prediction accuracy differed by cell types: CMs: 0.93, FBs: 0.92, myeloids: 0.85, ECs: 0.79 (Figs. 6D, S48, Tables S69-70; corresponding RV data Fig. S47C). Aggregation of genotype predictions obtained from these four LV cell types strengthened the correct prediction of genotypes, resulting in a high confidence model (Fig. 6D). Of note, three (H10, H22 and H33) of the four lower prediction probabilities occurred in samples with both a primary and secondary PVs, assigned as such by prior genotype and clinical phenotype review (Table S1). Moreover, as this machine learning model independently confirmed a genotype- and cell type-specific transcriptional signature, we concluded that these snRNAseq datasets accurately described the molecular responses to PVs and unexplained causes of DCM and ACM.

## Discussion

Our analyses of snRNAseq of LV and RV samples illuminated the cell types and states, molecular signals, and predicted intercellular communications that characterized DCM and ACM. In comparison to control hearts, we identified differences at multiple levels, including changes in the proportions of cell types and states, additional cell states, and differential gene expression, substantially expanding earlier insights achieved by bulk tissue analyses. Across all genotypes, disease hearts demonstrated some common dissimilarities from control hearts, often with graded differences between LVs and RVs. Despite studying hearts from patients with advanced disease who received diverse therapies, congruent transcriptional signatures emerged for different PVs within the same gene and varied between genotypes.

The differences between genotype groups and controls reflected differences in mean expression and not differences in variance. This was true for each genotype group including PVneg. Transcriptional signatures were complex, diversified both by the proportions of canonical and stressed cell states and by differentially expressed genes within the same states. While interrogation of these datasets provides ongoing opportunities for discovery, our findings provided substantial evidence that genotype influenced pathological remodeling of the heart. These results upend a prevalent dogma that HF results from a common final pathway and can guide the future development of therapies with selective targets to enhance personalized medicine.

Despite anatomical and histopathological differences between DCM and ACM, we identified shared changes in the cellular composition of ventricular tissues, albeit with DCM LV features largely mirrored in ACM RVs. Cardiomyopathies were depleted in CMs while EC and immune cell populations were increased. FBs did not expand, but increased states that augmented ECM gene expression and collagen deposition. Based on these changes and cytokine profiles (TGFβ activation, increased IGF signaling, decreased *CCL2* expression; Figs. 2H-I, 6C), we predicted cell-cell interactions and key molecules that are appropriate for mechanistic studies to causally link differential expression with adverse cardiac remodeling.

Disease CMs exhibited loss of the canonical state vCM1.0, and the emergence of genotype-enriched cell states with DEGs. Many of these responses are associated with stress-induced contractile, metabolic, and electrophysiologic properties that are prominent clinical manifestations of some genotypes. For example, attenuated expression of *SMYD1*, a critical organizer of sarcomere structure, epigenetic and metabolic remodeling, occurred only in CMs with PVs, and was unaltered in PVneg samples. With its global impact on myocardial function, dysregulation of *SMYD1* might contribute to earlier presentation and poorer outcomes of cardiomyopathy patients with, versus without, PVs (80). *LMNA* CMs had the greatest expansion of vCM1.2, enriched for genes encoding Ca^2+^ regulators and molecules with electrophysiologic functions, while *RMB20* and *LMNA* genotypes had the highest expression of *MYL4*, a sarcomere protein associated with atrial fibrillation. These data suggested molecular mechanisms whereby particular genotypes convey increased risks for arrhythmias and sudden cardiac death in patients.

Shifts in FB states explained the paradoxically increased fibrosis in cardiomyopathies, without expansion of overall FB populations. DCM LVs and ACM RVs showed increased proportions of vFB2, enriched for genes that modulate ECM composition, turnover, stiffness and fibrotic scarring (81) and reciprocally decreased proportions of vFB3 that express transcripts suppressing fibrosis. Notably, fibrogenic genes within vFB2 were highly expressed in *RBM20*, a genotype within our cohort with the poorest ventricular function and youngest age for HF diagnosis and cardiac transplantation. Strategies to manipulate proteins encoded by these genes may attenuate the prominent myocardial fibrosis that characterizes cardiomyopathies.

Unexpectedly, mural cells (PCs, SMCs) showed no increase compared to ECs across cardiomyopathies. DEG analysis suggested that these cells promoted vascular remodeling and dysfunction. PCs diminished *PDGFRB* expression and showed aberrant NOTCH signaling in vascular beds, while genotype selective DEGs in SMCs upregulated contractile genes, augmenting fiber formation. Together these molecular signals may contribute to microvascular dysfunction that occurs in cardiomyopathy patients (82) and adversely influences ventricular performance.

Among EC, EC7 demarcated the endocardium and had the most DEGs in disease compared to control. Little is known about this myocardial layer in HF pathogenesis. The endocardium forms via dynamic regulation of NOTCH, neuregulin (83) and BMP (84) signals. Pediatric heart diseases can exhibit pathological expansion of the endocardium (denoted endocardial fibroelastosis) which diminishes cardiac performance and is associated with progression to HF (84). Our data suggested similar dysregulation of these molecular pathways in adult-onset cardiomyopathies with increased *BMP6* and *NRG1* in EC7 and decreased *INHBA* (53). These signals may cause pathological changes in endocardium in DCM and ACM hearts and contribute to myocardial dysfunction.

Diseased hearts increased myeloid cells. Expansion of immune cell populations might arise from recruitment of circulating cells or proliferation of resident cells. In our samples proliferating myeloid cells decreased, as previously observed in other DCM studies (85). Surprisingly, myeloid recruitment via the CCR2/CCL2 axis, primarily mediated via vFB3, was decreased in all samples, with particularly dysregulated fibroblast-to-macrophage interactions in *TTN*. Although distinguishing between proliferation versus recruitment late in disease was problematic, previous studies indicate peak cytokine expression precedes the emergence of HF (86, 87). Analyses of earlier time points during disease progression will be important to discern these key signals.

To confirm and expand the conclusion that genotype-specific signals meaningfully contributed to disease pathogenesis, we employed machine learning strategies. Using GAT to classify patients’ genotypes from each cell type, we found that CMs, FBs, ECs, and myeloid cells provided the highest and most discriminatory information. Harnessing LV and RV data from these cell types, we independently predicted the established genotype of each patient with high accuracy. Moreover, among the four samples with the lowest genotype predictive probability, three samples carried two PVs, indicating that the model detected subtle transcriptional differences with additional influences.

While expanding machine learning models to much larger datasets will undoubtedly improve accuracy, these early analyses supported the conclusion that PVs in different genes evoked cell type and state-specific responses that altered inter-cellular communications and promoted distinct disease pathways. We recognize that pathways may converge, but even in advanced disease, our data indicated that genotypes promoted specific transcriptional signals that likely contributed to distinct as well as common manifestations of genetic cardiomyopathies.

Future studies are needed to comprehensively define the molecular pathophysiology of cardiomyopathies and HF, including assessments of age, sex, and ancestral influences, other DCM and ACM genotypes, additional cardiac regions, and longitudinal analyses to identify initiating and secondary processes. We also expect that the deployment of strategies that upsample conduction system and other rare cell types, and incorporation of techniques to characterize the epigenome, proteome, and spatial relationships between cell types, states, and gene expression will also be highly informative. To promote these initiatives, we freely provide all datasets and an interactive platform (https://cellxgene.cziscience.com/collections/e75342a8-0f3b-4ec5-8ee1-245a23e0f7cb) with cell type and state annotations. We expect that these resources will advance mechanistic studies to improve treatments of cardiomyopathies and enable HF prevention strategies.

## Materials and methods

Detailed information on human subject studies, experimental methods, data access, codes, algorithms, and computational programs used in this manuscript is provided in the Supplementary Materials (89).

Human studies were performed using protocols that were reviewed and approved by the ethics boards of participating institutions. DCM and ACM ventricular samples were collected from genotyped patients undergoing mechanical support (n=15) or heart transplantation (n=31) and from deceased donors with non-failing hearts as previously described (4). Nuclei from full-thickness LV and RV regions were isolated and processed for snRNAseq as described (4). Data were mapped to the human genome (GRCh38), processed to remove doublets and identify nuclei that met high quality standards, and harmonized to remove batch effects (105). Manifolds were constructed using Uniform Manifold Approximation and Projections (UMAPs) for all and individual cell types.

Differential abundance analyses of cell types and states were performed using centered log ratio transformation including a linear model. Differential gene expression between disease and control tissues were deduced using a pseudobulk approach and EdgeR (109, 110). Comparative analyses assessed cell type and cell state abundances and differential gene expression between disease samples and controls and between genotypes. Selected genes with differential expression were validated using single molecule fluorescent *in situ* hybridization or quantitative immunohistochemistry.

We investigated cell-cell communication using CellChat (75). The expression of genes previously identified through genome wide-association studies of DCM was assessed in diseased and control tissues. We interrogated transcriptional datasets with machine learning tools to generate cell type features that distinguished PVs within the same gene from PVs within different genes and used these data to generate a graph attention network (GAT). The accuracy for GAT analyses of randomly selected patient data to assign the correct, clinically assigned genotype was assessed.

## Supplementary Material

Supplemental Materials

Supp. table 1

Supp table 2

Supp table 3

Supp table 4

Supp table 5

Supp table 6Supp table

Supp table 7

Supp table 8

Supp table 9

Supp table 10

Supp table 11.2

Supp table 11.3

Supp table 11.1

Supp table 11.4

Supp table 11.5

Supp table 11.6

Supp table 11.8

Supp table 11.9

Supp table 11.7

Supp table 11.10

Supp table 11.11

Supp table 11.12

Supp table 11.13

Supp table 11.14

Supp table 11.15

Supp table 11.17

Supp table 11.16

Supp table 11.18

Supp table 12.2

Supp table 12.3

Supp table 12.1

Supp table 12.4

Supp table 12.5

Supp table12.6

Supp table 12.7

Supp table 12.8

Supp table 12.9

Supp table 12.10

Supp table 12.11

Supp table 12.12

Supp table 12.13

Supp table 12.14

Supp table12.15

Supp table 12.16

Supp table 12.17

Supp table 12.18

Supp table 13

Supp table 14

Supp table 15

Supp table 16

Supp table 17

Supp table 18

Supp table 19

Supp table 20.1

Supp table 20.3

Supp table 20.2

Supp table 20.5

Supp table 20.4

Supp table 20.6

Supp table 20.8

Supp table 20.7

Supp table 20.9

Supp table 20.10

Supp table 20.11

Supp table 20.12

Supp table 21.1

Supp table 21.2

Supp table 21.3

Supp table 21.4

Supp table 21.5

Supp table 21.6

Supp table 21.7

Supp table 21.9

Supp table 21.8

Supp table 21.10

Supp table 21.11

Supp table 21.12

Supp table 22

Supp table 23

Supp table 24

Supp table 25

Supp table 26

Supp table 27.3

Supp table 27.1

Supp table 27.2

Supp table 27.4

Supp table 27.5

Supp table 27.6

Supp table 27.7

Supp table 27.8

Supp table 27.9

Supp table 27.10

Supp table 27.11

Supp table 27,12

Supp table 28.1

Supp table 28.2

Supp table 28.3

Supp table 28.4

Supp table 28.5

Supp table 28.6

Supp table 28.7

Supp table 28.8

Supp table 28.9

Supp table 28.10

Supp table 28.11

Supp table 28.12

Supp table 29

Supp table 30

Supp table 31

Supp table 32

Supp table 33

Supp table 34.1

Supp table 34.2

Supp table 34.3

Supp table 34.4

Supp table 34.5

Supp table 34.6

Supp table 34.7

Supp table 34.8

Supp table 34.9

Supp table 34.10

Supp table 34.11

Supp table 34.12

Supp table 35.1

Supp table 35.2

Supp table 35.3

Supp table 35.6

Supp table 35.4

Supp table 35.5

Supp table 35.7

Supp table 35.8

Supp table 35.9

Supp table 35.10

Supp table 35.11

Supp table 35.12

Supp table 36

Supp table 37

Supp table 38

Supp table 39

Supp table 40

Supp table 41

Supp table 42

Supp table 43

Supp table 44.2

Supp table 44.3

Supp table 44.1

Supp table 44.4

Supp table 44.5

Supp table 44.6

Supp table 44.7

Supp table 44.8

Supp table 44.9

Supp table 44.10

Supp table 44.11

Supp table 44.12

Supp table 44.13

Supp table 44.14

Supp table 44.15

Supp table 44.16

Supp table 44.17

Supp table 44.18

Supp table 44.19

Supp table 44.20

Supp table 44.21

Supp table 44.22

Supp table 44.23

Supp table 44.24

Supp table 44.25

Supp table 44.26

Supp table 45.1

Supp table 45.3

Supp table 45.2

Supp table 45.4

Supp table 45.5

Supp table 45.6

Supp table 45.7

Supp table 45.8

Supp table 45.9

Supp table 45.11

Supp table 45.10

Supp table 45.12

Supp table 45.13

Supp table 45.14

Supp table 45.15

Supp table 45.16

Supp table 45.17

Supp table 45.18

Supp table 45.19

Supp table 45.20

Supp table 45.21

Supp table 46

Supp table 47

Supp table 48

Supp table 49

Supp table 50

Supp table 51.2

Supp table 51.1

Supp table 51.3

Supp table 51.4

Supp table 51.5

Supp table 51.6

Supp table 51.7

Supp table 51.8

Supp table 51.9

Supp table 51.10

Supp table 51.11

Supp table 51.13

Supp table 51.12

Supp table 51.14

Supp table 51.15

Supp table 51.16

Supp table 52.2

Supp table 52.3

Supp table 52.1

Supp table 52.5

Supp table 52.4

Supp table 52.6

Supp table 52.7

Supp table 52.8

Supp table 52.9

Supp table 52.10

Supp table 52.11

Supp table 52.12

Supp table 52.14

Supp table 52.13

Supp table 52.15

Supp table 52.16

Supp table 53

Supp table 54

Supp table 55

Supp table 56

Supp table 57

Supp table 58.3

Supp table 58 1

Supp table 58.2

Supp table 58.4

Supp table 58.5

Supp table 58.6

Supp table 58.7

Supp table 58.9

Supp table 58.8

Supp table 59.2

Supp table 59.1

Supp table 59.3

Supp table 59.4

Supp table 59.5

Supp table 59.6

Supp table 60

Supp table 61

Supp table 62

Supp table 63

Supp table 64

Supp table 65.1

Supp table 65.2

Supp table 65.3

Supp table 65.4

Supp table 66.1

Supp table 66.2

Supp table 67

Supp table 68

Supp table 69

Supp table 70

Supp table 71

Supp tables Index

## Data Availability

All sequence data generated and analyzed in this study has been deposited as BAM files at the European Genome-phenome Archive (EGA), which is hosted by the EBI and the CRG, under accession number EGAS00001006374 (https://ega-archive.org/studies/EGAS00001006374). Further information about EGA can be found on https://ega-archive.org, "The European Genome-phenome Archive in 2021" (https://academic.oup.com/nar/advance-article/doi/10.1093/nar/gkab1059/6430505). Processed data can be analyzed using the Cellxgene tool website: https://cellxgene.cziscience.com/collections/e75342a8-0f3b-4ec5-8eel-245a23e0f7cb/private and are available in h5ad-format to download. Metadata sheets and patient information are available in Table S1&2. All code used to generate the figures in this publication are available on github (https://github.com/heiniglab/DCM_heart_cell_atlas). Code used to reproduce the presented analyses is indexed on Zenodo (88). All scripts run on jupyter notebooks are available as “.ipynb” files, scripts executed in command line are available as .txts or .sh files. R scripts are available as “.R”. Anaconda environments are available as yml files containing information on package versions.
