# Supplementary material for: Pathogenic variants damage cell composition and single cell transcription in cardiomyopathies
Source: Science. Author manuscript; Available in PMC 2022 Oct 3. (PMC9528698; doi:10.1126/science.abo1984)
Supplement: Supplemental Materials [file NIHMS1835045-supplement-Supplemental_Materials.pdf]

Supplementary Materials for

Pathogenic variants damage cell compositions and single cell transcription in cardiomyopathies

Authors: Daniel Reichart[1,2,3#], Eric L. Lindberg[4#^], Henrike Maatz[4,5#], Antonio M.A. Miranda[6,7], Anissa Viveiros[8,9], Nikolay Shvetsov[4], Anna Gärtner[10], Emily R. Nadelmann[1], Michael Lee[6], Kazumasa Kanemaru[11], Jorge Ruiz-Orera[4], Viktoria Strohmenger[1,12], Daniel M. DeLaughter[1,13], Giannino Patone[4], Hao Zhang[8,9], Andrew Woehler[14], Christoph Lippert[15,16], Yuri Kim[1,2], Eleonora Adami[4], Joshua M. Gorham[1], Sam N. Barnett[6], Kemar Brown[1,17], Rachel J. Buchan[6,18], Rasheda A. Chowdhury[6], Chrystalla Constantinou[6], James Cranley[11], Leanne E. Felkin[6,18], Henrik Fox[19], Ahla Ghauri[20], Jan Gummert[19], Masatoshi Kanda[4,21], Ruoyan Li[11], Lukas Mach[6,18], Barbara McDonough[2,13], Sara Samari[6], Farnoush Shahriaran[22], Clarence Yapp[23], Caroline Stanasiuk[10], Pantazis I. Theotokis[6,24], Fabian J. Theis[22], Antoon van den Bogaerdt[25], Hiroko Wakimoto[1], James S. Ware[6,18,24], Catherine L. Worth[4], Paul J.R. Barton[6,18,24], Young-Ae Lee[20,26], Sarah A. Teichmann[11,27], Hendrik Milting[10#], Michela Noseda[6,7#], Gavin Y. Oudit[8,9#], Matthias Heinig[22,28,29#], Jonathan G. Seidman[1#^], Norbert Hubner[4,5,30#^], Christine E. Seidman[1,2,12#^]

Affiliations:

1. Department of Genetics, Harvard Medical School, Boston MA, 02115 USA
2. Cardiovascular Division, Brigham and Women's Hospital Boston MA, 02115 USA
3. Department of Medicine I, University Hospital, LMU Munich, 80336 Munich, Germany
4. Cardiovascular and Metabolic Sciences, Max Delbrück Center for Molecular Medicine in the Helmholtz Association (MDC), 13125 Berlin, Germany
5. DZHK (German Centre for Cardiovascular Research), Partner Site Berlin, 10785 Berlin, Germany
6. National Heart and Lung Institute, Imperial College London, London SW3 6LY, UK

7. British Heart Foundation Centre for Research Excellence and Centre for Regenerative Medicine, Imperial College London WC2R 2LS, UK
8. Division of Cardiology, Department of Medicine, Faculty of Medicine and Dentistry, University of Alberta, Edmonton, Alberta T6G 2R3, Canada
9. Mazankowski Alberta Heart Institute, Faculty of Medicine and Dentistry, University of Alberta, Edmonton, Alberta T6G 2R3, Canada
10. Erich and Hanna Klessmann Institute, Heart and Diabetes Center NRW, University Hospital of the Ruhr-University Bochum, 32545 Bad Oeynhausen, Germany
11. Cellular Genetics Programme, Wellcome Sanger Institute, Wellcome Genome Campus, Hinxton CB10 1SA, UK
12. Walter-Brendel-Centre of Experimental Medicine, Ludwig-Maximilian University of Munich, 81377 Munich, Germany
13. Howard Hughes Medical Institute, Bethesda MD, 20815-6789, USA
14. Systems Biology Imaging Platform, Berlin Institute for Medical Systems Biology (BIMSB), Max-Delbrück-Center for Molecular Medicine in the Helmholtz Association (MDC), 10115 Berlin, Germany
15. Digital Health-Machine Learning group, Hasso Plattner Institute for Digital Engineering, University of Potsdam, 14482 Potsdam, Germany
16. Hasso Plattner Institute for Digital Health, Icahn School of Medicine at Mount Sinai, NY 10029, USA
17. Cardiac Unit, Massachusetts General Hospital, Boston, MA 02114, USA
18. Royal Brompton and Harefield Hospitals, Guy's and St. Thomas' NHS Foundation Trust, London SW3 6NR, UK
19. Heart and Diabetes Center NRW, Clinic for Thoracic and Cardiovascular Surgery, University Hospital of the Ruhr-University, 32545 Bad Oeynhausen, Germany
20. Max Delbrück Center for Molecular Medicine in the Helmholtz Association (MDC), 13125 Berlin, Germany
21. Department of Rheumatology and Clinical Immunology, Sapporo Medical University School of Medicine, Sapporo 060-8556, Japan
22. Computational Health Center, Helmholtz Zentrum München Deutsches Forschungszentrum für Gesundheit und Umwelt (GmbH), 85764 Neuherberg, Germany

23. Laboratory of Systems Pharmacology, Harvard Medical School, Boston, MA 02115, USA
24. MRC London Institute of Medical Sciences, Imperial College London, London W12 0NN, UK
25. ETB-Bislife Foundation, POB 309, 2300 AH Leiden, The Netherlands.
26. Clinic for Pediatric Allergy, Experimental and Clinical Research Center, Charité-Universitätsmedizin Berlin, 13125 Berlin, Germany
27. Department of Physics, Cavendish Laboratory, University of Cambridge, Cambridge CB3 0HE, UK
28. Department of Informatics, Technische Universitaet Muenchen (TUM), 85748 Munich, Germany
29. DZHK (German Centre for Cardiovascular Research), Munich Heart Association, Partner Site Munich, 10785 Berlin, Germany
30. Charité-Universitätsmedizin Berlin, 10117 Berlin, Germany

# Denotes equal contribution
^ Corresponding authors: Eric L. Lindberg: eric.lindberg@mdc-berlin.de
Jonathan Seidman: seidman@genetics.med.harvard.edu
Norbert Hubner: nhuebner@mdc-berlin.de
Christine Seidman:cseidman@genetics.med.harvard.edu

This PDF file includes:

Materials and Methods
Figs. S1 to S53
Index to Tables S1 to S71 (provided in Excel format)
References 90-129, cited here, are provided in main manuscript

Materials and Methods

Data reporting

Data objects with the raw counts matrices and annotation are available via cellxgene through the Human Cell Atlas (HCA) Data Coordination Platform (DCP). Raw data for all samples, including the five major genotypes (*LMNA, RMB20, TTN, PKP2* and PVneg) and rare genotypes (*BAG3, DES, DSP, FKTN, TNNC1, TNNT2, TPMI, PLN*), are available through the European Genome Archive. All of our data can be explored at https://cellxgene.cziscience.com/collections/e75342a8-0f3b-4ec5-8ee1-245a23e0f7cb/private. All methods refer to the analyses of the five major genotypes unless differently stated.

Ethics statement

Clinical details on cardiac tissues are provided on Table S1. Control hearts were obtained from unused transplant organ donations, including 12 previously described samples (*4*), and six additional control samples from the Bad Oeynhausen Heart Center and NHS Blood and Transplant Health Authority. Discarded disease heart samples were obtained in the context of clinical patient care. All cardiac tissues were anonymized and used with approved protocols reviewed by the ethics committees listed below:
a) Bad Oeynhausen Heart Center; Ethics Board of the Ruhr-University Bochum (Approvals 2020-640-1; 21/2013)
b) Mazankowski Alberta Heart Institute (MAHI, Edmonton, Canada); Human Explanted Heart Program (HELP, Pro00011739)
c) Cardiovascular Research Centre Biobank at the Royal Brompton and Harefield Hospitals, Guy's and St. Thomas' NHS Foundation Trust (EC reference 09/H0504/104 +5)
d) Imperial College (REC reference16/LO/1568)
e) Mass General Brigham Human Research Protection Committee (Protocol 1999P010895)
f) Harvard Longwood Campus Institutional Review Board (Protocol M11135)

Cohort samples

Control heart samples were collected as previously described (*4*). Disease samples were collected from cardiomyopathy patients with heart failure, prior to mechanical support (n=15) or at the time of heart transplantation (n=31). All samples were full-thickness myocardial specimens from the LV and RV free walls, and the LV apex, and interventricular septum. Regions with large epicardial fat deposits or macroscopic areas of high fibrosis were intentionally excluded. When full-thickness apical cores were obtained, other regions were not available. Additional details are provided in Fig. S2A and Table S1. Thorough sample collection details have been described previously (*89*).

Patient genotyping

Genomic DNA from patient samples from the Bad Oeynhausen Heart Center was isolated from blood using the High Pure PCR Preparation Kit® (Roche Diagnostics GmbH) or Genomic DNA Extraction Kit (Qiagen). DNA was prepared for gene enrichment re-sequencing on a MiSeq® sequencing system using the TruSight™ Rapid Capture Sample Preparation Kit (Illumina). DNA was screened for pathogenic or likely pathogenic variants using the TruSight™ Cardio (174 genes) or the TruSight™ Cardiomyopathy (46 genes) Sequencing Panel (Illumina). In a subset of samples, whole exome or genome sequencing was performed (Table S1) and analyzed for cardiomyopathy genes. Variants were annotated using VariantStudio™ v.3.0 (Illumina) and classified according to the recommendations of the American College of Medical Genetics and Genomics and the Association for Molecular Pathology (ACMG) (*91*).

Genomic DNA from patients enrolled at Brigham and Women's Hospital and the Mazankowski Alberta Heart Institute was isolated using Genomic DNA Extraction Kit (Qiagen) from LV samples and whole exome sequencing (WES) performed using Illumina NovaSeq instruments. All sequencing reads were aligned to hg19 (GRCh37) using BWA-MEM (*92*). Single nucleotide variants (SNVs) and small indels were identified using the Genome Analysis Tool Kit (GATK; version 3.8) Haplotype Caller tool (*93*). The variant call format file was annotated using dbNSFP (*94*), gnomAD v2.1 (*95*), and SnpEff (version 4.3t, annotation database GRCh37.75). High quality variants (pass GATK, Variant Score Quality Recalibration (VSQR, truth sensitivity threshold 99.5 for SNVs, 99.0 for indels), a minimum depth (DP) of 10, and genotype quality (GQ) $\geq$ 20, and quality (QUAL) $\geq$ 30) were filtered for rare (defined as minor allele frequency < 1.00e-04 in gnomAD v2.1) pathogenic variants.

Genomic DNA from patients enrolled at the Imperial College was isolated from blood or heart tissues using the Genomic DNA Extraction Kit (Qiagen) and processed for cardiomyopathy panel sequencing or WES using Illumina NovaSeq instruments. Panel sequencing and bioinformatic analysis was performed as previously described (*96*). Reads were aligned to hg38 (GRCh38) using BWA-MEM. Variants were annotated using Ensembl VEP v99 (*97*), and CardioClassifier (*98*). Rare variants (MAF<0.001) in known disease genes were considered for a potential causal role in disease and interpreted using the ACMG-AMP variant interpretation framework (*91*).

Single nuclei isolation of cardiac samples and processing on the 10X Genomics platform

Samples were processed independent of disease, genotype, or control status. Nuclei isolation and library preparation was performed at the Harvard Medical School, Imperial College London, and the Max-Delbrück Center for Molecular Medicine as previously reported (*4*, *99*). Isolated nuclei from flash-frozen tissue were visually inspected under the microscope to assess nuclei integrity and manually or automatically counted using a Countess II (Life |Technologies). Nuclei suspension was loaded on the Chromium Controller (10X Genomics) with targeted nuclei recovery of 5,000–10,000 per reaction (Table S2).

3′ gene expression libraries were prepared according to the manufacturer's instructions of the v3 Chromium Single Cell Reagent Kits (10X Genomics). Quality control of final library cDNA was done using Bioanalyzer High

Sensitivity DNA Analysis (Agilent) and the KAPA Library Quantification kit. Libraries were sequenced on an Illumina HighSeq 4000 or NovaSeq with a targeted read number of 30,000-50,000 reads per nucleus (Table S2).

Data pre-processing and transcriptome mapping

Bcl files were converted to Fastq files by using bcl2fastq. Each sample was mapped to the human reference genome GRCh38 with a modified pre-mRNA gtf file of Ensembl release Ens84 (*100*) using the CellRanger suite (v.3.0.1) with specifications provided in the DCMheart github repository. Reads mapping within exonic and intronic regions were counted. Mapping quality was assessed using the cellranger summary statistics. Reads overlapping multiple sequence features have been discarded. Two libraries (Table S2) exceeded the read number per nucleus of 200,000 and were downsampled to 100,000 reads per nucleus using the DropletUtils R package on the molecule_info.h5 file (*101*).

Count data processing

Empty droplets (identified by Emptydrops, implemented in the CellRanger workflow) were removed, samples were assembled into an AnnData object by concatenating the filtered_feature_bc_matrix.h5, and metadata information was added.

Quality control, batch correction and clustering

Downstream analysis employed the concatenated filtered feature-barcode matrices, using R Seurat v4.0.2 and Python Scanpy v1.5.1 toolkits (*102*, *103*). Doublets were identified and filtered using Solo v0.3 per sample (*104*). Additionally, scrublet scores (v0.2.1) were calculated with prior z- or log-transformation as an independent doublet detection method (*105*). Single nuclei were filtered for counts ($300 \leq$ n_counts $\leq 15,000$), genes ($300 \leq$ n_genes $\leq 5,000$), mitochondrial genes (percent_mito $\leq 1\%$), ribosomal genes (percent_ribo $\leq 1\%$), and soft max score detected by Solo (solo_score ≤ 0.5).

No significant differences were identified in cell-type abundances or expression profiles between free-wall (FW), the apical core (AP) and septal (S) samples and thus these were merged and denoted as LV as previously described (*4*). After read count normalization and log-transformation, highly variable genes were selected. Effects of percentage of mitochondrial genes and total counts per nucleus were regressed out and values were scaled to unit variance. Principal components were computed and elbow plots were used to define the appropriate number of principal components for neighbor graph construction. Prior to manifold construction using UMAP, selected principal components were harmonized by using R Harmony (*106*) or Python Harmonypy with "Patient" as batch key (Table S2). Nuclei were clustered using the network-based Louvain and Leiden algorithms (*107*, *108*). Differential expressed genes per cluster were calculated using the Wilcoxon rank sum test. Clusters with high similarity in differentially expressed genes were merged.

Nuclei were classified for cell type or denoted unassigned (Fig. 1C). Subsequently nuclei were subclustered to identify cell-states. Despite prior doublet removal we identified during subclustering some droplets with chimeric transcriptional profiles. Whether these may represent real biology, background RNA noise (soup), or multiplets is unclear. We labeled these clustered "nuclei" as unassigned (Fig. 1C, n= 52981 "nuclei"; 6%). Additional filters are provided in scripts deposited at the DCMheart github. Lymphocytes were annotated initially by merging scRNAseq (*4*) with snRNAseq data using the scVI framework v0.9.0 to improve marker identification and annotation (*109*). Subsequently scRNAseq data were then removed for further downstream analyses.

Differential gene expression and variance analysis

Differentially expressed genes (DEG) per cell type and state were calculated using the implemented Wilcoxon rank-sum-test. Only genes with mean expression (log transformed and library size normalized counts) >0.0125 in the control and genotype group were tested for differential expression. Genes were called as differentially expressed with FDR<5% and |log2FC|>0.5. DEGs for rare cell states (>5 nuclei in at least 3 patients) have not been computed. For comparison of genotype specific effects, pseudobulks for each cell type and cell state per LV and RV sample were computed. Differential gene expression analysis on pseudobulk expression values were performed using edgeR, v3.28.1 (*110*, *111*).

We assessed differences in the variability across cells between patients with a specific PV or PVneg and controls, in each cell type and each anatomical region separately. Because the variance of UMI counts is strongly dependent on the mean expression level, we first applied a variance stabilizing transformation (*112*) similar to scTransform (*113*) on the 1000 most highly variable genes. We used the Pearson residuals of a negative binomial regression with explanatory variables: total read count of each cell and the fraction of mitochondrial reads. Next, we computed the variance of the Pearson residuals for each gene in each patient, anatomical region and cell type. Finally, we compared the variance for each gene between patients with a specific mutation (or PVneg) against the control group using a Wilcoxon rank sum test and corrected across all comparisons made using the Benjamini Hochberg method.

Differential abundance analysis

Compositional data analyses were performed to determine genotype specific differences in cell type abundances, excluding unassigned nuclei. Analyses of cell counts per cell type and cell counts per cell state within each cell type were performed separately in each anatomical region (LV and RV). To account for the compositional nature of the data count data were transformed using the centered log ratio (CLR) transformation. Counts of zero were assumed to be due to insufficiently deep sampling and therefore imputed using the method of multiplicative replacement (*114*). To assess statistical differences between groups of samples, for example all patients with a specific genotype vs. controls, a linear model of the CLR values was estimated as a function of the grouping encoded as an indicator variable and a t-test was performed to determine the significance of the regression coefficient. Differential

abundance of all cell types or states in each anatomical region was assessed separately between patients of each genotype and controls. In addition, all DCM patients were compared to controls. For the analysis of cell states within each cell type, only cell state counts assigned to each cell type were considered, effectively normalizing all cell states within a cell type to 100%. Samples with less than 10 cells per cell type were excluded from the cell state analysis. Before analyzing differential abundance between samples of different genotypes or diagnosis, we assessed whether samples from different anatomical regions show compositional differences using the region as a grouping variable in the model described above. The comparison of FW and AP showed no significant abundance changes (all FDR>5%, Fig. S2D). Therefore AP and FW were merged. Next, we also compared the merged AP and FW to SP. As no significant abundance changes were observed (all FDR>5%, Fig. S2E) AP, FW and SP were merged into LV.

We also employed CLR transformed cell type abundance to consider sex specific differences of cell type abundance, separately for LVs and RVs, between DCM (10 females, 29 males) and controls (7 females, 11 males). As explanatory variables we included the additive terms phenotype (control=0, DCM=1) and sex (female=0, male=1) as well as an interaction term (DCM and male = 1, others = 0). We tested whether the interaction term was different from 0, which would indicate a significant (FDR < 5%) sex specific difference. Using the same approach, we compared sex specific cell abundances between *LMNA* and control hearts, as only this genotype had sufficient samples from males (n=7) and female (n=5) patients.

In addition to CLR values, abundance differences were also reported as differences of mean percentages between groups (while using statistical significance from the CLR analysis). The proportional changes in mean percentages of control and disease samples were reported (Figs. 1D, S4, S8, S12, S17, S21, S26, S32, S35, S38). Positive values indicate higher abundance in the disease group. In addition to CLR values, log ratios of abundances for cell type (or state) pairs between genotypes and controls were ascertained and reported. CLR values are normalized to the geometric mean of all abundance values. For a more intuitive interpretation of the differential abundance results, we complemented the CLR analysis with an analysis of all pairwise cell type (respectively cell state) ratios. Based on the imputed abundances used for the CLR analysis we also computed differences in log ratios of counts of cell type c1 and cell type c2 between groups of patients as assessed in the CLR analysis. Specifically we tested whether log (c1/c2) in group 1 was equal to log (c1/c2) in group 2 using a t-test. All P-values were adjusted for multiple testing using the Benjamini and Hochberg method and only significant results are shown.

GOterm and pathway enrichment

GOterm enrichment analysis was performed using the web-tool Gprofiler2 with default settings (*115*). Enriched KEGG pathways were identified using 'Pathway Enrichment Analysis' from the R package 'ReactomePA' (*116*).

Gene set score enrichment

Enrichment of individual pathways was calculated using the score_genes functionality implemented in Scanpy on log-transformed and scaled counts (*102*) using reference gene sets. These included: Apoptosis (REACTOME_APOPTOSIS, M15303; https://reactome.org), OSM pathway (*4*), TGFB-stimulation (curated from (*30*) using ldFC>0.7 and FDR of 0.05), Endothelial-to-mesenchymal (EMT) and mesenchymal-to-endothelial (MET) (Table S36), Deathbase (*117*) cross-referenced with the gene ontology GO:0010942 and GO:0060548, and Antigen presentation (MHCII) score (*118*). Cell-cycle scoring was defined using the Scanpy function *score_genes_cell_cycle*.

Cell-cell interaction and differential connectome analysis

Cell-cell interactions between assigned cell states in LVs and RVs were inferred using *CellChat* (version 1.1.0 using the cell-cell interaction database) (*75*). The cellchat database is accessible at http://www.cellchat.org/cellchatdb/. Analyses were performed using the log-transformed normalized gene counts with default parameters, and population size was accounted for when calculating communication probabilities. Data from controls and genotypes were compared to identify significant changes.

Interaction heatmaps were generated using the table produced by the *rankNet()* function comparing each genotype to controls, which produced aggregated communication probabilities across all cell states for each signaling pathway and p-values. To address for multiple testing p-values were Bonferroni-adjusted using the *p.adjust()* function in the *R stats* package. The log2(fold change) in aggregated communication probability of genotype vs control was calculated and plotted on a heatmap using *heatmap.2()* from the *gplots* package (https://CRAN.R-project.org/package=gplots). Heatmap color scales depict 0.05 sized intervals, from -14 to 14.

Circle plots (Fig. 6C) showing pathway specific changes in interaction strength between cell types were generated by aggregating communication probabilities per cell state, while subsetting for a specified pathway using the *aggregateNet()* function. Communication probabilities were then aggregated for cell states of the same cell type using the *mergeInteractions()* function and plotted using *netVisual_diffInteraction()*. Chord plots showing cell state interactions for specific signaling pathways were generated using the *netVisual_aggregate()* function in *CellChat*.

Masson trichrome staining and collagen quantification via hydroxyproline

Control and disease LV and RV tissue were fresh-frozen in isopentane (ThermoFisher) at -80°C and OCT (VWR) embedded. Sections were cut (10 µm thickness) using a microtome, placed onto slides and processed using a standard Masson trichrome staining protocol.

Extracellular matrix was quantified within 50 mg of LV and RV (Table S15) tissue, by measuring the quantity of hydroxyproline, a common amino acid in mammalian collagens, as described (*119*).

Differential gene expression validated by single molecule fluorescent *in situ* hybridization with RNAscope probes

Fresh-frozen LVs or RVs obtained from controls or patients with PVs were fixed overnight in 4% paraformaldehyde solution and then placed in 30% sucrose in PBS until submerged. Tissues were embedded in OCT compound, sectioned to 5 µm thickness using a cryotome and mounted on Superfrost Plus slides. Slides were pre-treated and incubated with probes according to the manufacturer's protocol (RNAScope Multiplex Fluorescent Reagent Kit V2, ACDBiotechne), but used Protease IV for digestion. Positive and negative controls were performed for each tissue sample. RNAscope probes were multiplexed according to the manufacturer's protocol and run with positive and negative controls. Tissue sections were counterstained with DAPI (Wavelength: 358/461 nm) and WGA (WGA Alexa Fluor® 488 conjugate by Invitrogen, Wavelength: 495/519 nm; Biotium CF®633 WGA Wavelength: 630/650nm). Opal 520 (Wavelength: 494/525 nm), Opal 570 (Wavelength 550/570 nm), Opal 620 (Wavelength 588/616 nm), Opal 690 (Wavelength 676/694 nm) dyes (Akoya Bioscience) were conjugated to the RNAscope probes. Slides were imaged using one of three microscope platforms. The LSM710 confocal microscope (Zeiss, North American samples) was used with 25x or 40x oil immersion objectives (1.3 oil, DIC III). The SP8 confocal microscope (Leica, for samples obtained and processed in Germany) was used with a NA 1.4 63x oil immersion objective. The LSM780 confocal microscope (Zeiss, samples from the Imperial College) was used with 20x (0.8 NA) or 40x oil immersion objectives (1.3 NA). Spectral bleed-through was corrected using Fiji or Zeiss' Zen Black software according to the manufacturer's manual (*120*, *121*). Cell segmentation and transcript quantification/spot detection for cardiomyocytes was performed using Multiple-Choice Microscopy (MCMICRO, Fig. 2C) (*122*). Cells were segmented using a custom trained instance segmentation model, Cypository (*123*), based on the MaskRCNN resnet50 architecture (*124*) and pretrained on the COCO dataset (*125*). Briefly, images were manually annotated with two classes - cell membrane stained with wheat germ agglutinin and background consisting of all other areas. Annotated data was split into training, validation and test images in a 0.72:0.18:0.1 split. Training was performed with stochastic gradient descent with a learning rate of 0.005, momentum of 0.9 and weight decay of 0.0005. Cypository was deployed using MCMICRO (*122*) which is an end-to-end nextflow-based image analysis pipeline for tissue images. After cells were segmented as label masks, spot detection of RNA was performed in S3segmenter (*126*) by convolving a Laplacian of Gaussian (LoG) kernel over the image and identifying local maxima that had responses above a threshold. The number of spots per channel and mean intensity were then quantified on a single cell basis. In order to quantify *SMYD1* transcripts per CMs, control (n=2) and disease samples were analyzed. At least five images per sample were taken at different regions with 64 to 114 CMs per image. Other RNAscope quantifications (Figs. 2D, S6D) was performed manually in controls and disease samples using the H-score as described by ACDBio (ACDBiotechne).

Immunofluorescence Staining

Fresh LVs obtained from controls (n=5) or patients with PVs (n=5) were embedded in OCT compound, and sectioned at 5 µm thickness using a cryotome and mounted on Superfrost Plus slides. Slides were fixed in 4% paraformaldehyde solution and permeabilized with 0.1% Triton X-100 (Sigma-Aldrich). After several washing steps, slides were incubated in TrueBlack® Lipofuscin Autofluorescence Quencher (Biotium), then blocked in 4% Bovine Serum Albumin (BSA). Slides were incubated in primary antibodies for SMYD1 (Abcam, ab181372) and Cardiac Troponin T (cTnT) to identify cardiomyocytes (Invitrogen, MA5-12960) overnight at 4°C. Next, slides were incubated with anti-rabbit Alexa Fluor™ 568 (Invitrogen, Wavelength: 578/603 nm), and anti-mouse Alexa Fluor™ 647 (Invitrogen, Wavelength: 650/665 nm) to visualize SMYD1 and cTnT respectively. Tissue sections were counterstained with DAPI (Wavelength: 358/461 nm) and WGA (WGA Alexa Fluor® 488 conjugate by Invitrogen, Wavelength: 495/519 nm). Slides were mounted in ProLong™ Gold Antifade Mountant (Invitrogen) and coverslipped. Slides were imaged using the SP5 laser scanning confocal microscope (Leica) with a 100x immersion objective (1.4 NA). Spectral bleed-through was corrected using Fiji or Zeiss' Zen Black software according to the manufacturer's manual (*120*, *121*). SMYD1 quantification per CMs (cTnT staining) was performed by measuring the integrated density using Fiji, where a minimum of ten transmural images were quantified with a total of 97 to 174 CMs per sample.

Selection of GWAS candidate loci and assessment of expression in cell types

Table S65 provides the candidate genes residing in 15 previously identified DCM GWAS loci. Differential gene expression results were obtained from the edgeR analysis. For analyses of GWAS genes we applied a strict fold change cutoff of |log2FC|>1 for disease compared to controls, and FDR cutoff of 5%. In addition, we removed signals derived from ambient RNA, identified as transcripts from the top 30 genes with cell type specific expression and high technical noise for each cell type.

Cell type specific expression was defined by first computing the average UMI count per gene, per nucleus, per cell type. Next, these averages per gene were normalized to sum to 100% across cell types. Finally, genes were termed as specifically expressed if a cell type's average UMI fraction was >85%. To assess expression above background levels, background droplets that do not contain nuclei were identified by the cellranger pipeline.

Construction of graph attention network models

Cell-types were split into separate anndata files followed by library-size normalization and log-transformation per nucleus (barcode). Highly variable genes (HVGs) were selected based on mean expression and dispersion. Effects of percentage of mitochondrial genes and total counts per nucleus were regressed out and values were scaled to unit variance. kNN-neighbor graphs were computed (*sc.pp.neighbors*) on harmonized (Patient as batch

key) PCs per cell-type as edge input for graph attention (GAT) models, using classification model structure described above.

In order to recognize genotype specific transcriptional patterns, an aggregated GAT model was built consisting of the individual cell-type specific GAT models, where each model was trained per cell-type and validated on test data (not included in training). Model performance was evaluated by using an inductive learning policy using a leave-one-out cross-validation approach (LOO-CV) .

Training was stopped at the point when performance on the validation dataset did not improve to avoid overfitting (early stopping procedure). Subsequently, the genotype probability was assigned based on the transcriptional signature of each nucleus from one patient that was left out of the training, a process that was replicated to encompass all patients. Probabilities across all nuclei per cell-type were aggregated to obtain the genotype-likelihood for each patient (Fig. 6D). The final classification model was restricted to highly abundant cell types (CM, FB, EC and myeloid cells) in LV. Each cell type per genotype was given a weight, which was obtained by multiplying the true positive (TP) and 1-false positive (FP) rate.

GAT takes node (nuclei) features (*X*, anndata.X) and the adjacency matrix (*A*) of the nodes as input features (Fig. S53). $X = \{x_1, x_2, x_3, \dots, x_N\}$, $x_i \in R^F$ where *N* is the number of nodes, and F is the number of HVGs for each node. In the first step the input files were forwarded to the GAT layer. As output, the learned graph representation was received (*H'*). Secondly, layer normalization (*LN*) and Exponential Linear Unit activation function (ELU) was applied to the learned graph representation. LN is defined as

$$LN(x) = \frac{x - E[x]}{\sqrt{Var[x] + \epsilon}}$$

and the ELU activation function is

$$ELU(x) = \{x, \qquad \alpha * (e^x - 1), \qquad x > 0\ x \leq 0\ ,$$

where $\alpha = 1$.

Next, a self-attention layer (*F'*) was applied (*127*) and the procedures from the previous step (*LN* and *ELU*) were repeated. Hidden features were extracted from the graph representation. *H'* and output from *F'* were concatenated to feed into the second GAT layer. Finally, a log-softmax function for multiclass classification was executed.

$$LogSoftmax(x_i) = log\ log\ \left(\frac{e^{x_i}}{\sum_j \quad e^{x_j}}\right)$$

For multiclass classification a negative log-likelihood was used as a loss function.

The individual GAT layers were built as described in (*128*); in brief:

1) Apply a linear transformation — Weighted matrix W to the feature vectors of the nodes.
where W is an earnable weight matrix and h_i a lower layer embedding.

$$z_i^{(l)} = W^{(l)} h_i^{(l)}$$

2) Attention Coefficients determine the relative importance of neighboring features to each other. These were calculated using the formula.

$$\gamma_{ij}^{(l)} = LeakyReLU\left(a^{(l)^T}\left[z_i^{(l)} \mid z_j^{(l)}\ \right]\right)$$

First, the z embeddings of the two nodes are concatenated, where || denotes concatenation. Then, it takes the dot product of concatenation and a learnable weight vector a. In the end, a LeakyReLU was applied to the result of the dot product. The attention score indicates the importance of a neighbor node in the message passing framework.

3) Normalization of attention coefficients by applying a softmax to normalize the attention scores on each node's incoming edges. *Ni* denotes the set of indices of neighbors of a node with index *i*.

$$\alpha_{ij}^{(l)} = \frac{e^{\gamma_{ij}^{(l)}}}{\sum_{k \in N(i)} \ e^{\gamma_{ik}^{(l)}}}$$

4) Computation of final output features

$$h_i^{(l+1)} = \sigma\left(\sum_{j \in N(i)} \alpha_{ij}^{(l)} z_j^{(l)}\right)$$

5) Computation of multiple attention mechanisms, improving stability of the learning process:

$$h_i' = \sigma\left(\frac{1}{K}\sum_{k=1}^{K} \sum_{j\in N(i)}^{N} \alpha_{ij}^{k} W^{k} h_j'\right)$$

where K denotes the number of independent attention maps used.

To derive the final prediction per patient, an aggregation procedure was used in which median prediction scores per cell-type per patient were multiplied with a pre-computed weight matrix. Prediction scores per cell-type equals the relative abundance of nuclei assigned to a genotype. The weight matrices were created from previously computed confusion matrices. The aggregation procedure was defined as,

$$P = \sum_{i=1}^{n} \hat{y}_i W_i$$

where P denotes the probability per genotype, $\hat{y}$ the probability of a cell-type to be derived from a patient with a certain genotype. W is the pre-computed weight matrix. n equals the number of cell-types included in the aggregation procedure. The weighted aggregation procedure yielded more robust results than mean value per class, as errors per classifier were compensated.

Alternative modeling

For alternative modeling, six different models were used, and accuracy and F1 macro on cross validation was compared. An example for fibroblasts is below (Table S71). Model performance was measured on the cell-type level. For input data we used the gene expression data matrix (anndata.X) and meta information about patients (such as age, gender). Hyperparameters for a) Random Forest, b) XGBOOST and c) KNN classifiers were selected according to GridsearchCV. The training procedure was based on cross validation with patient stratification, to avoid overfitting of patient specific transcriptional patterns. The best result by alternative methods was obtained by the Random Forest Classifier with an accuracy of 0.39 and F1 macro score 0.15. In addition to three classical machine learning approaches, three approaches based on neural networks were compared. i) Feed Forward Neural Network (FFNN) on the count matrix with three hidden layers obtained a lower performance on validation data. ii) SCANVI (single-cell ANnotation using Variational Inference), developed for single-cell annotation using variational inference (*129*), was outperformed by GAT in the unbiased genotype classification task. iii) An additional FFNN on graph embeddings was applied. The neighbors' information from the graph (considering first and second neighbors order) and edge quantity were extracted using a graph neural network embedding and used as

input features. This approach performed better than other neural networks and the classical machine learning approaches, however, was still outperformed by GAT (accuracy and F1 macro).

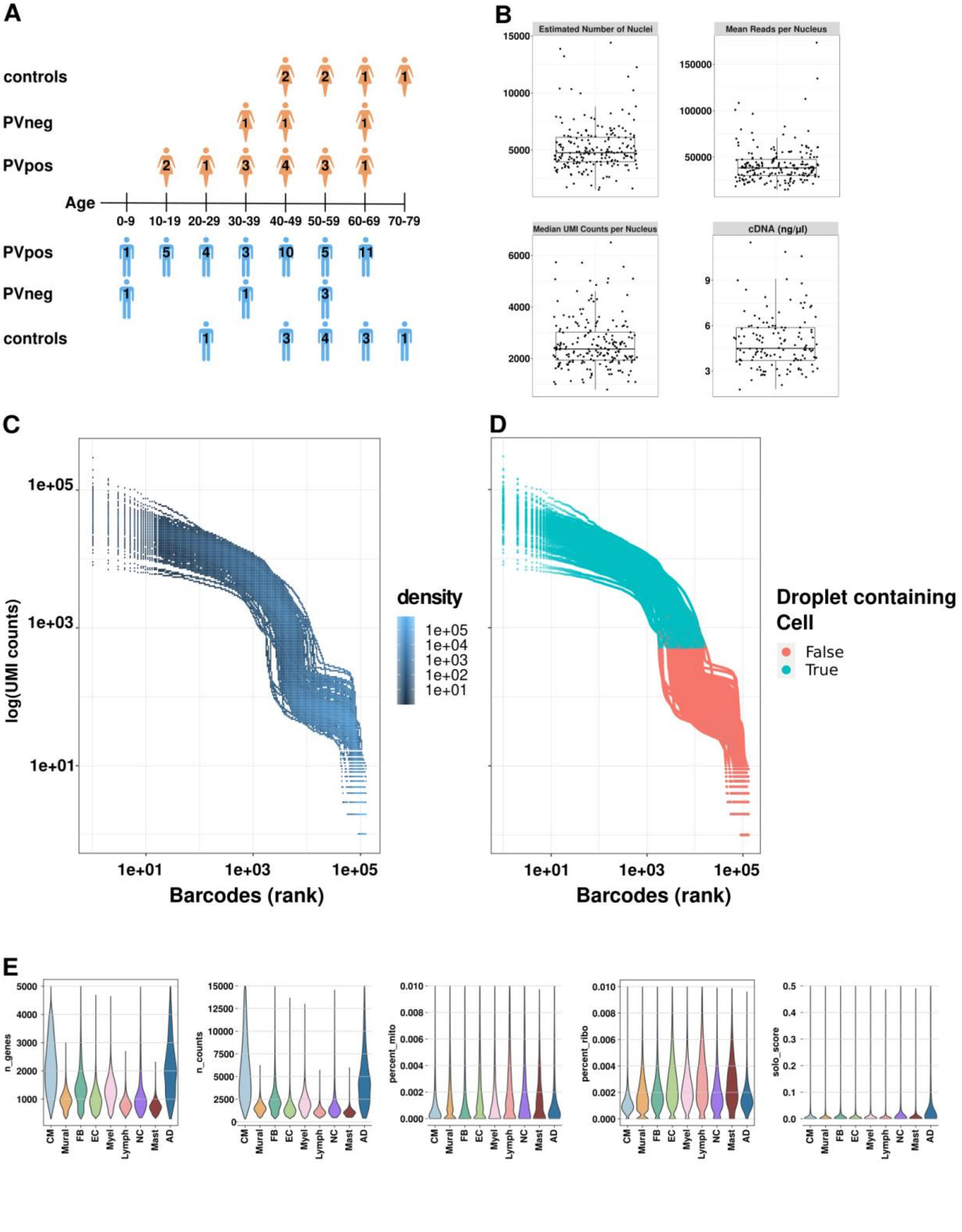

A
controls
PVneg
PVpos
Age
0-9
10-19
20-29
30-39
40-49
50-59
60-69
70-79
PVpos
PVneg
controls
B
Estimated Number of Nuclei
Mean Reads per Nucleus
Median UMI Counts per Nucleus
cDNA (ng/µl)
C
log(UMI counts)
Barcodes (rank)
density
D
Barcodes (rank)
Droplet containing Cell
False
True
E
n_genes
n_counts
percent_mito
percent_ribo
solo_score
CM
Mural
FB
EC
Myel
Lymph
NC
Mast
AD

Figure S1: Quality assessment of snRNAseq data

(A) Infographic depicts the 78 individuals studied (women, pink; men, blue; binned by age range), including controls (unused donor hearts), patients with pathogenic variants (PV pos) or patients with unknown disease etiology (PVneg). Further clinical details are provided in the metadata sheet (Table S1). **(B)** Estimated number of nuclei, mean reads and median UMI count per nuclei (Cellranger) as well as cDNA (ng/µl). **(C)** Barcode rank plots across all samples. A clear distinction between nuclei containing droplets and empty droplets (background ambient RNA) indicated a low overall background. **(D)** Barcode rank plot across all samples as in (C). Coloring code shows classification of nuclei: turquoise, nuclei-containing droplets (true); red, empty droplet, background, or ambient RNA (false). **(E)** Violin plots show number of genes (n_genes), number of UMIs (n_counts), percent UMIs mapping to mitochondrial (percent_mito) and ribosomal genes (percent_ribo) and doublet score (solo_score) plotted per cell type after quality control filtering and clustering.

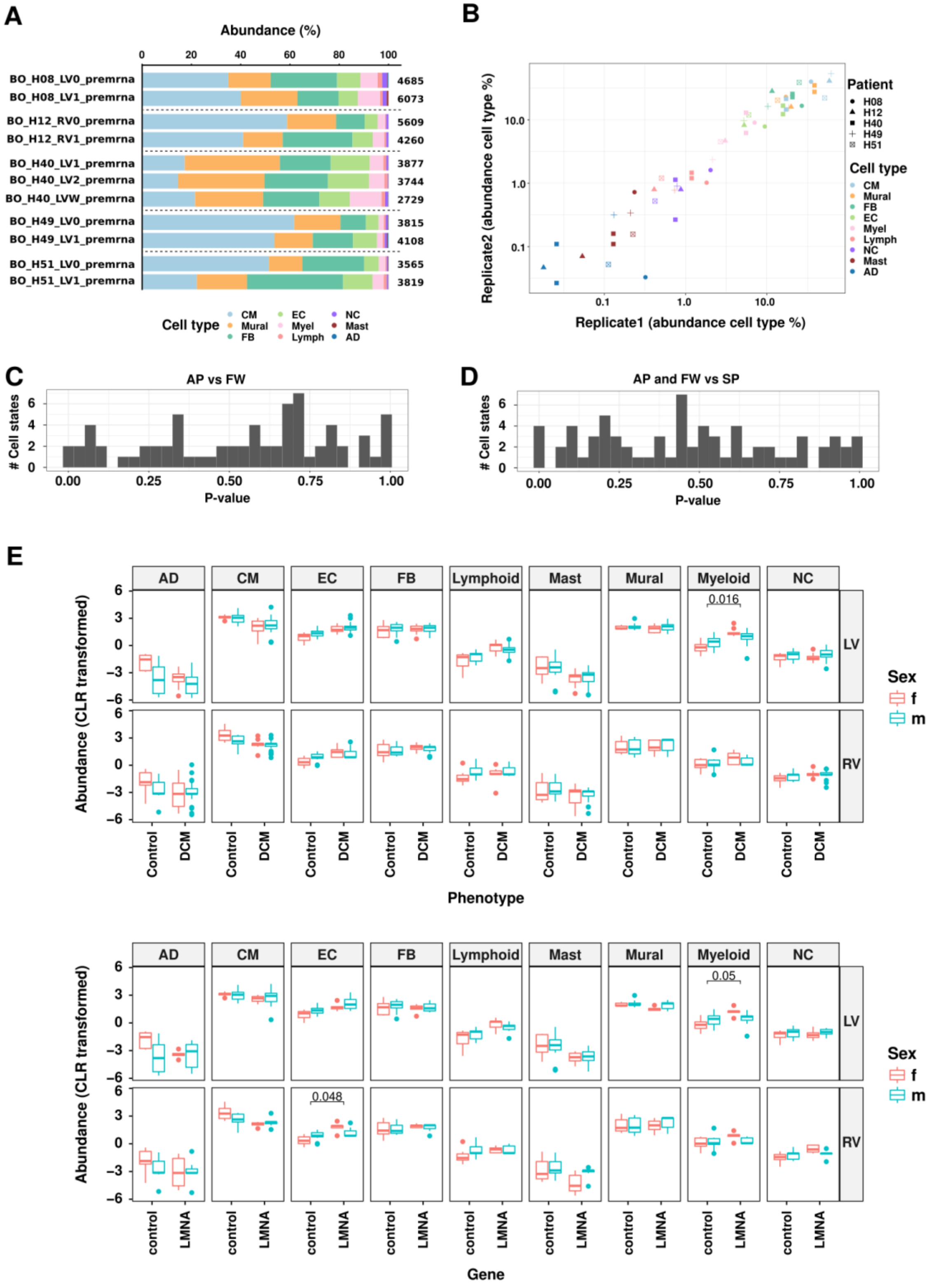

A
Abundance (%)
0
20
40
60
80
100
BO_H08_LV0_premrna
BO_H08_LV1_premrna
BO_H12_RV0_premrna
BO_H12_RV1_premrna
BO_H40_LV1_premrna
BO_H40_LV2_premrna
BO_H40_LVW_premrna
BO_H49_LV0_premrna
BO_H49_LV1_premrna
BO_H51_LV0_premrna
BO_H51_LV1_premrna
4685
6073
5609
4260
3877
3744
2729
3815
4108
3565
3819
Cell type
CM
Mural
FB
EC
Myel
Lymph
NC
Mast
AD
B
Replicate2 (abundance cell type %)
Replicate1 (abundance cell type %)
0.1
1.0
10.0
Patient
H08
H12
H40
H49
H51
Cell type
CM
Mural
FB
EC
Myel
Lymph
NC
Mast
AD
C
AP vs FW
Cell states
P-value
0
2
4
6
0.00
0.25
0.50
0.75
1.00
D
AP and FW vs SP
Cell states
P-value
0
2
4
6
0.00
0.25
0.50
0.75
1.00
E
AD
CM
EC
FB
Lymphoid
Mast
Mural
Myeloid
NC
Abundance (CLR transformed)
6
3
0
−3
−6
0.016
LV
RV
Control
DCM
Phenotype
Sex
f
m
AD
CM
EC
FB
Lymphoid
Mast
Mural
Myeloid
NC
Abundance (CLR transformed)
6
3
0
−3
−6
0.05
0.048
LV
RV
control
LMNA
Gene
Sex
f
m

Figure S2: Schematic of study cohort, replicate analyses, and comparison of LV regions

(A) Biological replicates of tissue samples obtained from neighboring areas of the same heart (from individuals H08, H12, H40, H49 and H51) and studied after libraries were generated in different batches. **(B)** There was a high correlation between replicates (0.74 and 0.99), based on cell type proportions. **(C)** Histogram showing *p*-value distribution of differential cell state abundances between LV free walls (FW) and apex (AP) samples. *p*-values were uniformly distributed, and no significant differences were detected at FDR < 10%. **(D)** Parallel comparisons as shown in (D), between LV apical (AP) and free wall (FW) samples versus septum (SP). Based on these analyses, data was combined for all LV regions**. (E)** Abundance changes (CLR transformed) of cell types in healthy control compared to all DCM (*LMNA*, *TTN*, *RBM20*, PVneg) and *LMNA* only.

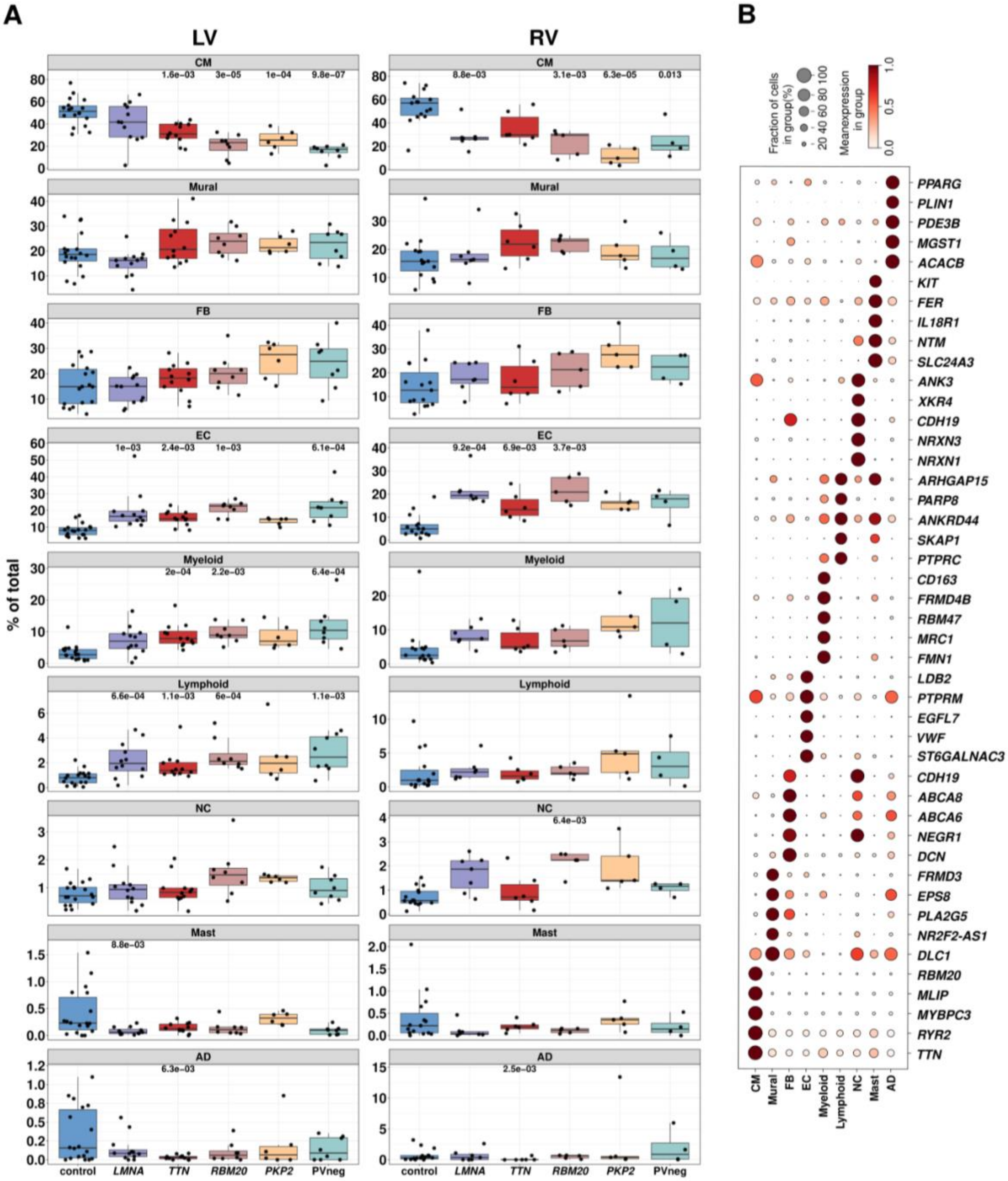

Figure S3: Abundance analyses and marker genes of cardiac cell types in LVs and RVs

(A) Box plots show cell type distribution across controls and genotypes in LVs and RVs. *p*-values are indicated for significant proportional changes, FDR<0.05. **(B)** Dotplots show selected marker genes of each cell type. Dot size, fraction (%) of expressing cells; color, mean expression level.

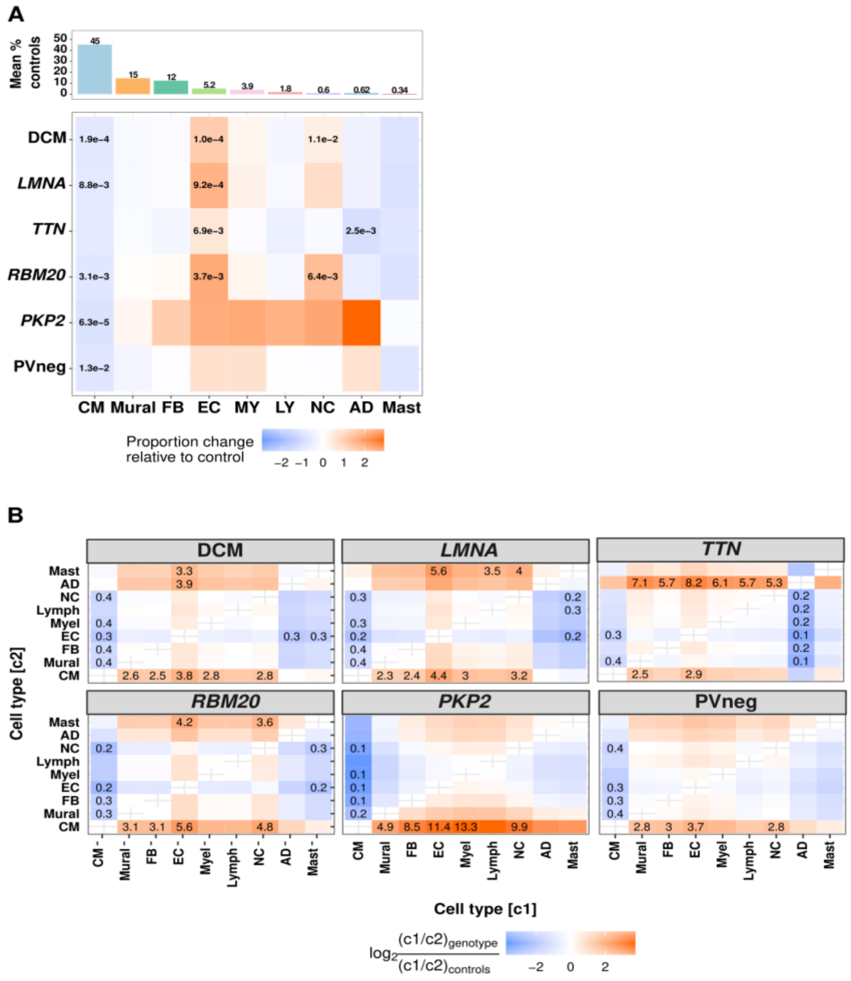

Figure S4: Relative cellular composition analyses in RV

(A) Upper panel: Mean abundance (%) of cell types in healthy control RVs. Lower panel: Proportional changes of cell types in specified genotypes or aggregated across DCM genotypes. Proportional changes are scaled by color: increased (red) or decreased (blue) in disease versus control. *p*-values are indicated for significant proportional changes, FDR<0.05. **(B)** Pairwise cell-type abundance ratios in specified genotypes or aggregated DCM genotypes in RVs relative to controls. Color scale, FDR, significance depicted as in (A).

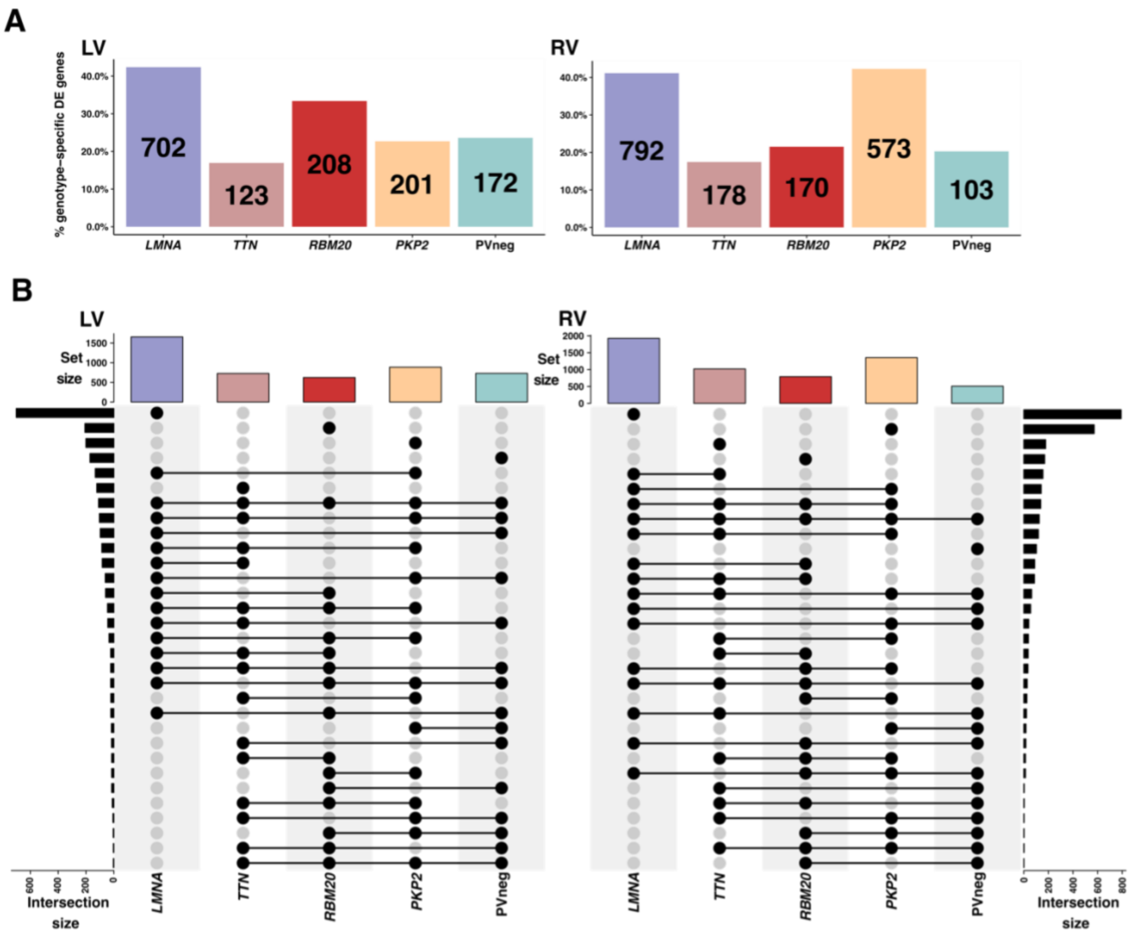

Figure S5: Genotype specific upregulated genes in cardiomyocytes (CMs)

(A) Total number of uniquely upregulated genes (log2FC >0.5) for each genotype in LVs and RVs, across all CM states. FDR<0.05. **(B)** Upset plots of all upregulated genes (log2FC>0.5, FDR<0.05) in LVs and RVs demonstrated shared (connected by lines) and specific expression (no connected lines) by genotypes. The total number of genes in the set is plotted as a bar on top (set size).

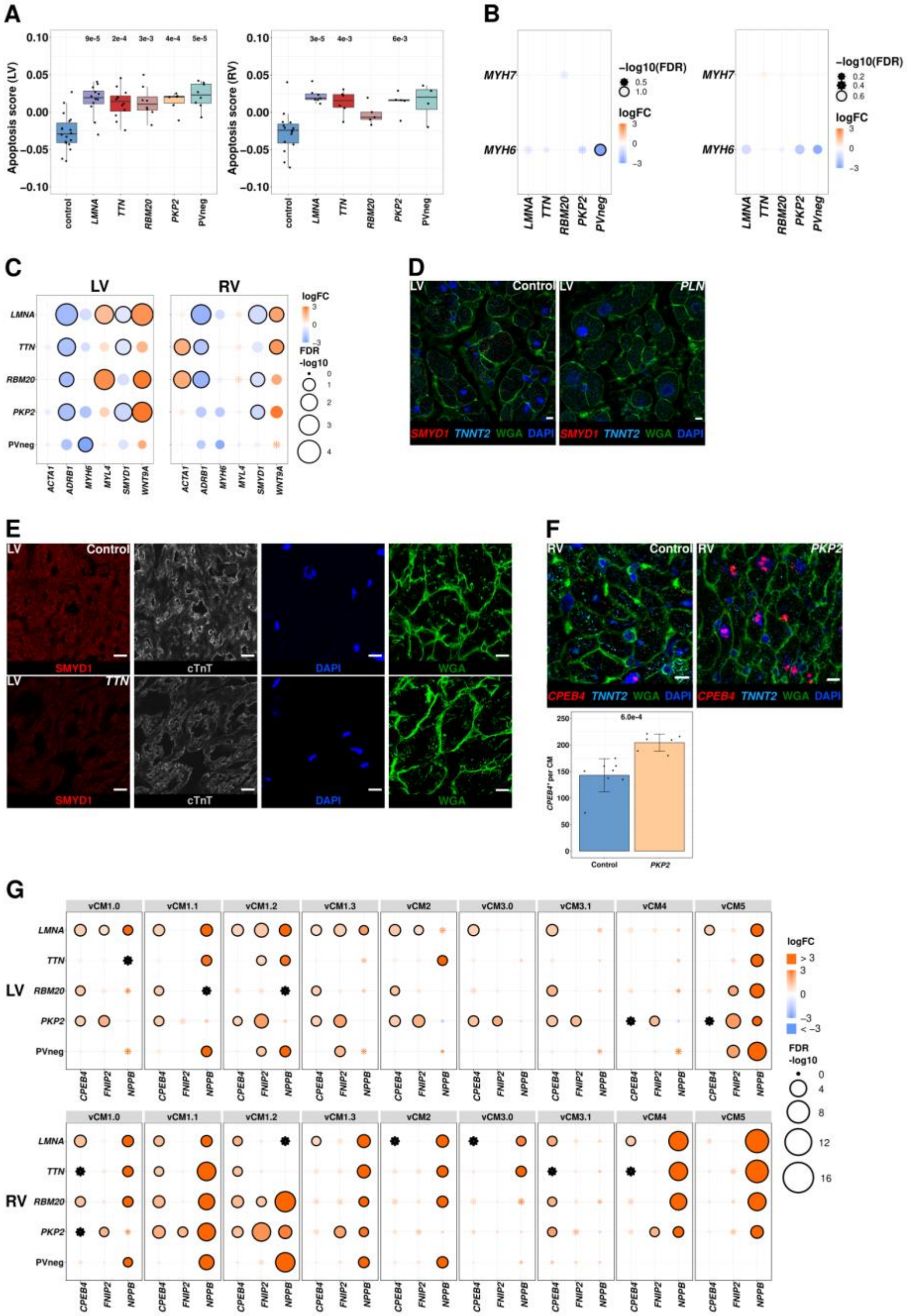

Figure S6: Genotype specific compositional changes across cardiomyocyte (CM) cell states

(A) Box plots show apoptosis gene expression scores in LV and RV CMs. Significant *p*-values comparing the genotype to control (FDR<0.05) are shown above the whiskers. **(B)** Dotplots illustrate fold-change (log2FC) and significance (-lg(FDR)) of *MHY6* and *MYH7* across diseased genotypes and all cell states in LVs (left) and RVs (right) **(C)** Dotplots illustrate fold-change (log2FC) and significance (-lg(FDR)) of selected dysregulated genes across diseased genotypes and all cell states in the LVs and RVs. **(D)** Single-molecule RNA fluorescent *in situ* hybridization exemplifies decreased *SMYD1* (red) expression in CMs (identified by *TNNT2* transcripts, cyan) within a DCM heart

with a PV in *PLN* (phospholamban). **(E)** Single-channel images of SMYD1 immunohistochemistry are shown in Figure 2D. **(F)** (Left) Single-molecule RNA-fluorescent *in situ* hybridization (RNA-scope) of *CPEB4* (red) and *TNNT2* (cyan) in control and *PKP2* RVs. (Right) Bar graph showing quantified expression of *CPEB4* assessed in controls vs. *PKP2* (H-score, spots per CM) (right) with *p*-value indicated. Cell boundaries, WGA-stained (green); nuclei, DAPI-stained (blue); bar 10 µm. **(G)** Doptplots visualize the levels of fold-change (logFC) and significance (-log10(FDR)) of selected dysregulated genes across diseased genotypes and each cell state in LVs and RVs.

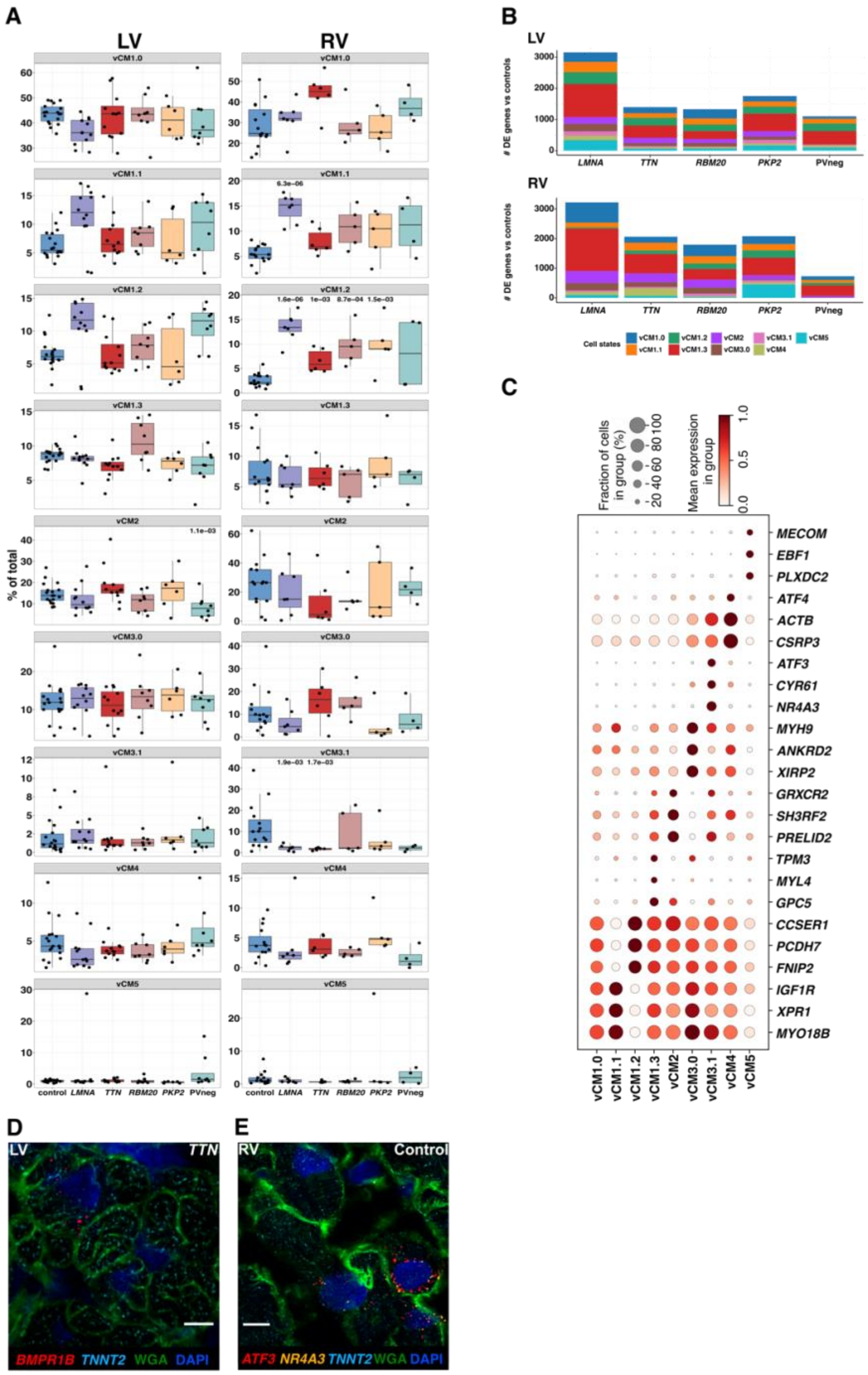

Figure S7: Characterization of cardiomyocyte (CM) state abundance and gene expression

(A) Box plots show CM state distribution across controls and genotypes in LVs and RVs. *p*-values are indicated for significant proportional changes, FDR<0.05. **(B)** Total number of upregulated genes across CM states and genotypes in LVs and RVs. Only significantly upregulated expressed genes (log2FC >0.5) are shown, FDR<0.05. **(C)** Dotplot shows selected marker genes of CM states. Dot size, fraction (%) of expressing cells; color, mean expression level. **(D)** Single-molecule RNA-fluorescent *in situ* hybridization (RNA-scope) of *BMPR1B* (red) enriched in vCM1.3 and

TNNT2 (cyan) in *TTN* LV. **(E)** RNA-scope of *ATF3* (red) and *NR4A3* (yellow), both enriched in vCM3.1, as well as *TNNT2* (cyan) in control LV and RV. **(D and E)** Cell boundaries, WGA-stained (green); nuclei, DAPI-stained blue); bar 10 µm (note longer bar in *TTN* panel).

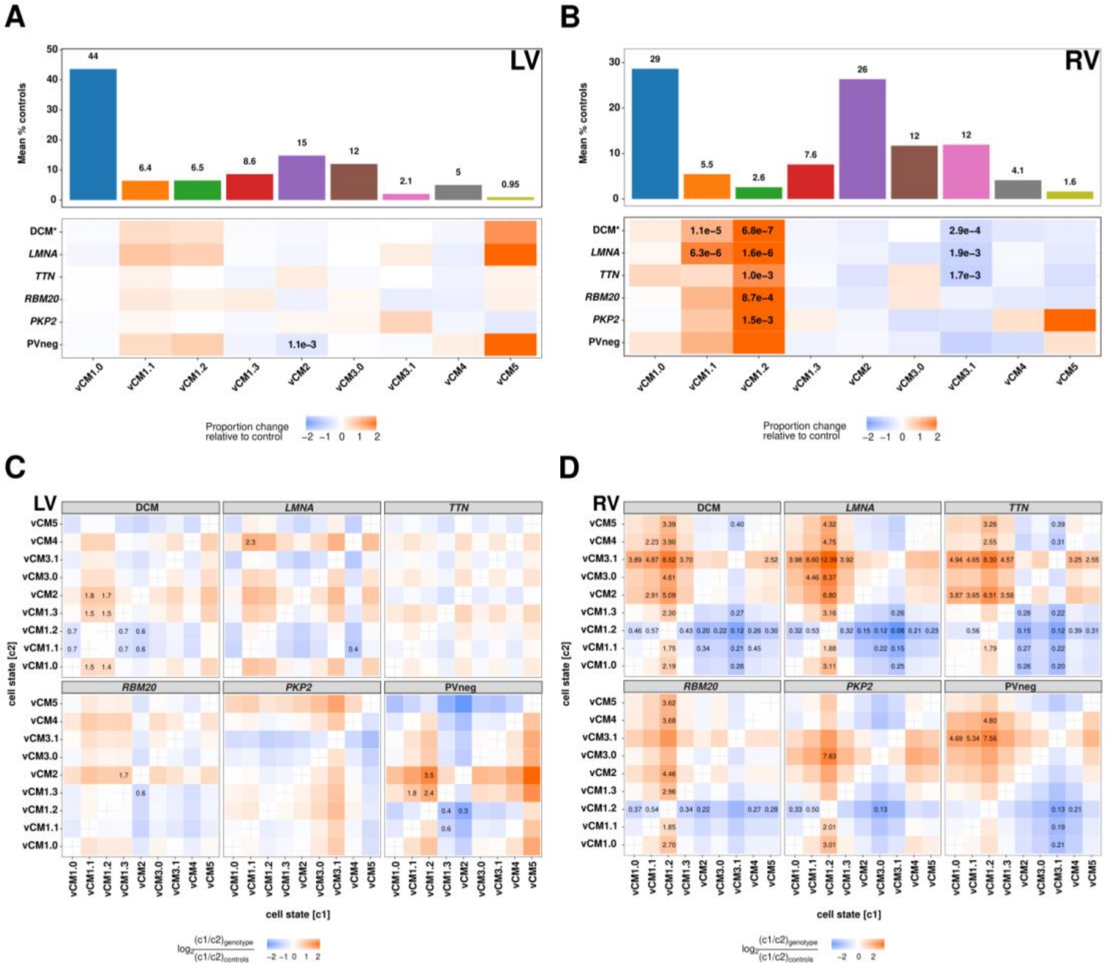

Figure S8: Genotype specific compositional changes in cardiomyocytes (CMs)

(**A**) Upper panel: Mean abundance (%) of CM states in healthy control LVs. Lower panel: Proportional changes of CM states in specified genotypes or aggregated across DCM genotypes. (**B**) as in (A) but for RVs. (**C**) Pairwise CM state abundance ratios in specified genotypes or aggregated DCM genotypes in LVs relative to controls. (**D**) as in (C) but for RVs.

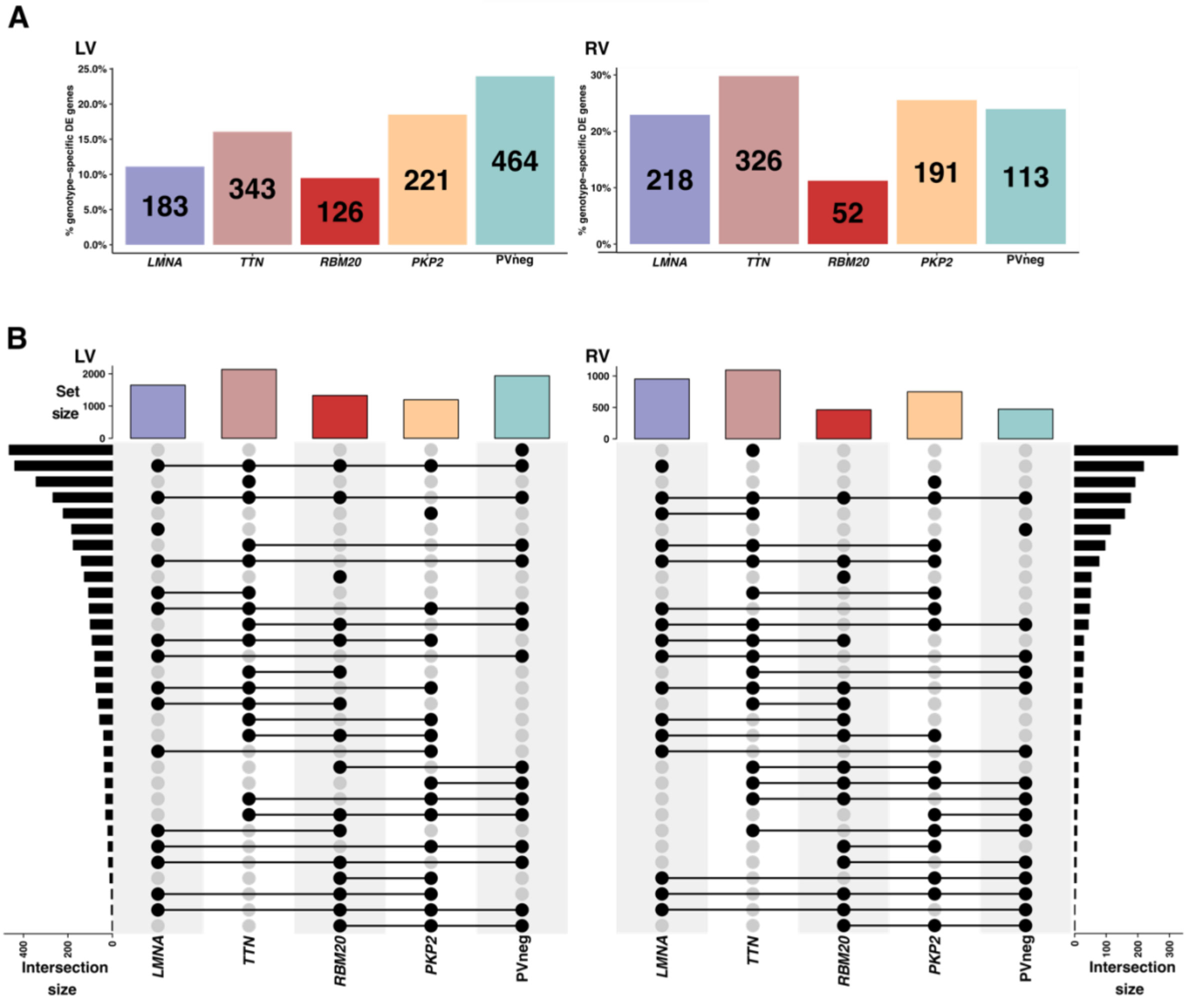

Figure S9: Genotype specific upregulated genes in fibroblasts (FBs)

(A) Total number of uniquely upregulated genes (log2FC >0.5) for each genotype in LVs and RVs across all FB states, FDR<0.05. **(B)** Upset plots of all upregulated genes (log2FC>0.5, FDR<0.05) in LVs and RVs demonstrated shared (connected by lines) and specific expression (no connected lines) by genotypes. The total number of genes in the set is plotted as a bar on top (set size).

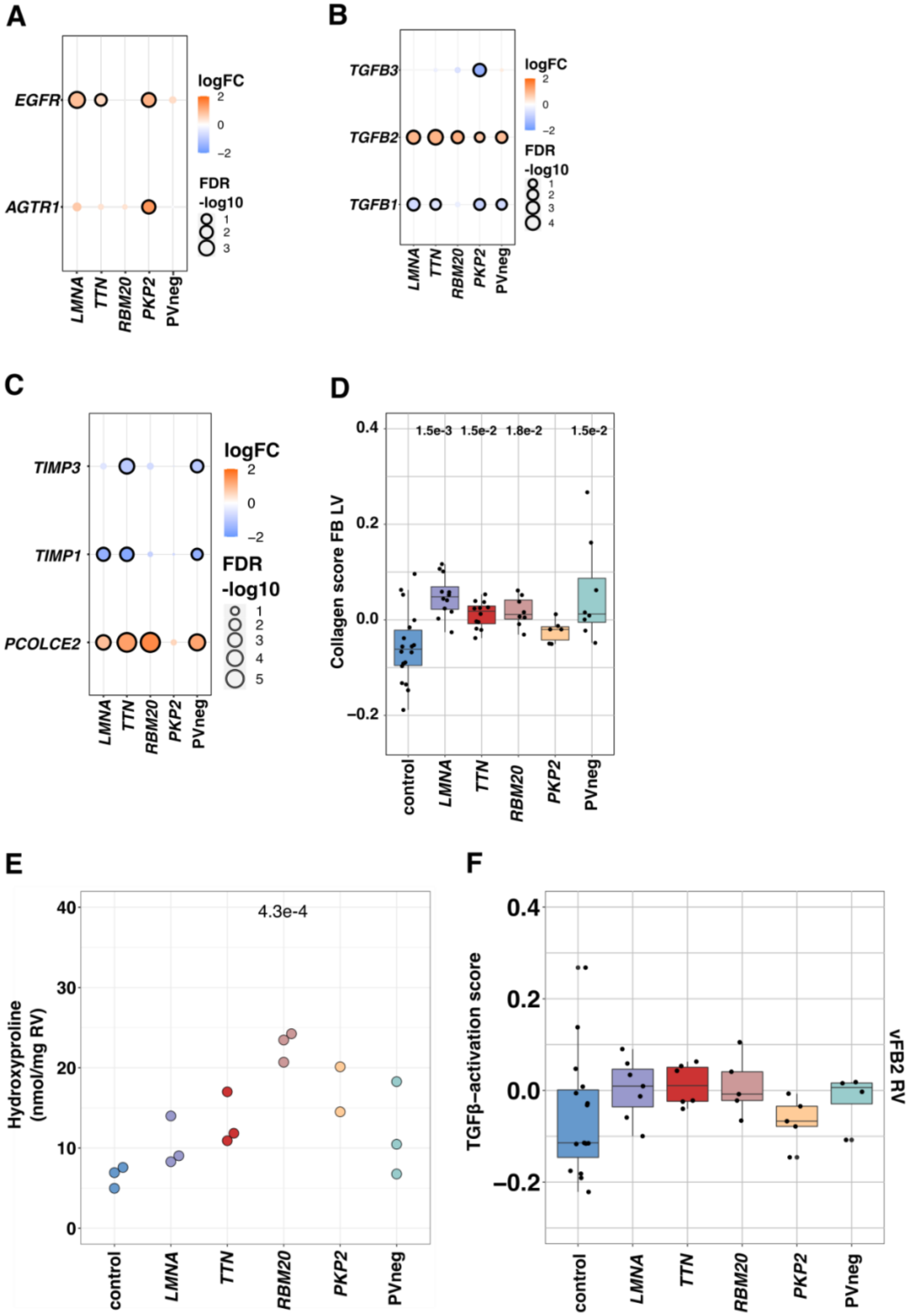
A
EGFR
AGTR1
LMNA
TTN
RBM20
PKP2
PVneg
logFC
FDR
-log10
B
TGFB3
TGFB2
TGFB1
C
TIMP3
TIMP1
PCOLCE2
D
Collagen score FB LV
1.5e-3
1.5e-2
1.8e-2
1.5e-2
control
E
Hydroxyproline
(nmol/mg RV)
4.3e-4
F
TGFβ–activation score
vFB2 RV

Figure S10: Genotype specific gene expression in fibroblasts (FBs)
(A) Dot plot shows levels of *EGFR* and *AGTR1* across genotypes in LV FBs compared to control. **(B)** Dot plot shows levels of fold-change (logFC) and significance (-log10(FDR)) of *TGFB1-3* across genotypes in LV FBs compared to control. **(C)** Dot plot illustrates fold-change (log2FC) and significance (-lg(FDR)) of selected genes encoding ECM modulators in FBs across genotypes. **(D)** Box plot shows collagen gene expression scores in LV FBs. Significant *p*-values comparing the genotype to control (FDR<0.05) are shown above the whiskers. **(E)** Hydroxyproline assay (HPA) in RVs quantifying cardiac collagen content for each genotype. *p*-values indicate significant differences. **(F)** TGFβ activation score for vFB2 in RV. (A, B and D) Dot sizes represent significance values; color intensity denotes fold-change.

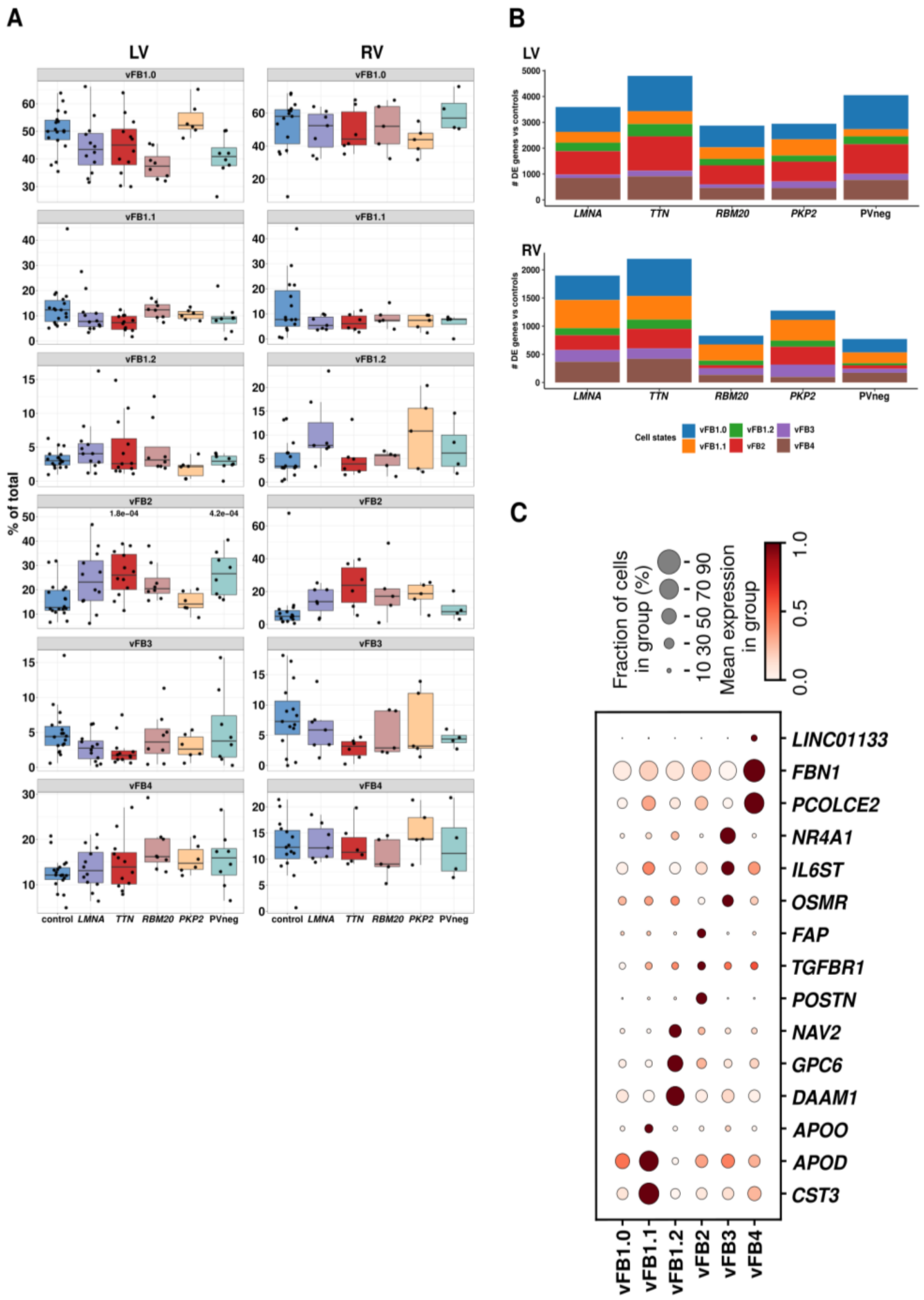

Figure S11: Characterization of fibroblast (FB) state abundance and gene expression

(A) Box plots show FB state distribution across controls and genotypes in LVs and RVs. *p*-values are indicated for significant proportional changes, FDR<0.05. **(B)** Total number of upregulated genes across FB states and genotypes in LVs and RVs. Only significantly upregulated expressed genes (log2FC >0.5) are shown, FDR<0.05. **(C)** Dotplot shows selected marker genes of fibroblast states. Dot size, fraction (%) of expressing cells; color, mean expression level.

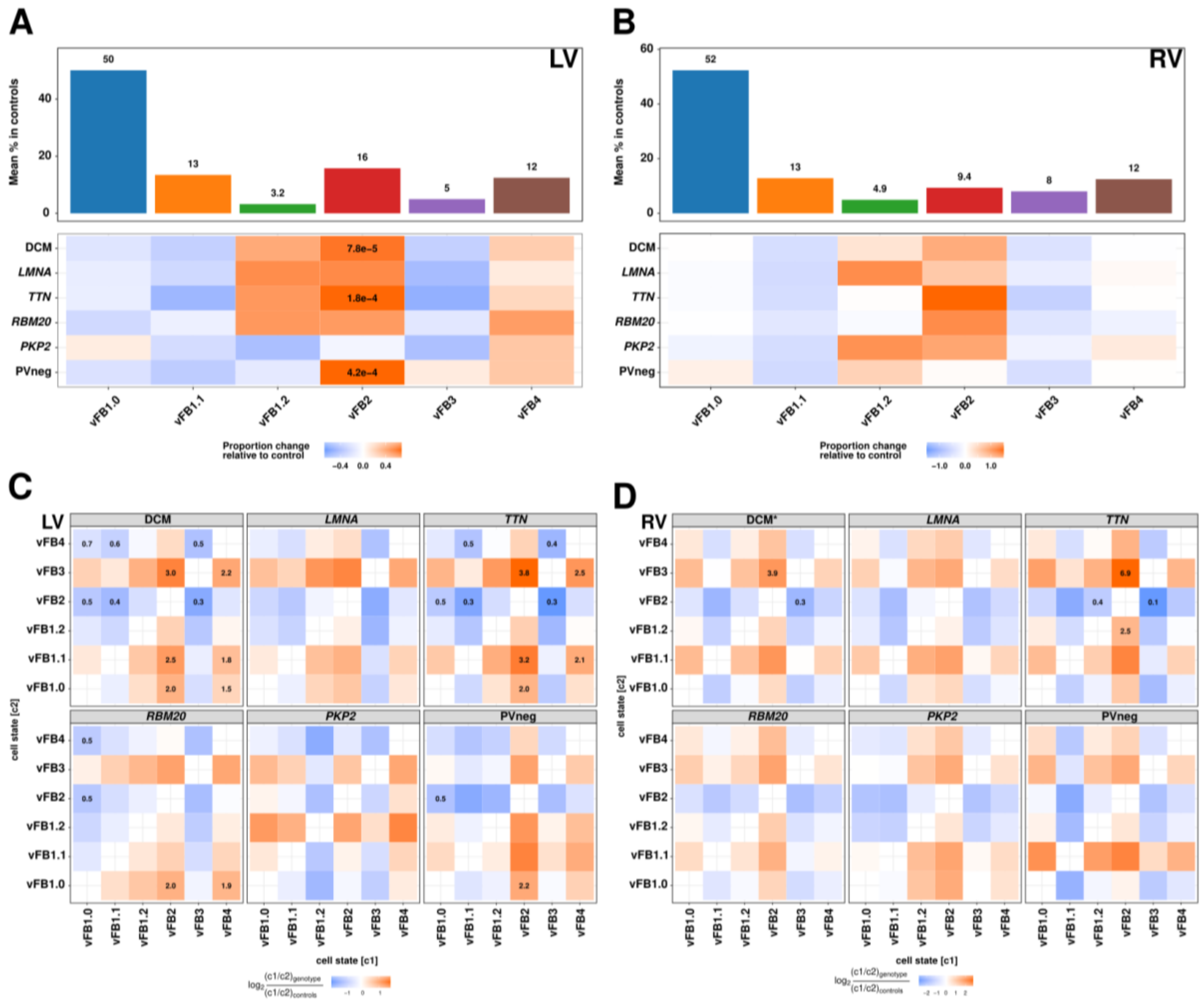

Figure S12: Genotype specific compositional changes in fibroblasts (FBs)

(A) Upper panel: Mean abundance (%) of FB states in control LVs. Lower panel: Proportional changes of FB states in specified genotypes or aggregated across DCM genotypes. **(B)** as in (A) but for RVs. **(C)** Pairwise FB state abundance ratios in specified genotypes or aggregated DCM genotypes in LVs relative to controls. (**D**) as in (C) but for RVs.

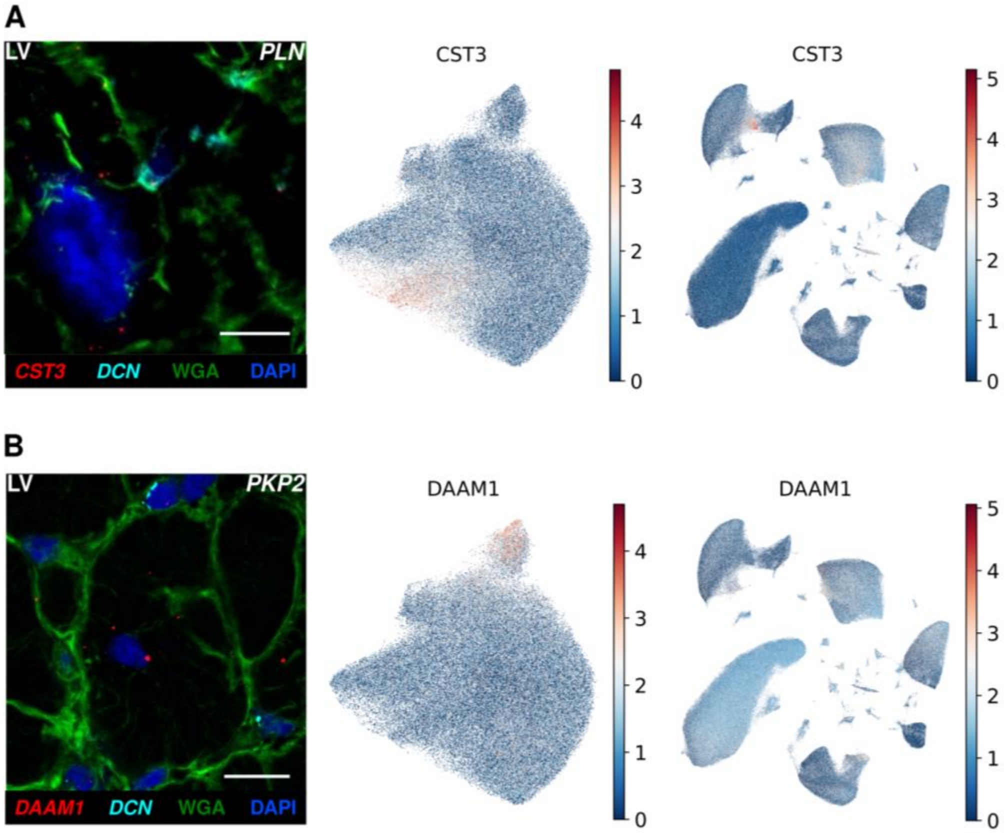

Figure S13: Validation of fibroblast (FB) states vFB.1 and 1.2

(A) Single-molecule RNA-fluorescent *in situ* hybridization (RNAscope) demonstrated colocalization of *CST3* (red) and *DCN* (cyan), enriched in vFB1.1 (exemplified in *PLN* LV). UMAP representations depict FB state (mid) and cell type (right) specificity of *CST3*. **(B)** RNAscope demonstrated colocalization of *DAAM1* (red) and *DCN* (cyan), enriched in vFB1.2 (exemplified in *PKP2* LV). DCN serves as a pan-fibroblast marker cell boundaries, WGA-stained (green); nuclei, DAPI-stained (blue); bar 10 µm. UMAP representations depict FB state (mid) and cell type (right) specificity of *DAAM1*.

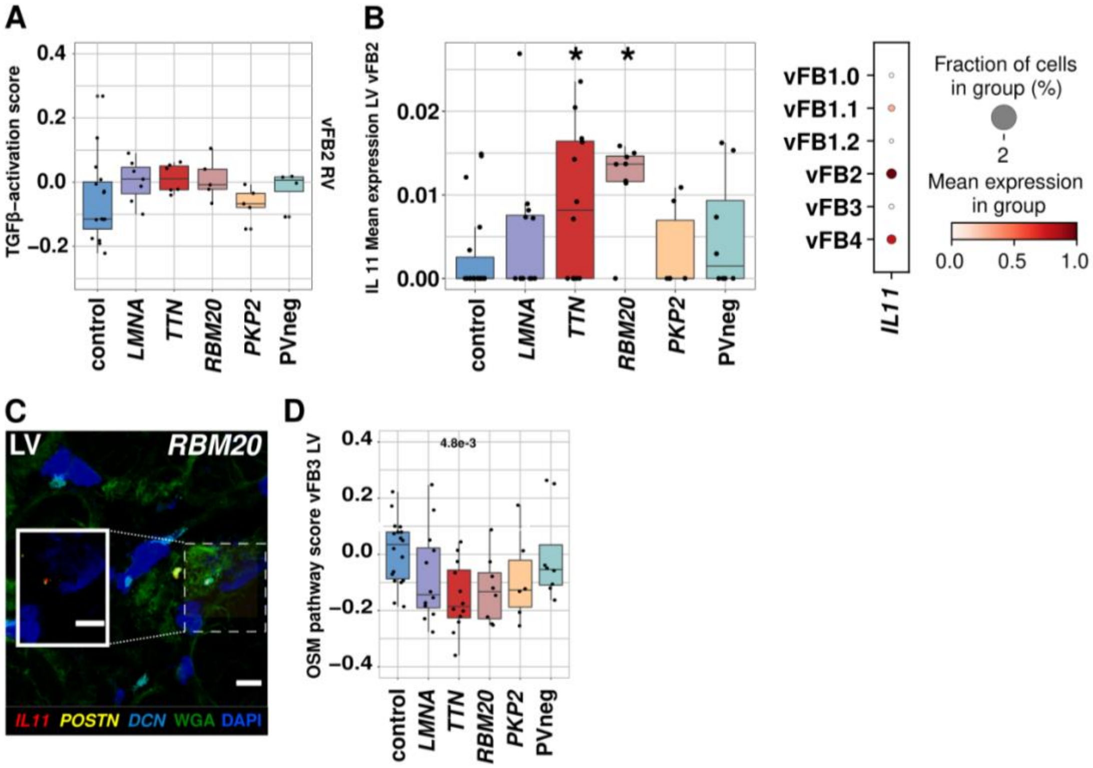

Figure S14: Analyses of gene expression indicating fibroblast (FB) activation

(A) TGFβ-activation score in RV vFB2. Significant *p*-values comparing genotypes to control are shown above the whiskers. **(B)** Left: Box plot visualizes the level of average *IL11* expression in LV vFB2 across genotypes. *p*-values were calculated using a hypergeometric test. *p*-values <0.05 are indicated with *. Right: Dot plot showing *IL11* expression across all fibroblast states. The dot sizes represent the fraction (%) of expressing cells; the color scale represents the corresponding scaled mean expression levels. **(C)** Single-molecule RNA-fluorescent *in situ* hybridization (RNAscope) of *IL11* (red), *POSTN* and *DCN* (cyan) expression (yellow) in *LMNA* LV (left) and *RBM20* LV (right). DCN serves as a pan-fibroblast marker while *POSTN* expression depicts activated fibroblast (vFB2). Cell boundaries, WGA-stained (green); nuclei, DAPI-stained (blue); bar 10 µm. **(D)** OSM pathway score in LV vFB3. Significant *p*-values comparing genotypes to control are shown above the whiskers.

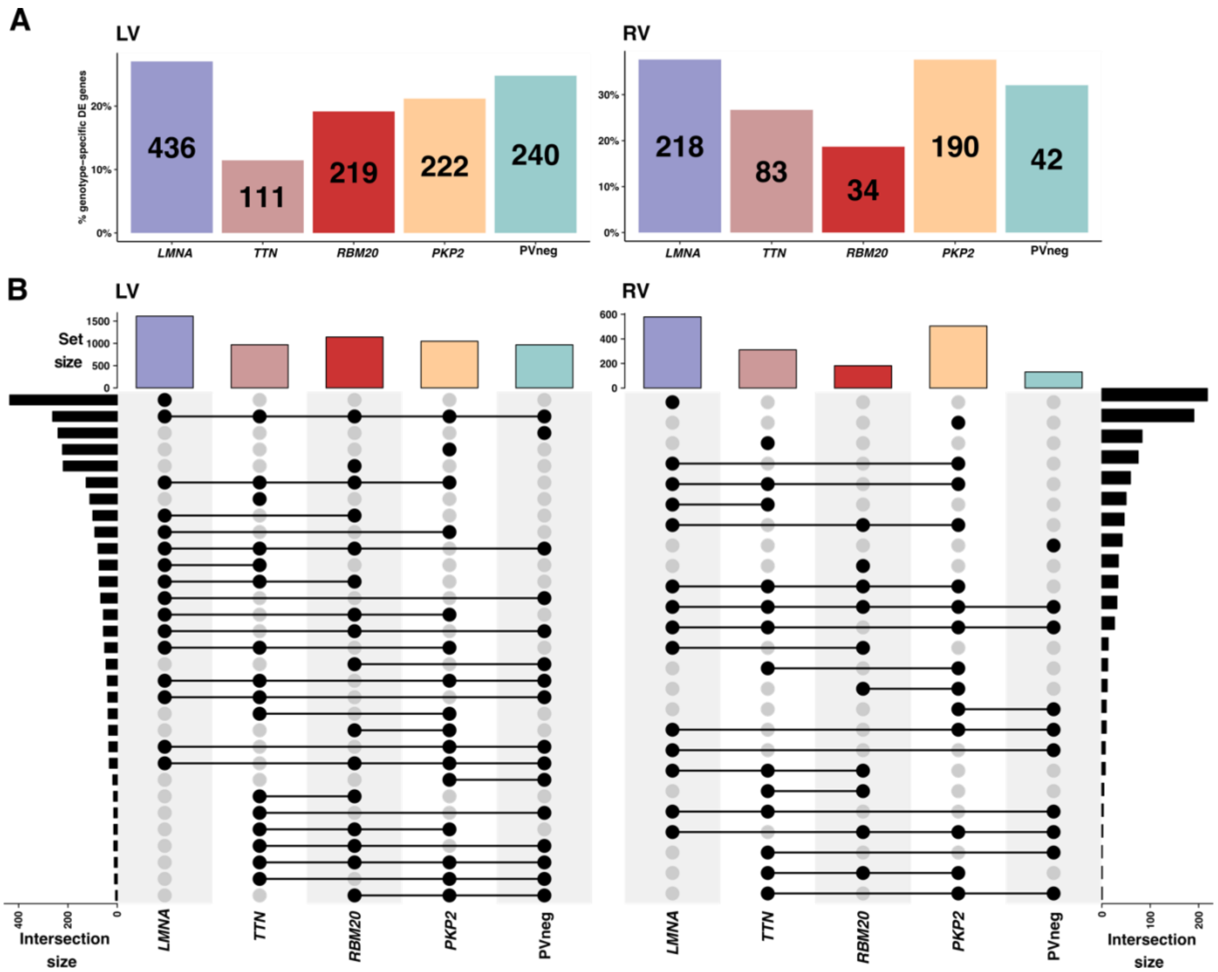

Figure S15: Genotype specific upregulated genes in mural cells (MCs)

(A) Total number of uniquely upregulated genes (log2FC>0.5) for each genotype in LVs and RVs across MC states, FDR<0.05. **(B)** Upset plots of all upregulated genes (log2FC>0.5, FDR<0.05) in LVs and RVs demonstrated shared (connected by lines) and specific expression (no connected lines) by genotypes. The total number of genes in the set is plotted as a bar on top (set size).

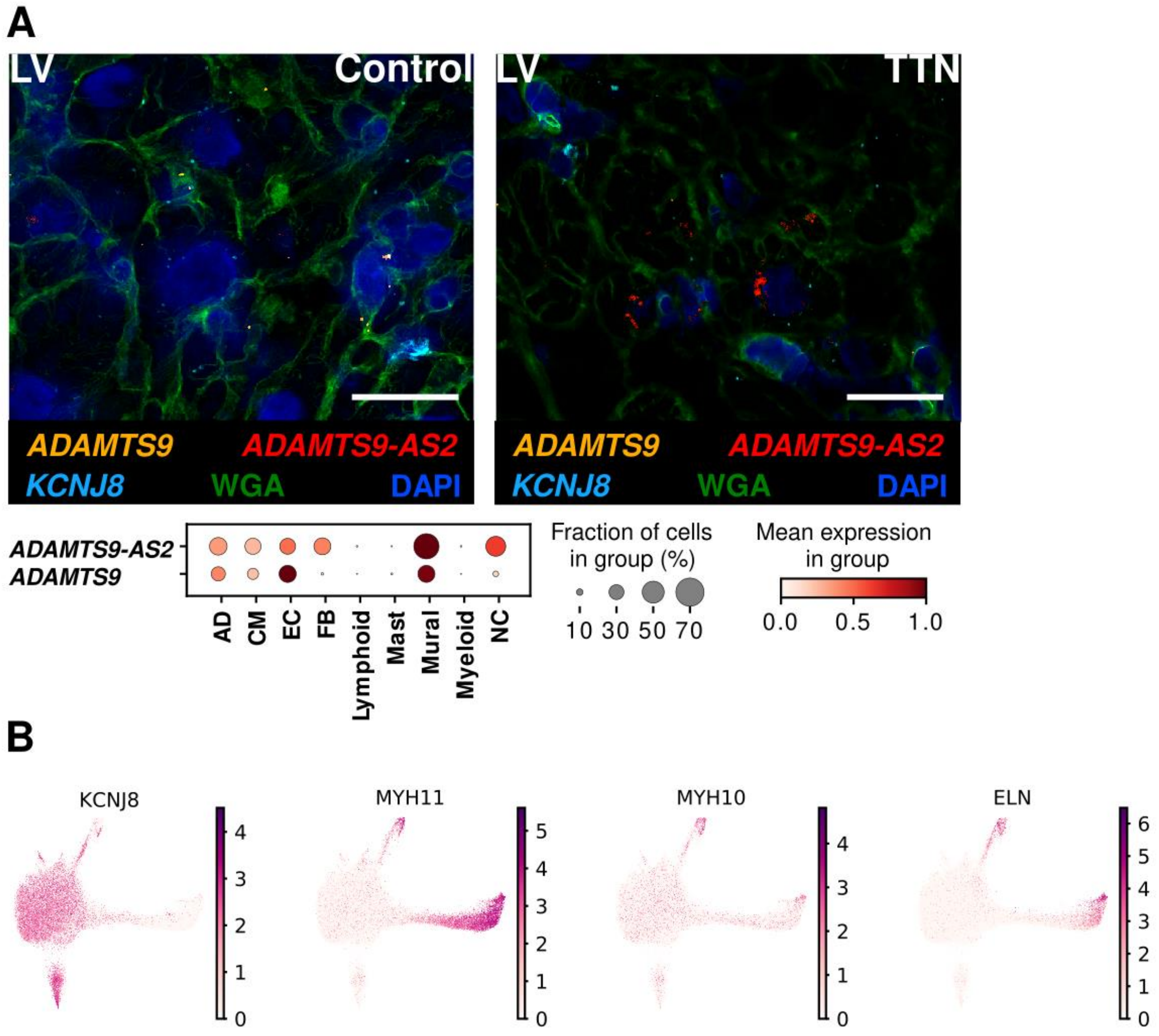

Figure S16: Validation of mural cell (MC) states

(**A**) Validation of increased *ADAMTS-AS2* and decreased *ADAMTS9* expression in MCs of diseased genotypes using RNA *in situ* hybridization (RNAscope). RNAscope of *ADAMTS9* (orange) and *ADAMTS-AS2* (red) in control and *TTN* LVs. *KCNJ2* (cyan) served as a marker for mural cells. Cell boundaries: WGA (green), nuclei stain: DAPI (dark blue), bar length: 10 µm. Dotplot shows *ADAMTS9-AS2* and *ADAMTS9* expression across cell types. Dot size, fraction (%) of expressing cells; color, mean expression level. (**B**) Feature plot shows selected marker genes of MC states.

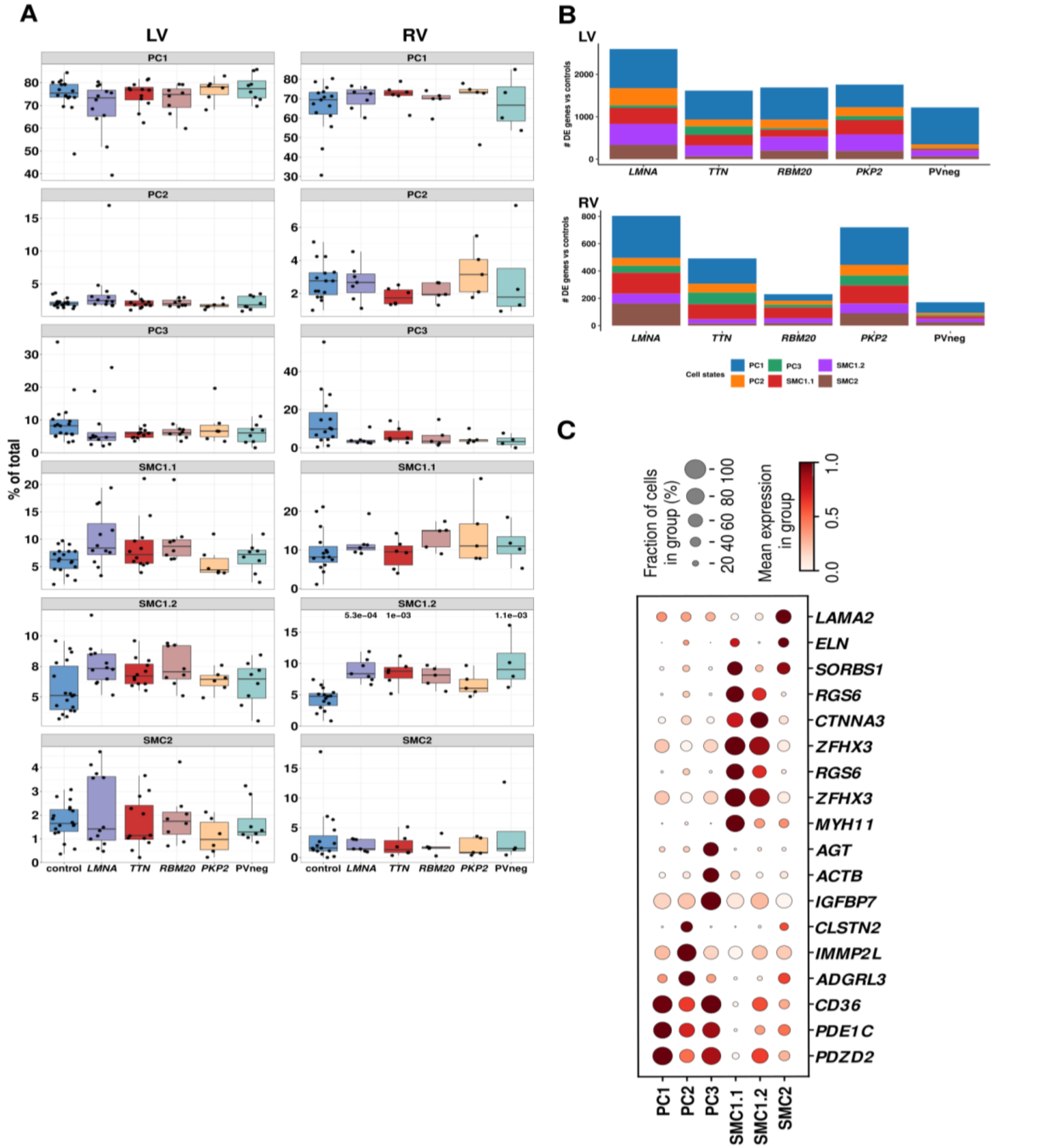

Figure S17: Characterization of MC state abundance and gene expression

(A) Box plots show MC state distribution across controls and genotypes in LVs and RVs. *p*-values are indicated for significant proportional changes, FDR<0.05. **(B)** Total number of upregulated genes across MC states and genotypes in LVs and RVs. Only significantly upregulated expressed genes (log2FC >0.5) are shown, FDR<0.05. **(C)** Dotplot shows selected marker genes of MC states. Dot size, fraction (%) of expressing cells; color, mean expression level.

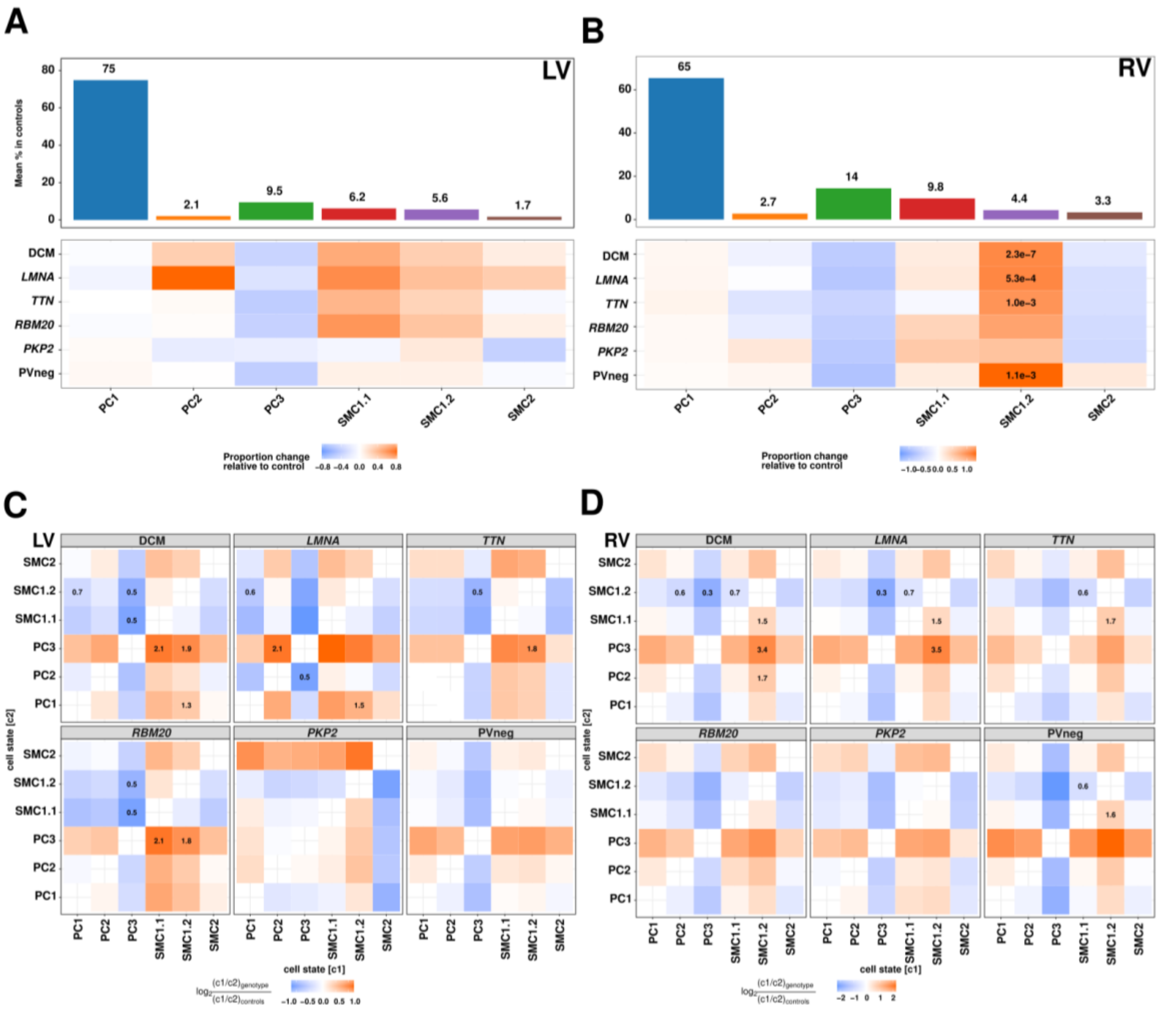

Figure S18: Genotype specific compositional changes in mural cells (MCs)

(**A**) Upper panel: Mean abundance (%) of MC states in control LVs. Lower panel: Proportional changes of MC states in specified genotypes or aggregated across DCM genotypes. (**B**) as in A but for RVs. (**C**) Pairwise MC state abundance ratios in specified genotypes or aggregated DCM genotypes in LVs relative to controls. (**D**) as in (C) but for RVs.

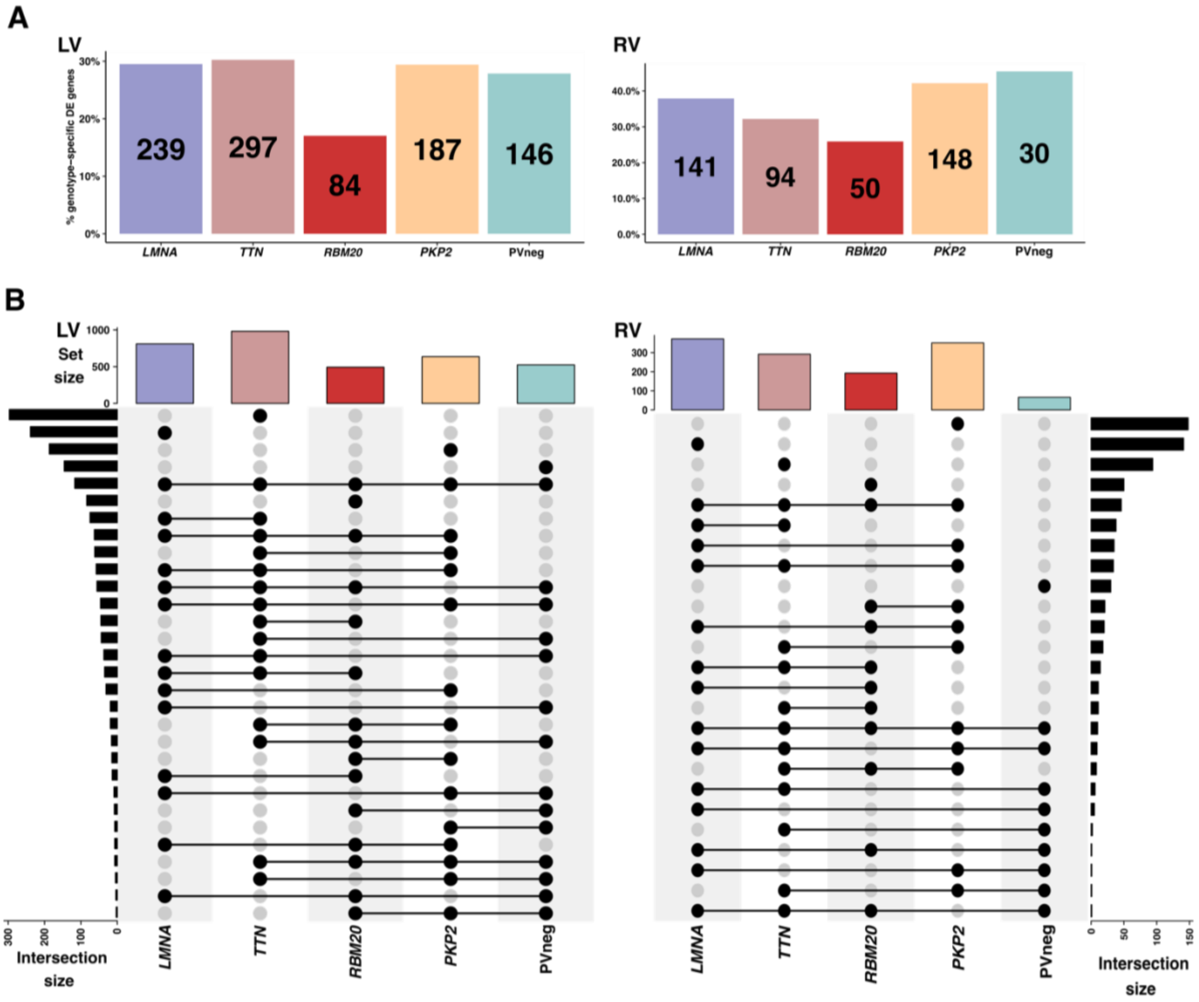

Figure S19: Genotype specific upregulated genes in endothelial cells (ECs)

(**A**) Total number of uniquely upregulated genes (log2FC>0.5) for each genotype in LVs and RVs across all EC states, FDR<0.05. (**B**) Upset plots of all upregulated genes (log2FC>0.5, FDR<0.05) in LVs and RVs demonstrated shared (connected by lines) and specific expression (no connected lines) by genotypes. The total number of genes in the set is plotted as a bar on top (set size).

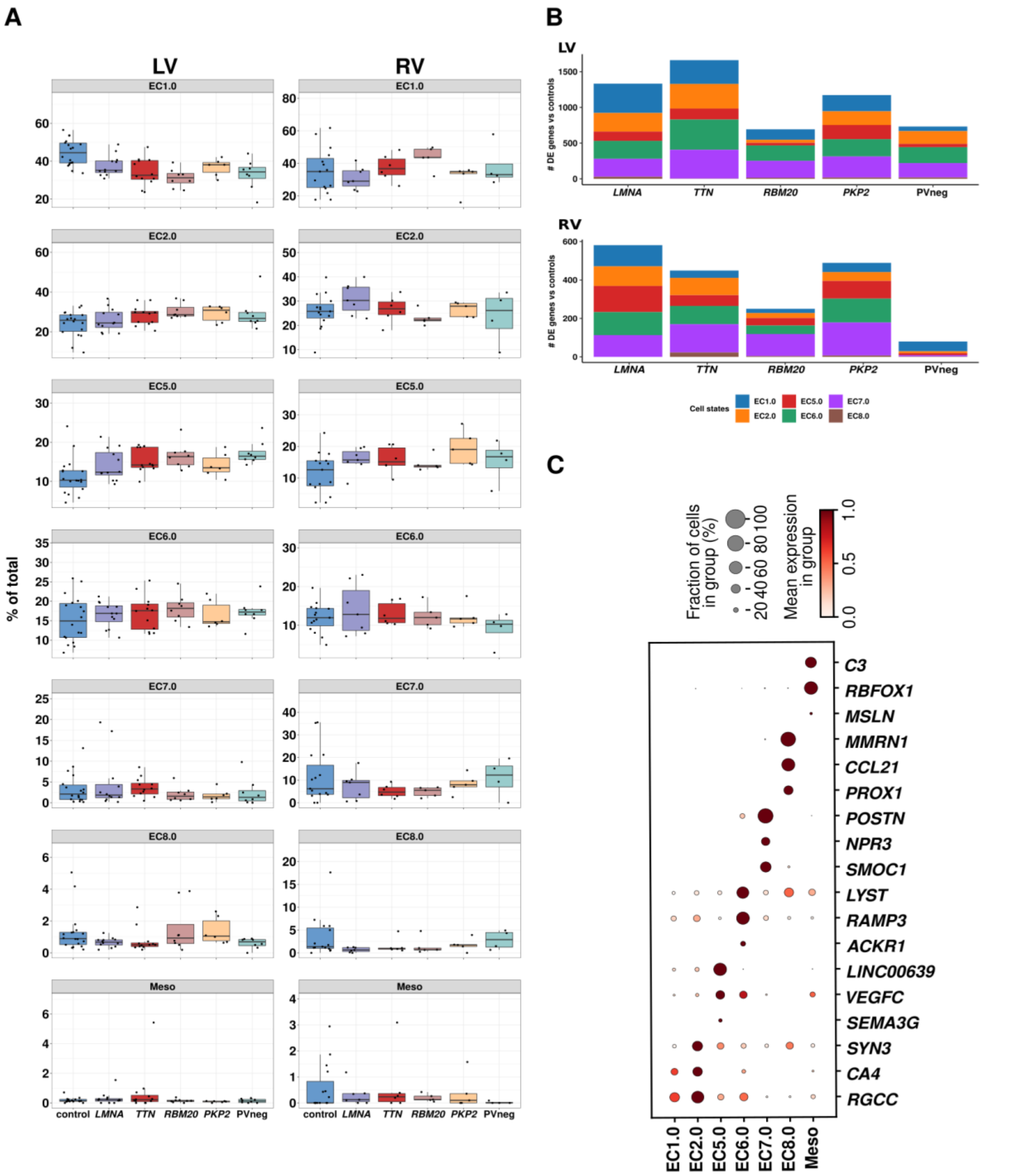

Figure S20: Characterization of endothelial cell (EC) state abundance and gene expression

(**A**) Box plots show EC state distribution across controls and genotypes in LVs and RVs. *p*-values are indicated for significant proportional changes, FDR<0.05. (**B**) Total number of upregulated genes across EC states and genotypes in LVs and RVs. Only significantly upregulated expressed genes (log2FC >0.5) are shown, FDR<0.05. (**C**) Dot plot shows selected marker genes of EC states. Dot size, fraction (%) of expressing cells; color, mean expression level.

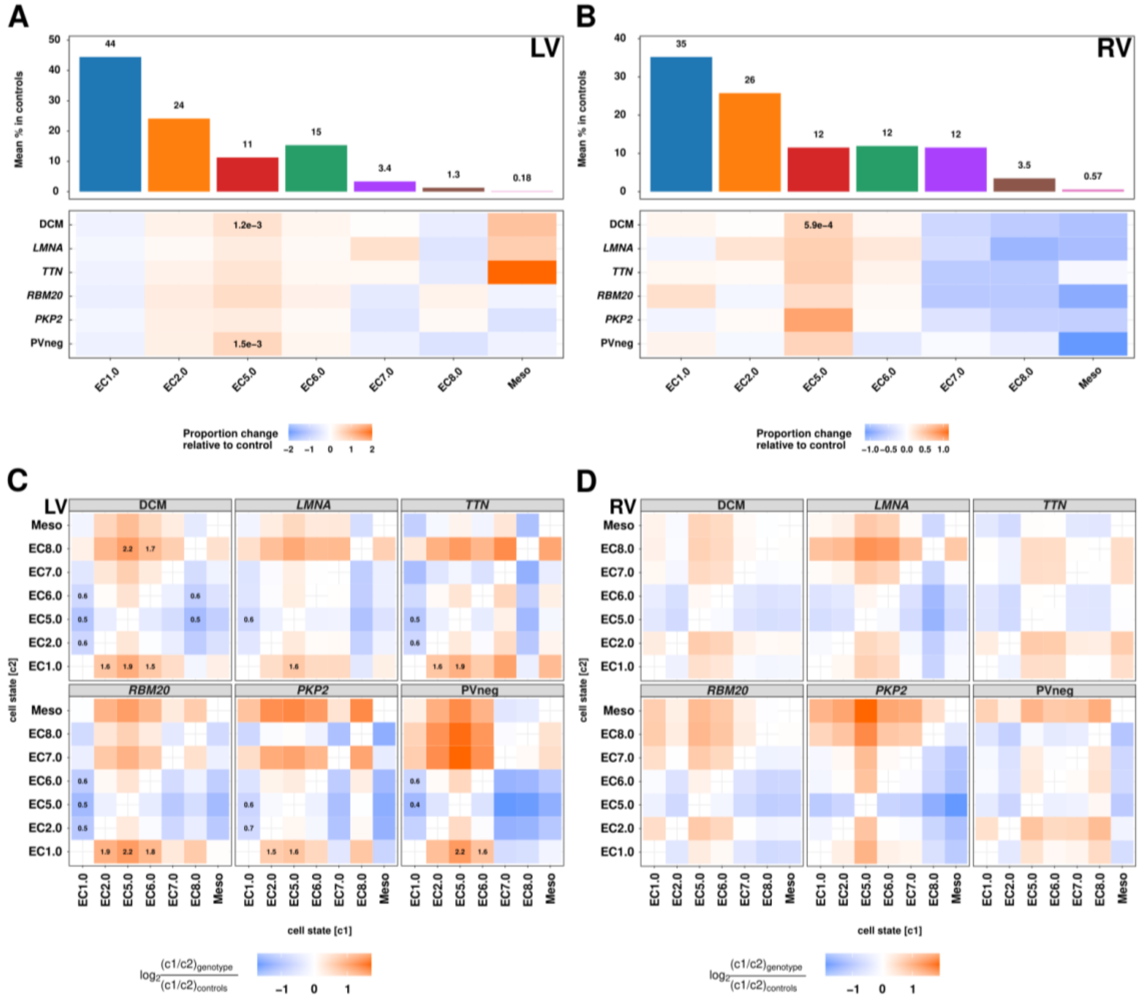

Figure S21: Genotype specific compositional changes in endothelial cells (ECs)

(**A**) Upper panel: Mean abundance (%) of EC states in control LVs. Lower panel: Proportional changes of EC states in specified genotypes or aggregated across DCM genotypes. (**B**) as in (**A**) but for RVs. (**C**) Pairwise EC state abundance ratios in specified genotypes or aggregated DCM genotypes in LVs relative to controls. (**D**) as in (C) but for RVs.

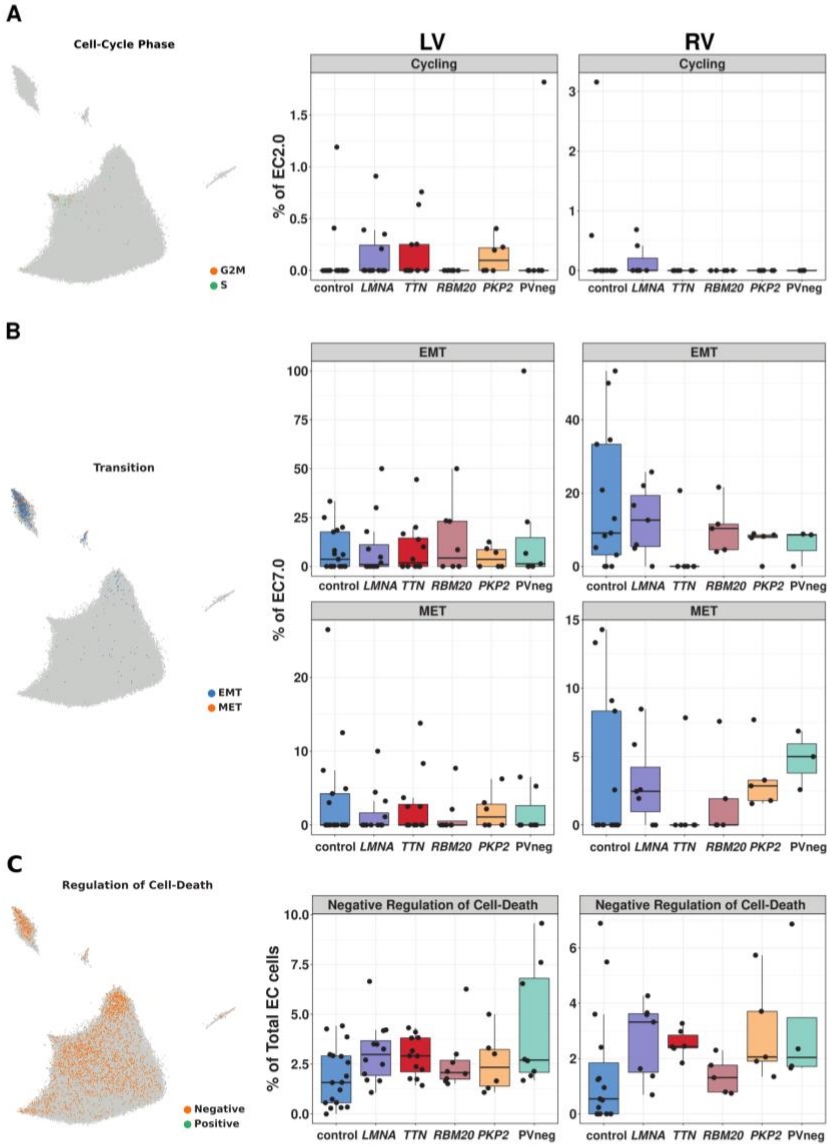

Figure S22: Cell-phase, EMT/MET, and apoptosis related pathways in endothelial cells (ECs)

(A) Cell-phase classification of ECs. Left: Nuclei in G2M (orange) and S-phase (green) are highlighted. Right: Each dot represents the abundance of cycling ECs of a patient in LV and RV. The table of genes used for classification is provided in Table SEC2. **(B)** EMT/MET classification of ECs. Left: Nuclei in EMT (blue) and MET (orange) are highlighted. Right: Each dot represents the abundance of transitional ECs of the EC7.0 population in LVs and RVs. The table of genes used is provided in Table SEC2. Nuclei with a score lower than 0.3 for both processes were considered as unscored and nuclei with a MET score higher than an EMT score were considered as undergoing MET. **(C)** Classification of ECs showing higher expression of positive and negative regulators of cell death. Right: Each dot represents the abundance of apoptosis inhibiting ECs of total EC population in LVs and RVs. The table of genes used is provided in Table SEC2. Nuclei with a score lower than 0.1 were unscored and nuclei with a greater positive than negative score were denoted as undergoing cell death.

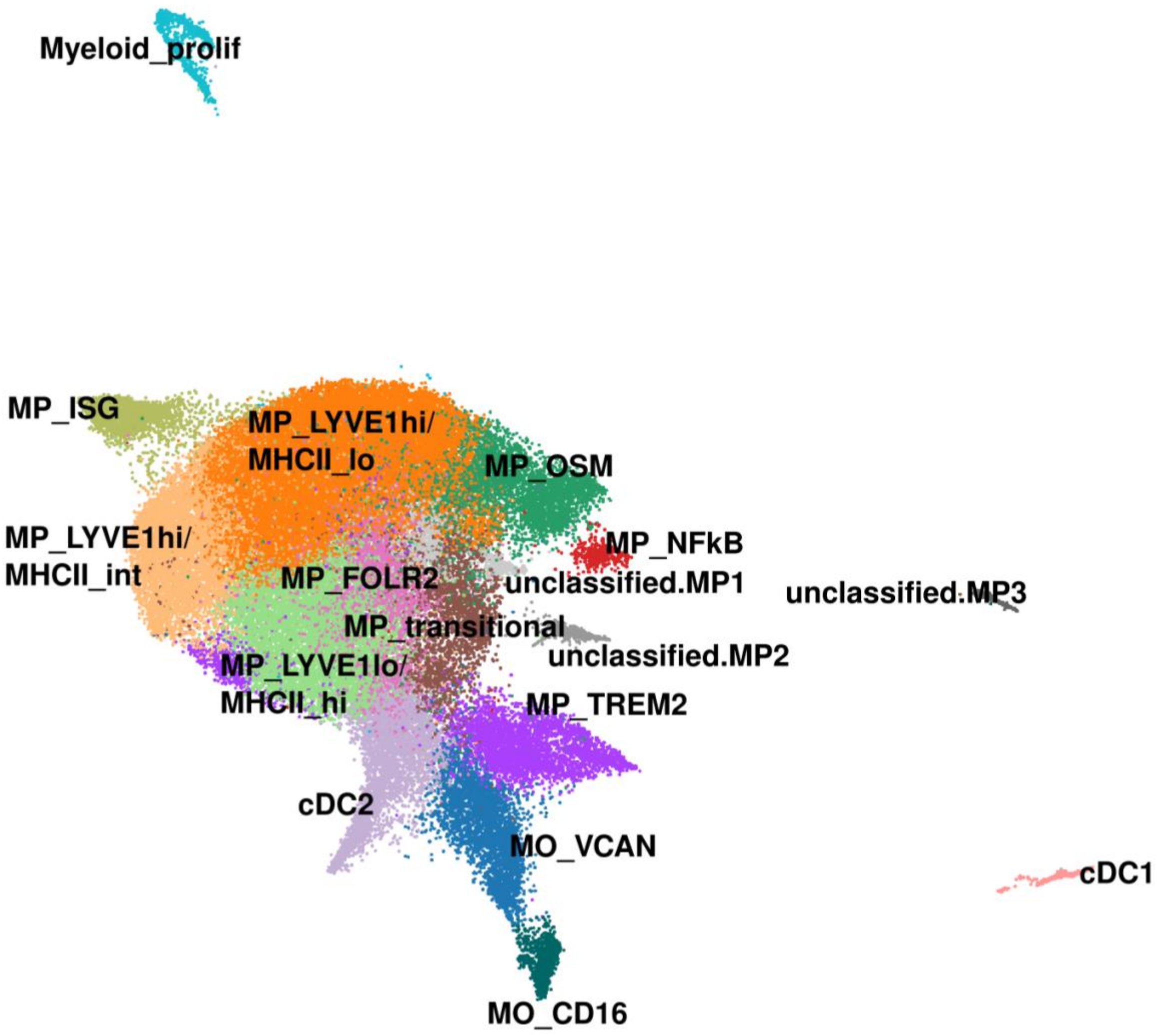

Figure S23: UMAP embedding and expression of selected marker genes of myeloid cells.

This is the unmodified UMAP of Fig. 4A, embedding the 17 myeloid states.

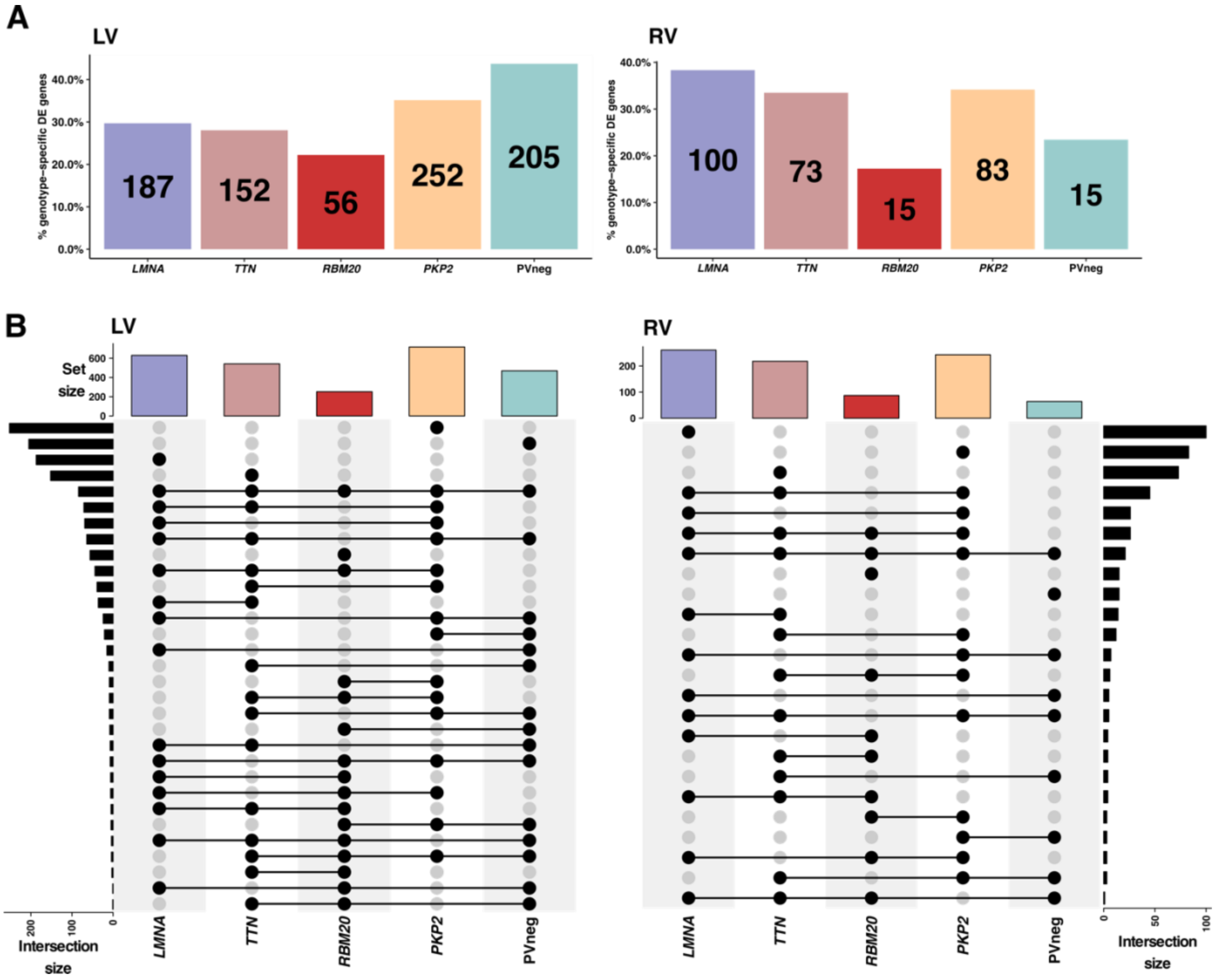

Figure S24: Genotype specific upregulated genes in myeloids

(A) Total number of uniquely upregulated genes (log2FC >0.5) for each genotype in LVs and RVs across all myeloid states, FDR<0.05. **(B)** Upset plots of all upregulated genes (log2FC>0.5, FDR<0.05) in LVs and RVs demonstrate shared (connected by lines) and specific expression (no connected lines) by genotypes. The total number of genes in the set is plotted as a bar on top (set size).

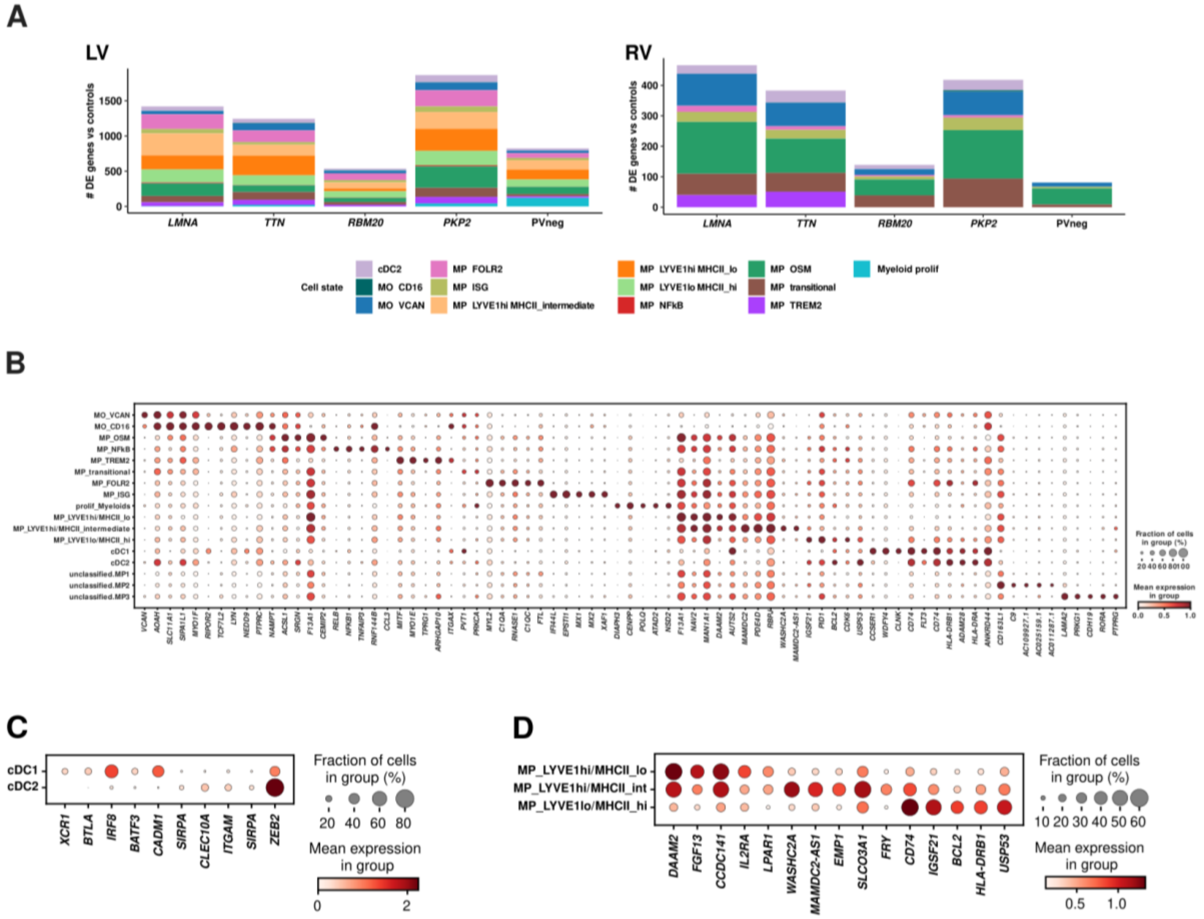

Figure S25: Characterization of myeloid state abundance

(A) Total number of upregulated genes across myeloid states and genotypes in LVs and RVs. Only significantly upregulated expressed genes (log2FC>0.5) are shown, FDR<0.05. **(B)** Dotplot shows selected marker genes of myeloid states. Dot size, fraction (%) of expressing cells; color, mean expression level. **(C)** Dotplot highlighting selected marker genes of cDC1 and cDC2. The dot sizes represent the fraction (%) of expressing cells; the color scale represents the corresponding mean expression levels. **(D)** Dotplot highlighting selected marker genes of different LYVE1 MP populations. The dot sizes represent the fraction (%) of expressing cells; the color scale represents the corresponding mean expression levels.

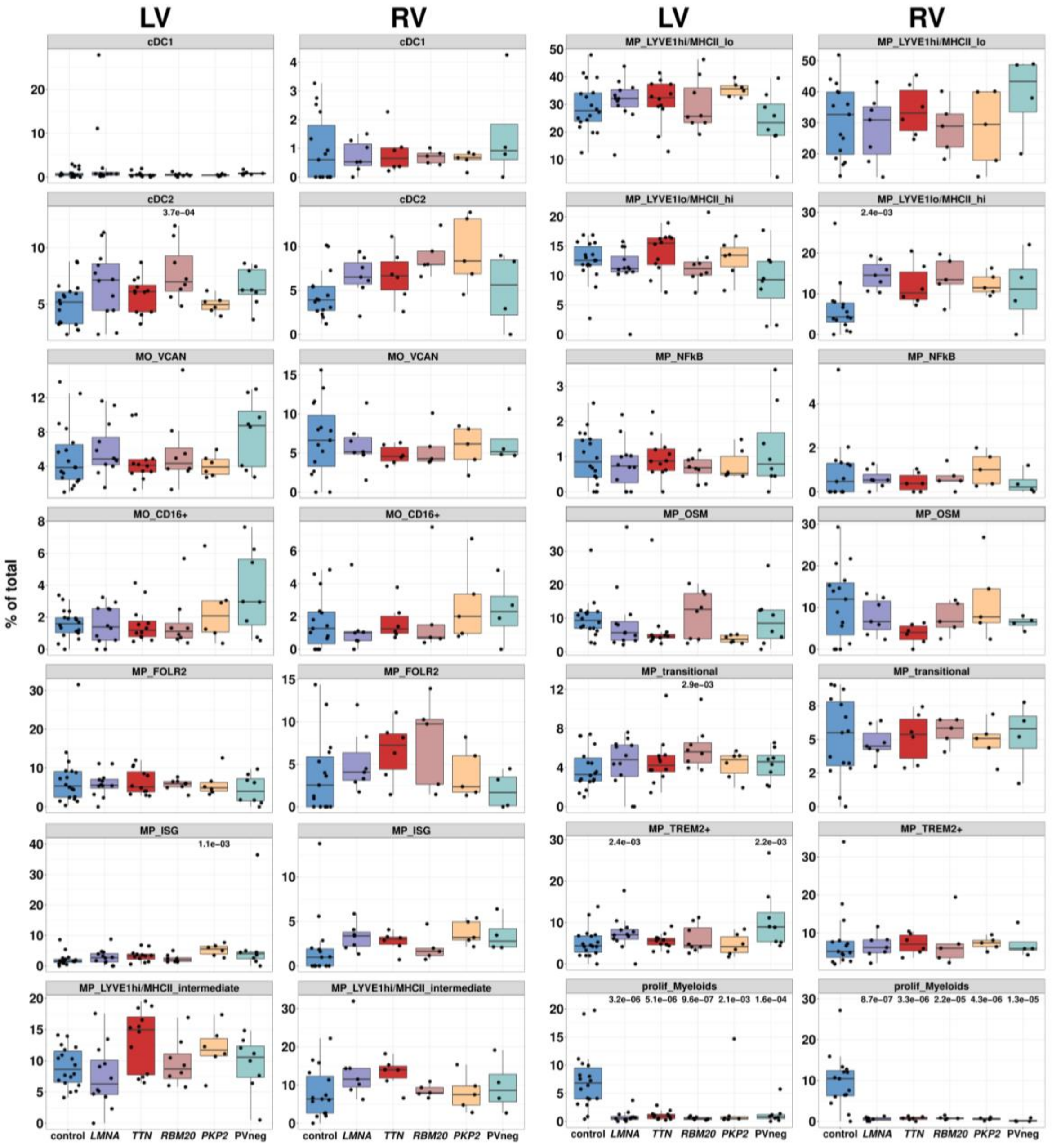

Figure S26: Characterization of myeloid state abundance

Box plots show myeloid state distribution across controls and genotypes in LVs and RVs. *p*-values are indicated for significant proportional changes, FDR<0.05.

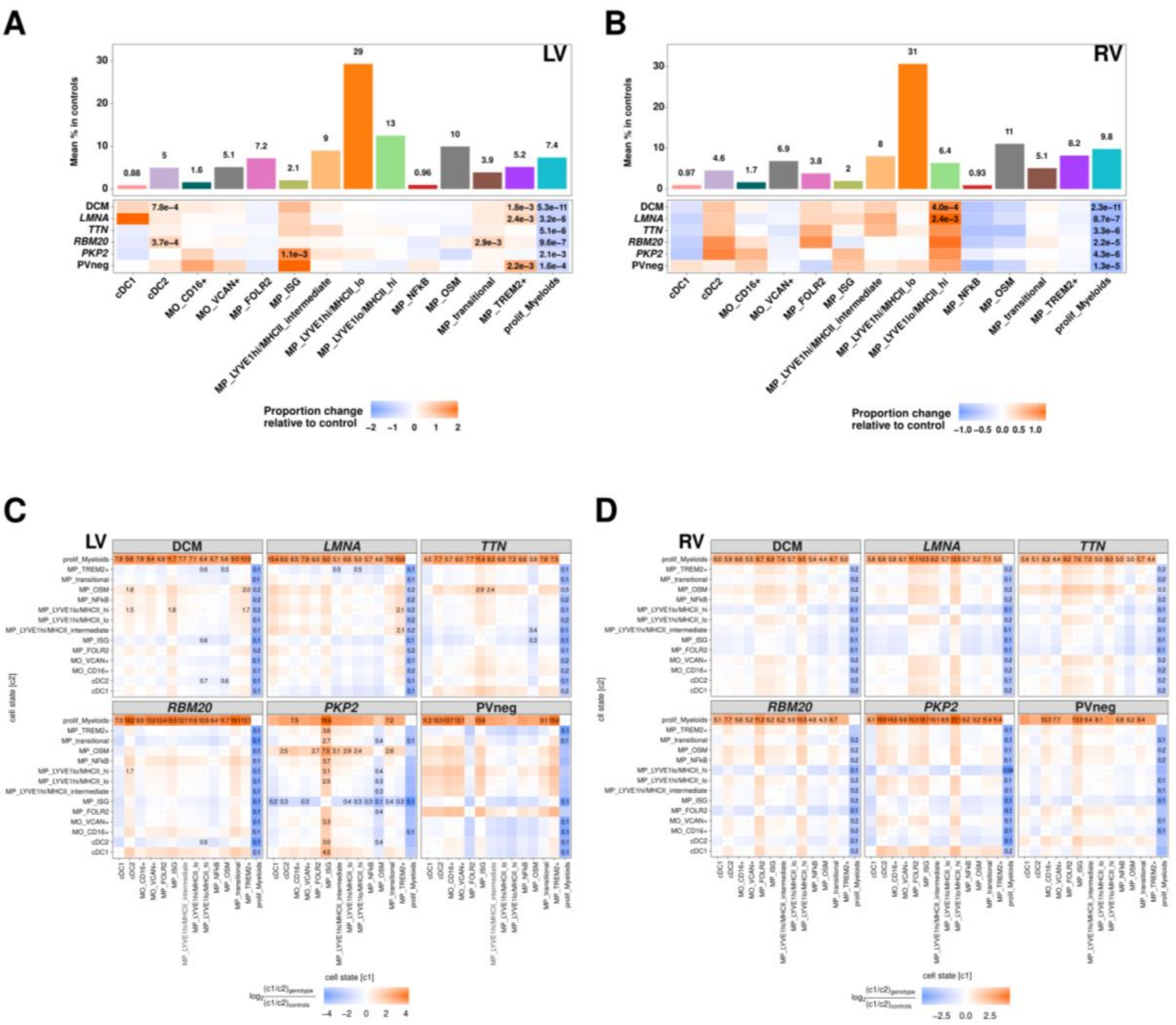

Figure S27: Genotype specific compositional changes in myeloids

(A) Upper panel: Mean abundance (%) of myeloid states in control LVs. Lower panel: Proportional changes of cardiomyocyte states in specified genotypes or aggregated across DCM genotypes. **(B)** as in (A) but for RVs. **(C)** Pairwise myeloid state abundance ratios in specified genotypes or aggregated DCM genotypes in LVs relative to controls**. (D)** as in (C) but for RVs.

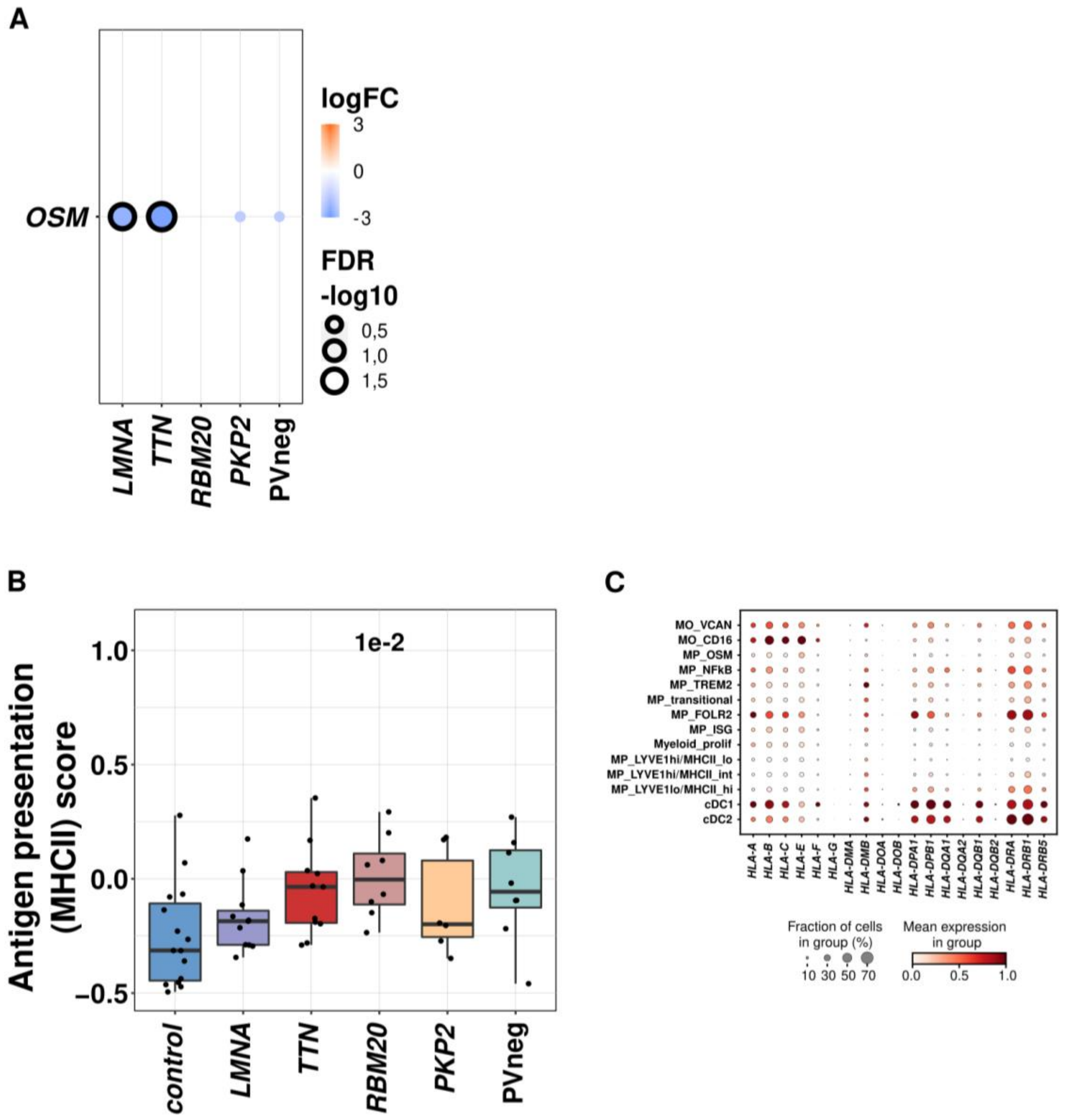

Figure S28: *OSM* and MHCII expression in myeloids

(A) Dot plot shows *OSM* expression across genotypes in LV MP_OSM compared to control. Dot sizes represents significance values; color intensity denotes fold-change. **(B)** Enrichment score of antigen presentation MHCII gene expression in LVs across antigen presenting myeloid populations and genotypes. cDC1, cDC2, MO_CD16, MO_VCAN, MP_FOLR2 and MP_LYVE1lo/MHCII_hi are included. (**C)** Dotplot highlighting MHCI and II genes across all assigned myeloid states (all regions and genotypes). The dot sizes represent the fraction (%) of expressing cells; the color scale represents the corresponding mean expression levels.

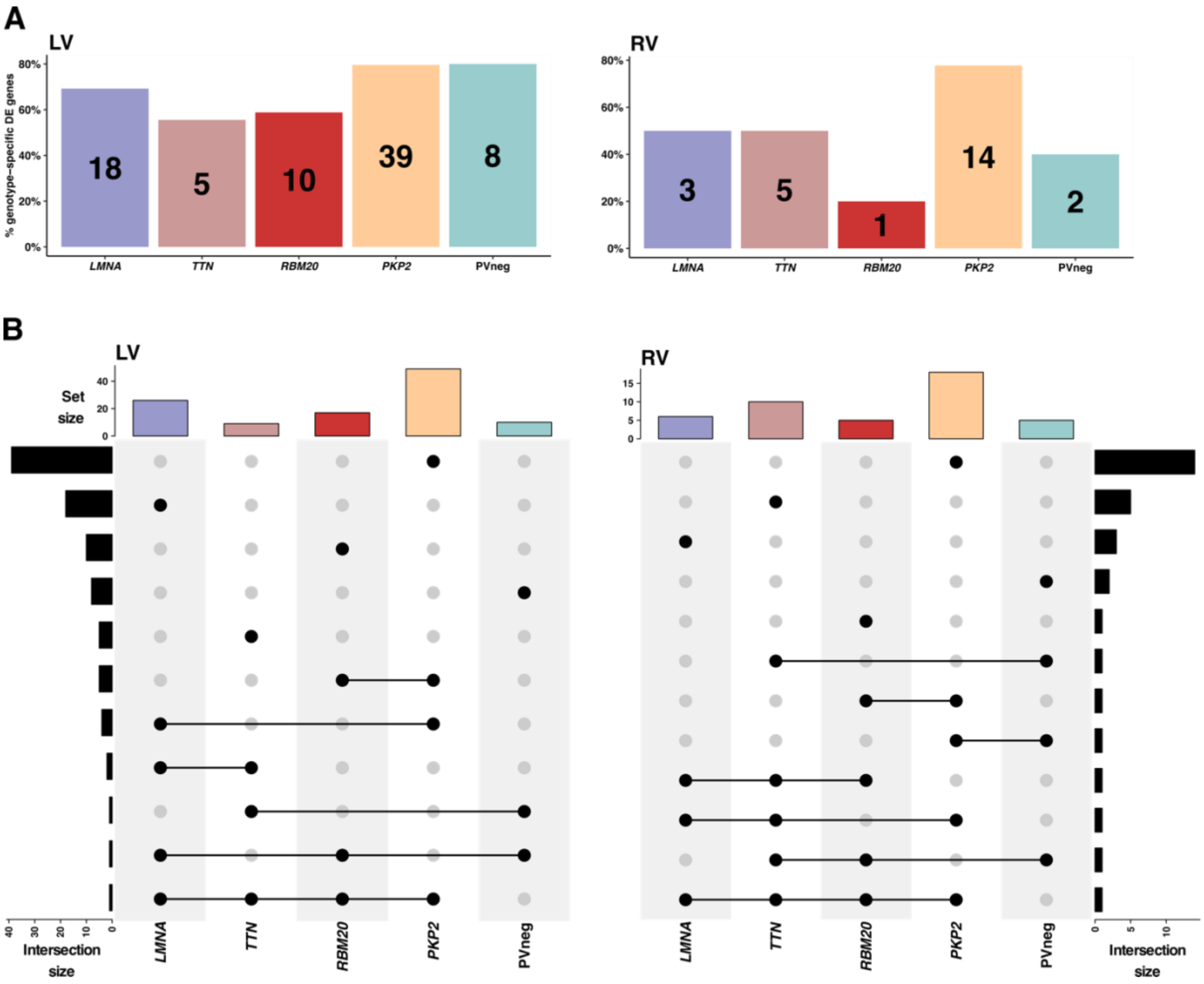

Figure S29: Genotype specific upregulated genes in lymphoids

(A) Total number of uniquely upregulated genes (log2FC>0.5) for each genotype in LVs and RVs across all lymphoid states, FDR<0.05. **(B)** Upset plots of all upregulated genes (log2FC>0.5, FDR<0.05) in LVs and RVs demonstrated shared (connected by lines) and specific expression (no connected lines) by genotypes. The total number of genes in the set is plotted as a bar on top (set size).

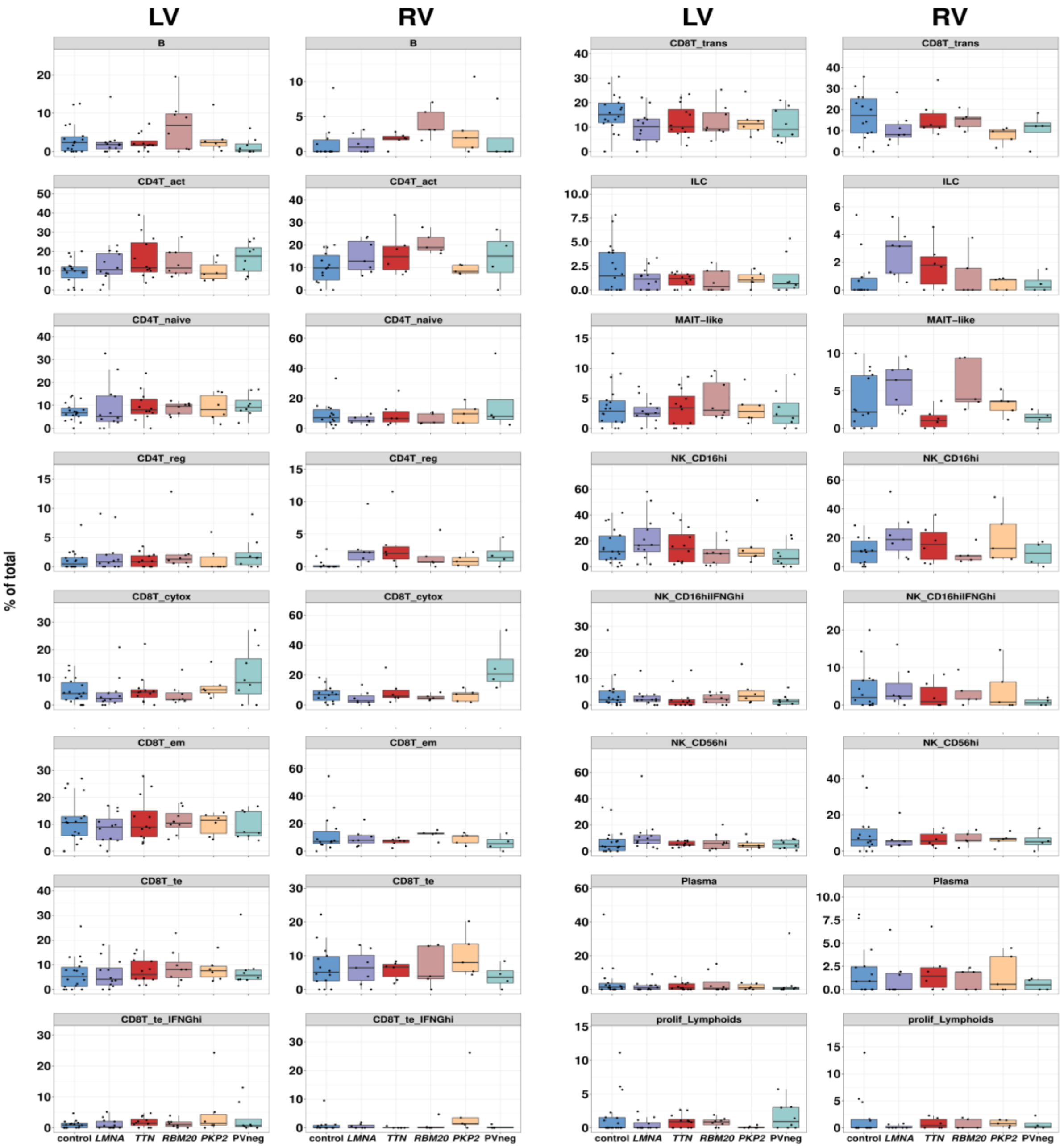

Figure S30: Characterization of lymphoid state abundance

Box plots showing myeloid state distribution across controls and genotypes in LVs and RVs. *p*-values are indicated for significant proportional changes, FDR<0.05.

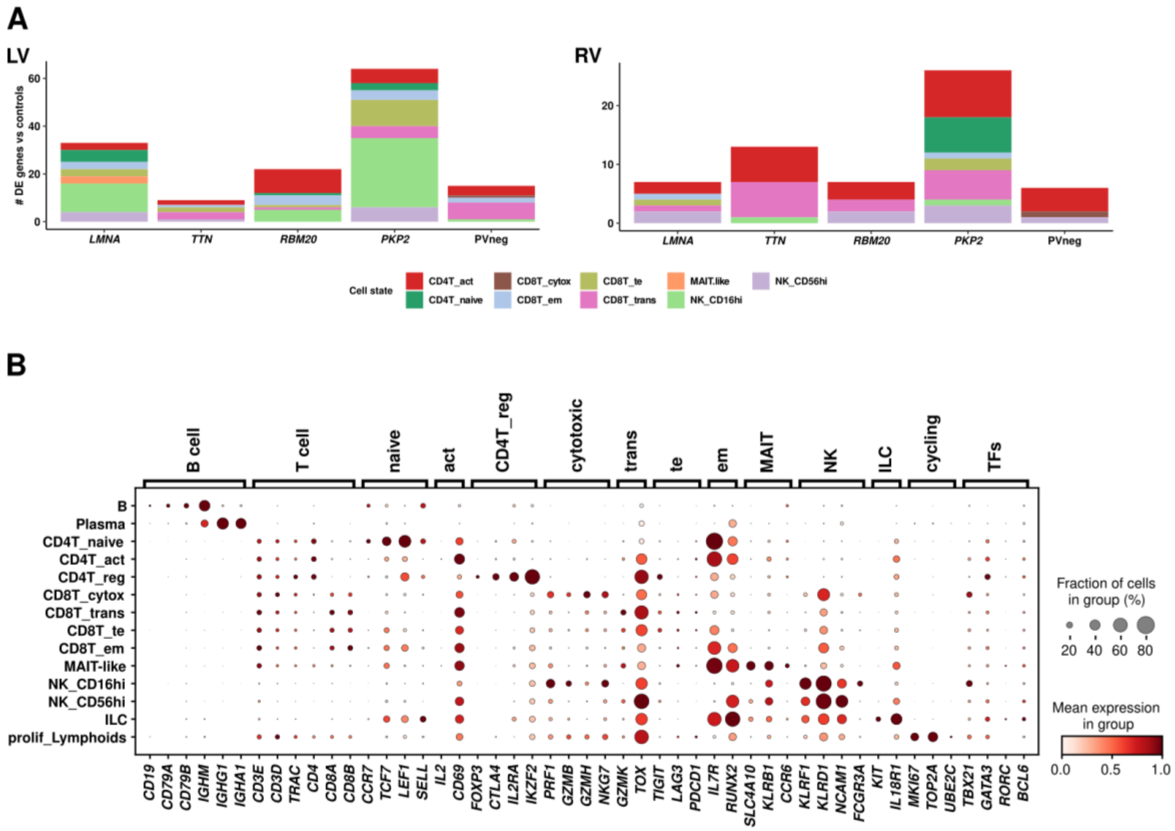

Figure S31: Characterization of lymphoid state gene expression

(A) Total number of upregulated genes across lymphoid states and genotypes in LVs and RVs. Only significantly upregulated expressed genes (log2FC>0.5) are shown, FDR<0.05. **(B)** Dotplot shows selected marker genes of lymphoid states. Dot size, fraction (%) of expressing cells; color, mean expression level.

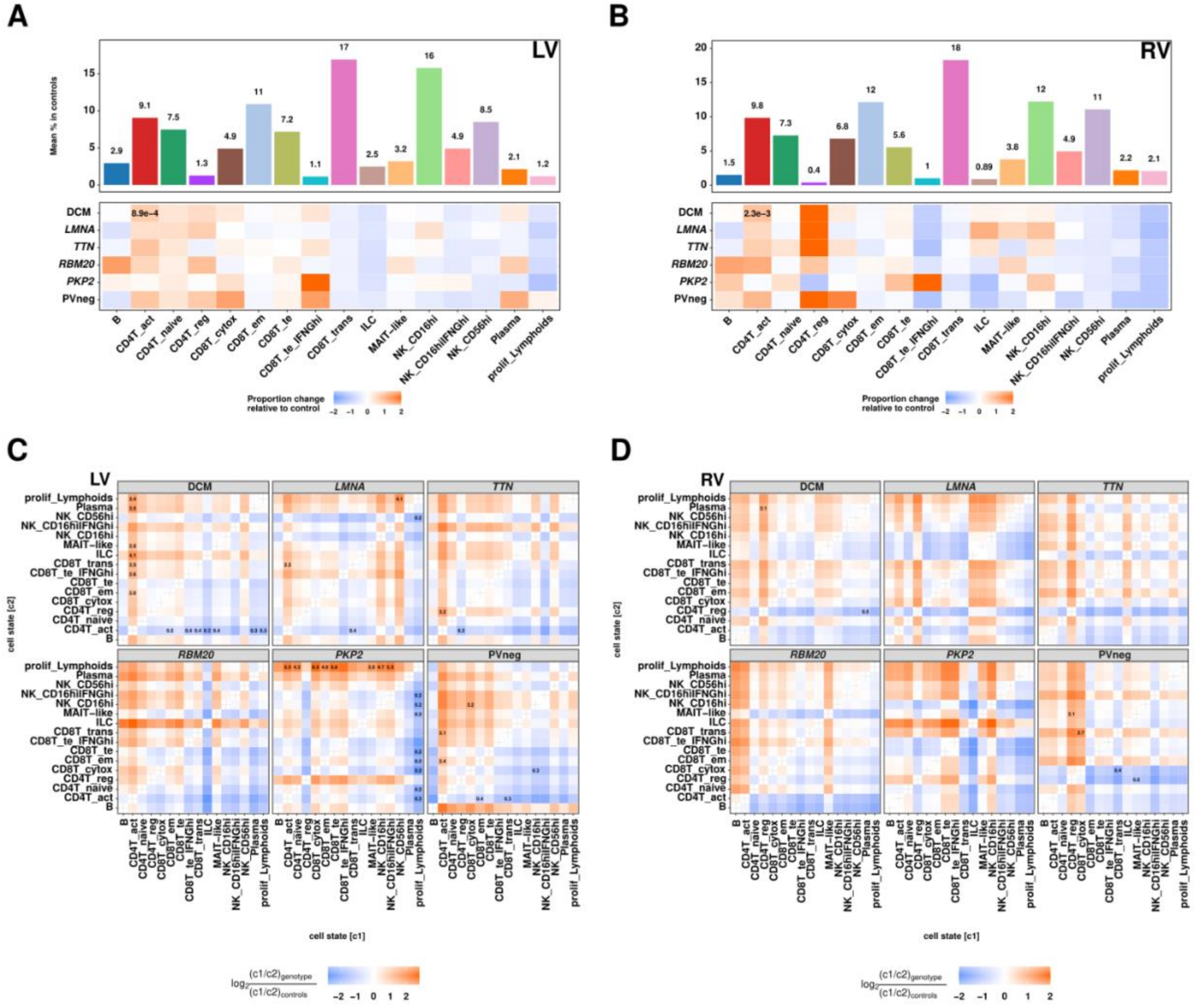

Figure S32: Genotype specific compositional changes in lymphoids

(A) Upper panel: Mean abundance (%) of lymphoids states in control LVs. Lower panel: Proportional changes of lymphoid states in specified genotypes or aggregated across DCM genotypes. **(B)** as in (A) but for RVs. **(C)** Pairwise lymphoid state abundance ratios in specified genotypes or aggregated DCM genotypes in LVs relative to controls. **(D)** as in (C) but for RVs.

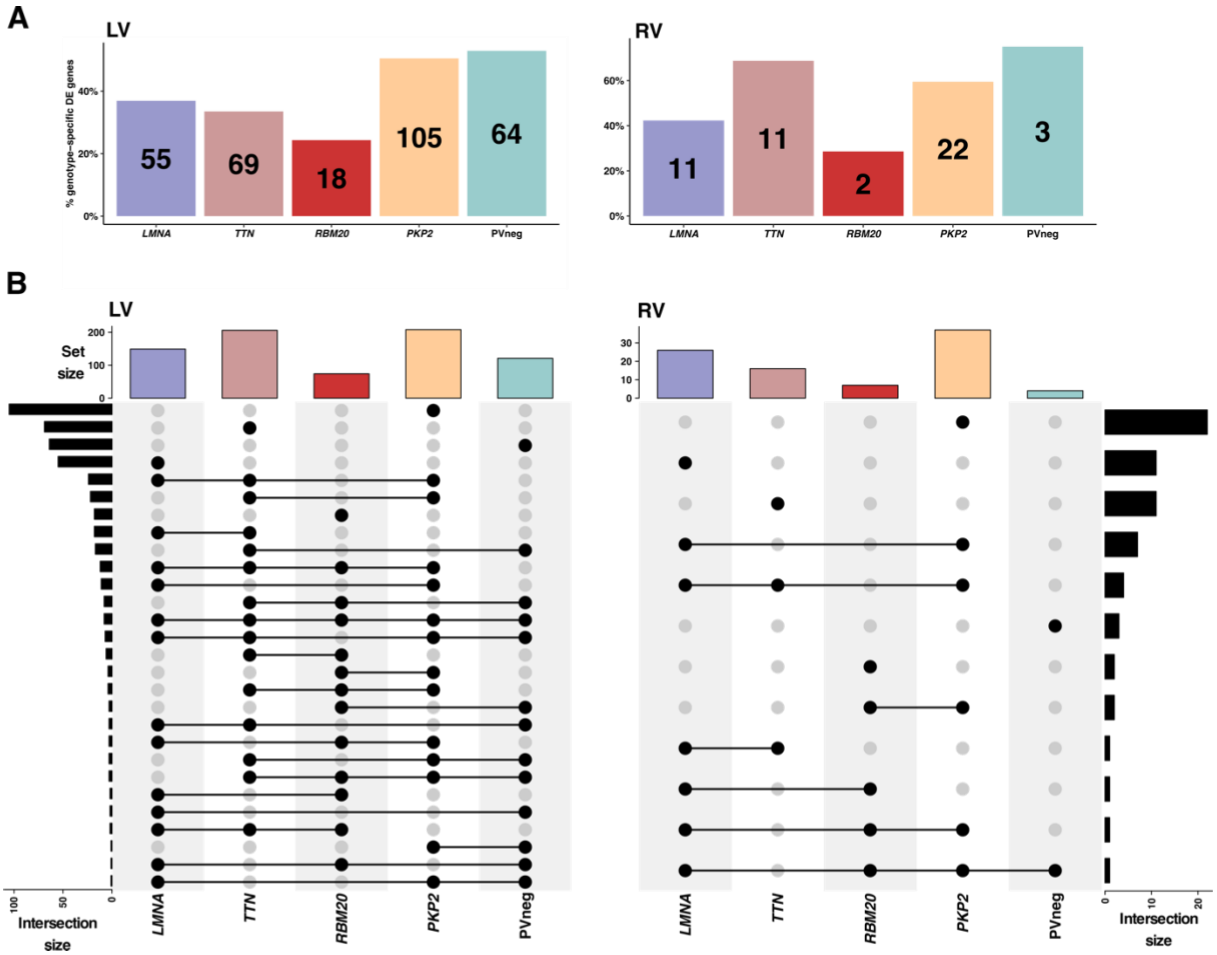

Figure S33: Genotype specific upregulated genes in neuronal cells (NC)

(A) Total number of uniquely upregulated genes (log2FC>0.5) for each genotype in LVs and RVs across all NC states, FDR<0.05. **(B)** Upset plots of all upregulated genes (log2FC>0.5, FDR<0.05) in LVs and RVs demonstrated shared (connected by lines) and specific expression (no connected lines) by genotypes. The total number of genes in the set is plotted as a bar on top (set size).

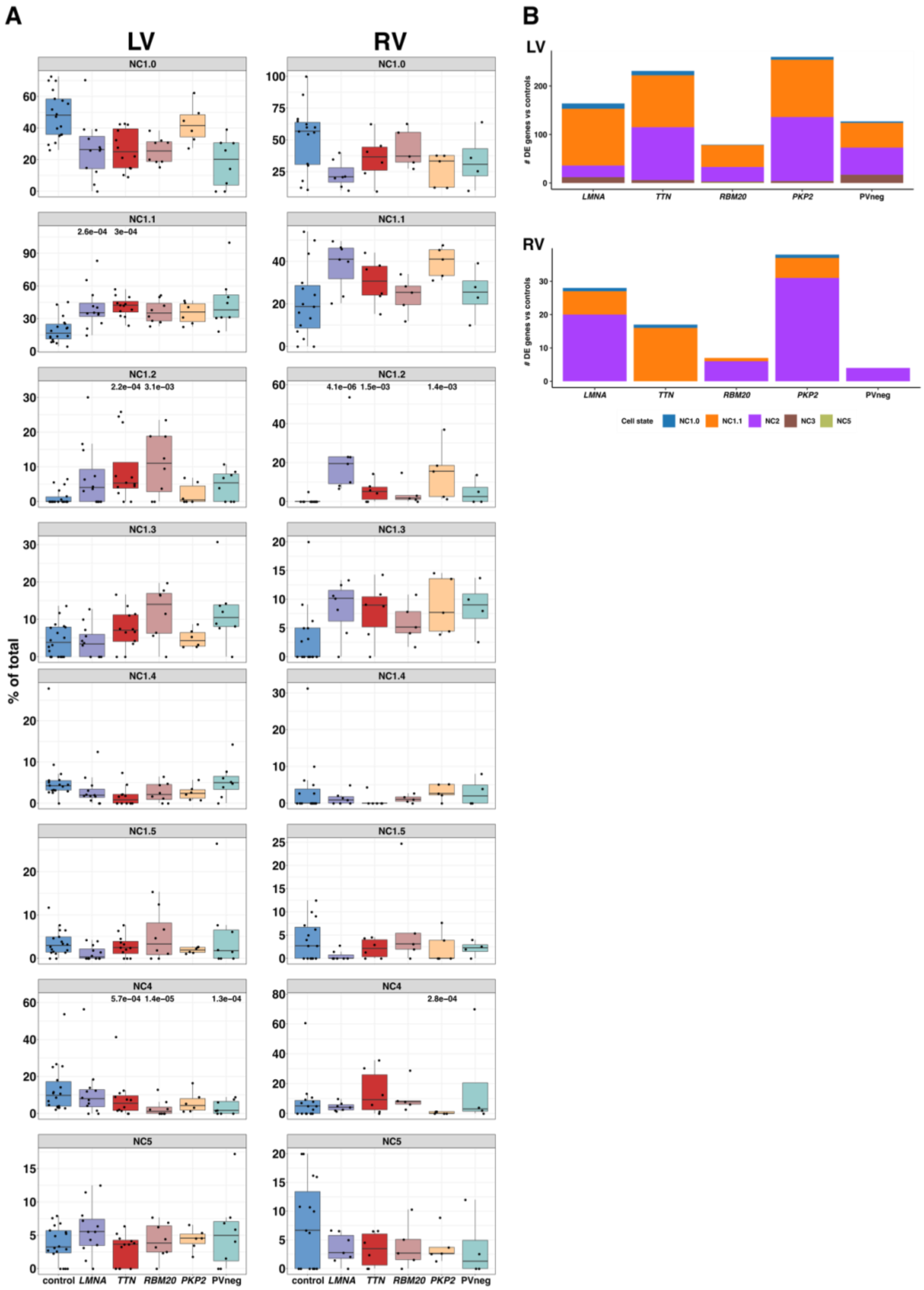

Figure S34: Characterization of neuronal cell (NC) state abundance and gene expression

(A) Box plots show NC state distribution across controls and genotypes in LVs and RVs. *p*-values are indicated for significant proportional changes, FDR<0.05. **(B)** Total number of upregulated genes across NC states and genotypes in LVs and RVs. Only significantly upregulated expressed genes (log2F >0.5) are shown, FDR<0.05.

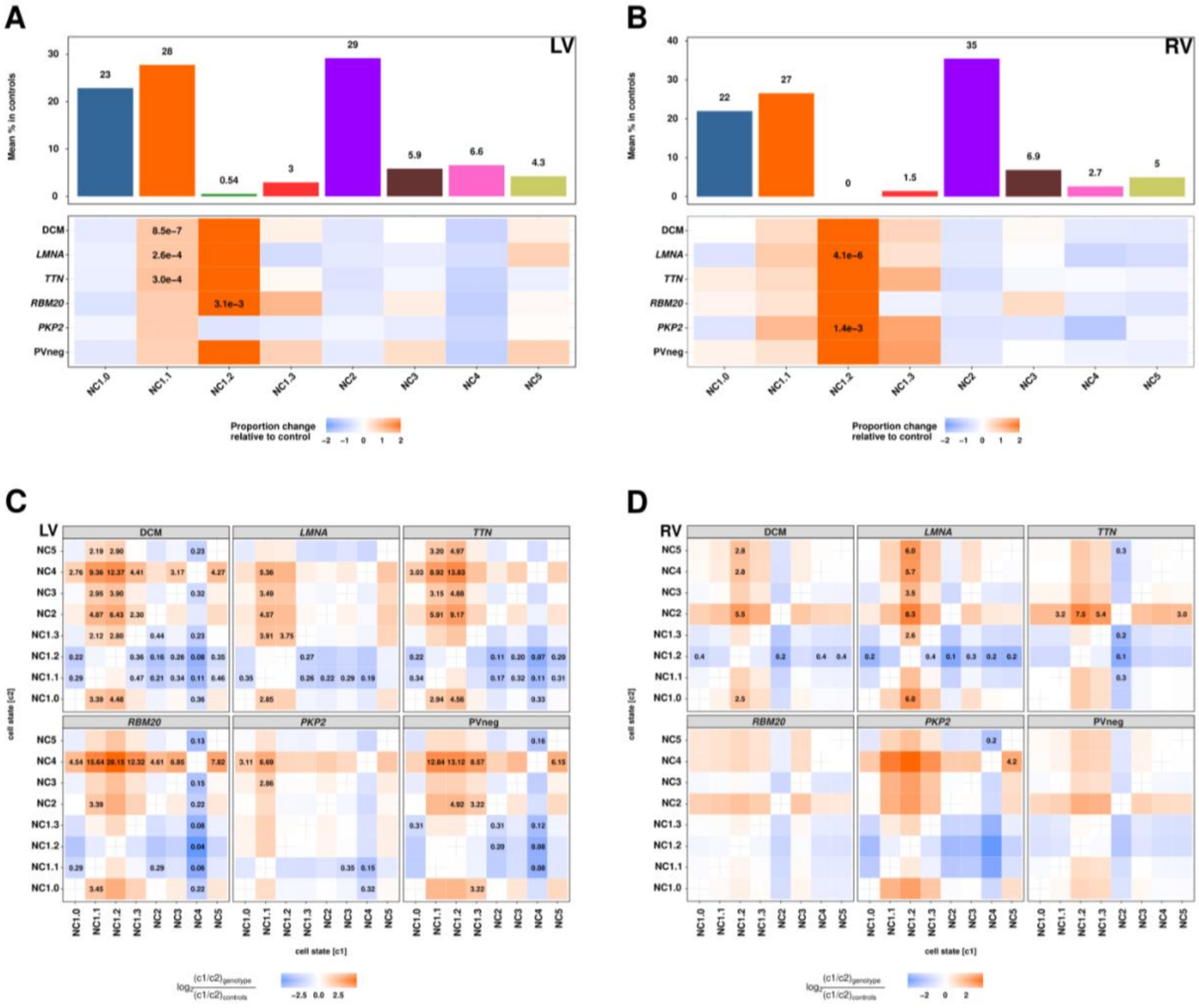

Figure S35: Genotype specific compositional changes in neuronal cells (NC)

(A) Upper panel: Mean abundance (%) of NC states in control LVs. Lower panel: Proportional changes of NC states in specified genotypes or aggregated across DCM genotypes. **(B)** as in (A) but for RVs**. (C)** Pairwise NC state abundance ratios in specified genotypes or aggregated DCM genotypes in LVs relative to controls. **(D)** as in (C) but for RVs.

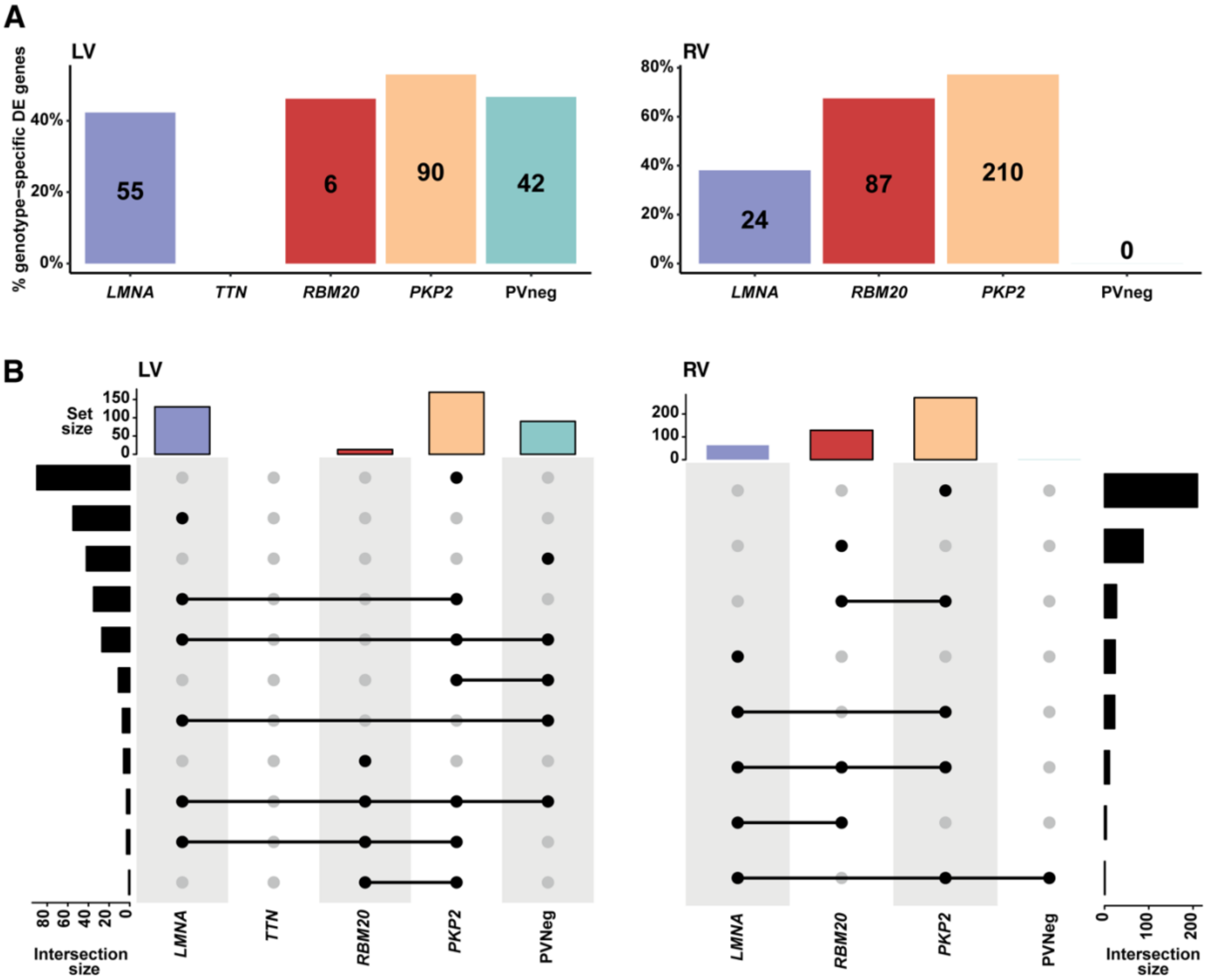

Figure S36: Genotype specific upregulated genes in adipocytes

(A) Total number of uniquely upregulated genes (log2FC>0.5) for each genotype in LVs and RVs across all adipocytes, FDR<0.05. **(B)** Upset plots of all upregulated genes (log2FC>0.5, FDR<0.05) in LVs and RVs demonstrated shared (connected by lines) and specific expression (no connected lines) by genotypes. The total number of genes in the set is plotted as a bar on top (set size).

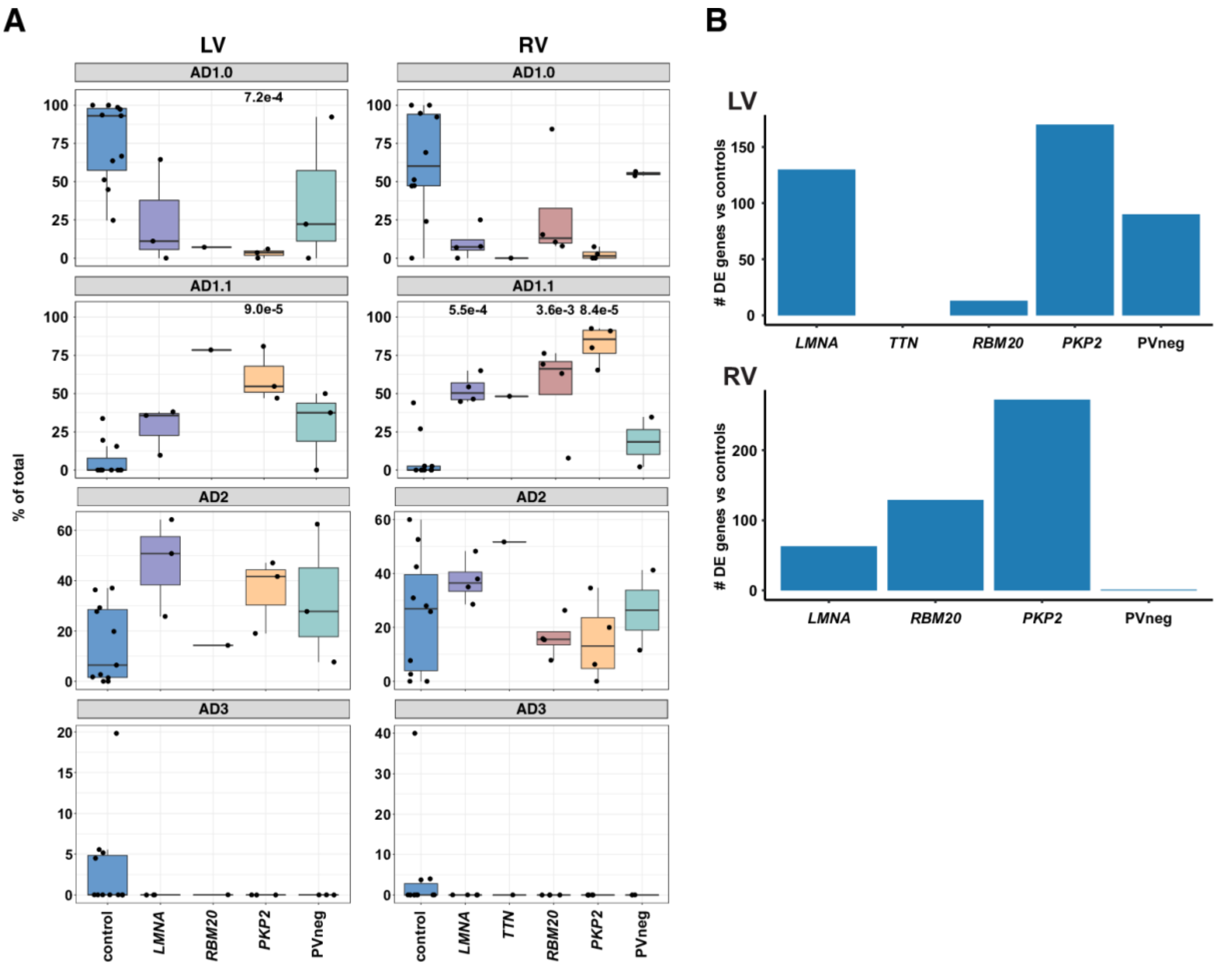

Figure S37: Characterization of adipocyte state abundance

(**A**) Box plots show adipocyte state distribution across controls and genotypes in LVs and RVs. Tissues from patients with fewer than 10 nuclei were excluded from these analyses. *p*-values are indicated for significant proportional changes, FDR<0.05. (**B**) Total number of upregulated genes across adipocyte states and genotypes in LVs and RVs. Only significantly upregulated expressed genes (log2FC>0.5) are shown, FDR<0.05.

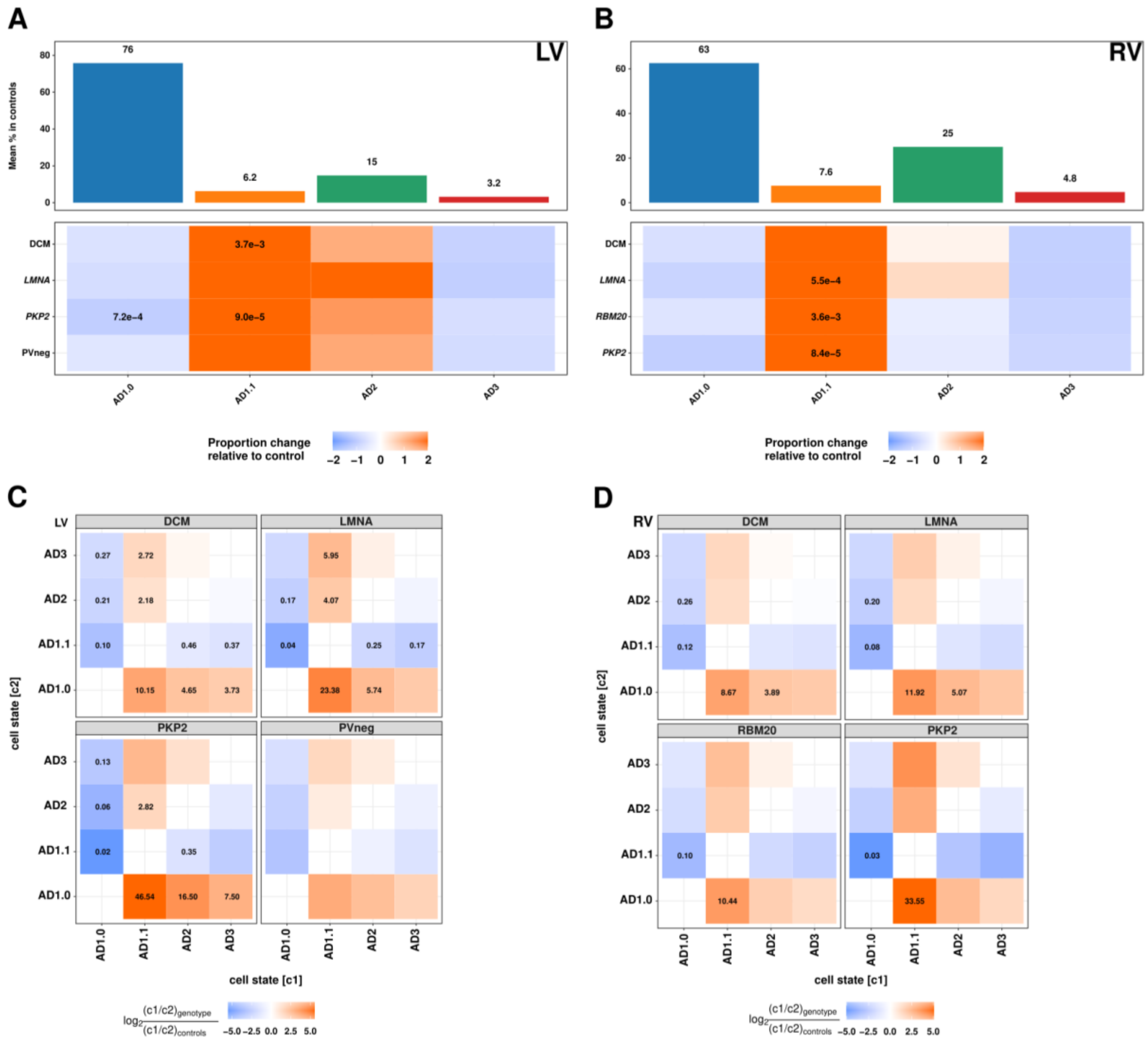

Figure S38: Genotype specific compositional changes in adipocytes

(**A**) Upper panel: Mean abundance (%) of adipocyte states in control LVs. Lower panel: Proportional changes of adipocyte states in specified genotypes or aggregated across DCM genotypes. (**B**) as in (A) but for RVs. (**C**) Pairwise adipocyte state abundance ratios in specified genotypes or aggregated DCM genotypes in LVs relative to controls. (**D**) as in (C) but for RVs.

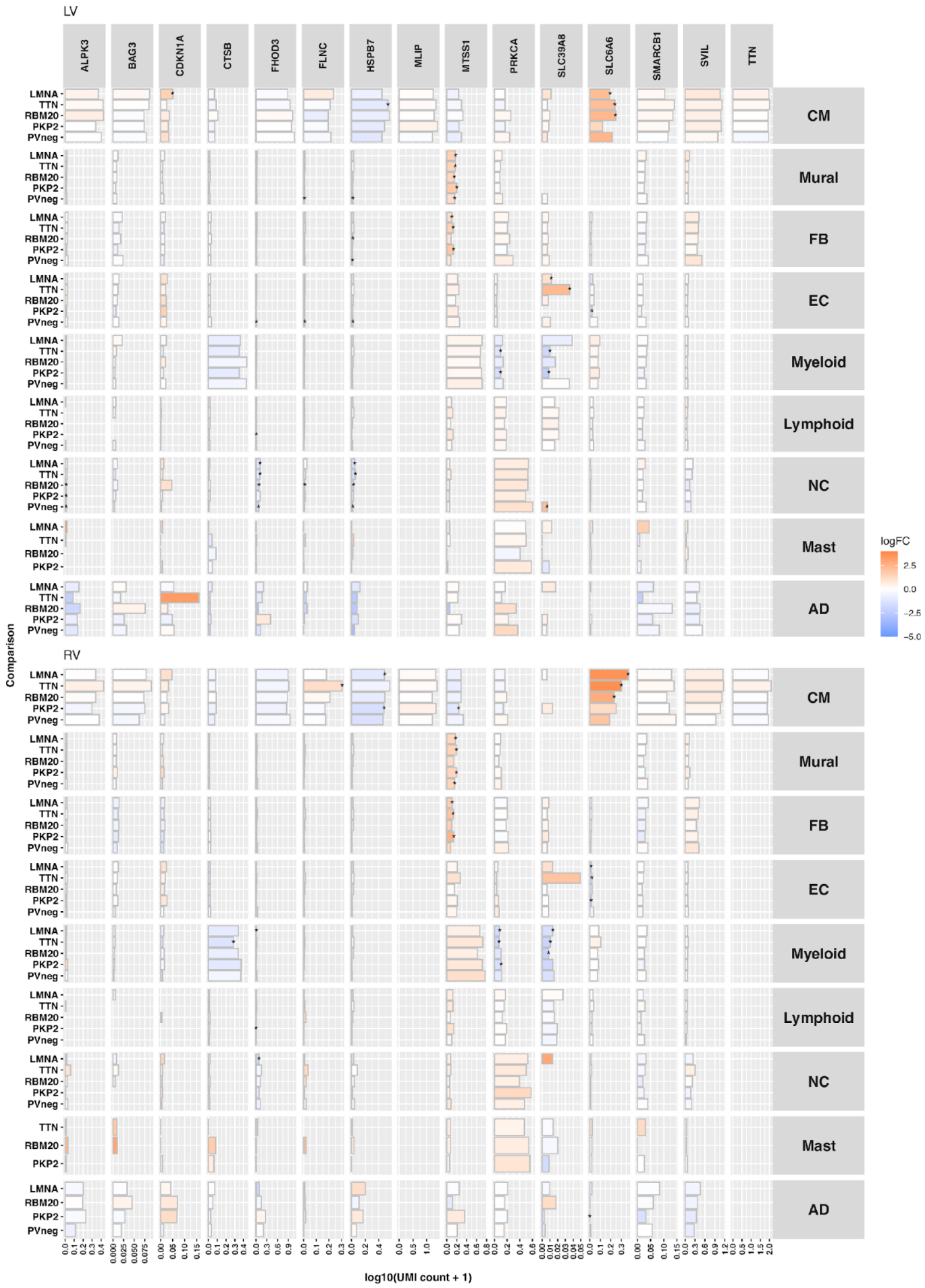

LV
RV
ALPK3
BAG3
CDKN1A
CTSB
FHOD3
FLNC
HSPB7
MLIP
MTSS1
PRKCA
SLC39A8
SLC6A6
SMARCB1
SVIL
TTN
LMNA
TTN
RBM20
PKP2
PVneg
CM
Mural
FB
EC
Myeloid
Lymphoid
NC
Mast
AD
logFC
2.5
0.0
-2.5
-5.0
Comparison
log10(UMI count + 1)

Figure S39. **LV and RV expression of genes identified by DCM GWAS studies in DCM and ACM samples.** The expression of genes that were identified from published GWAS studies (Table S65) are shown as log(UMI count +1), fold-changes are relative to mean pseudobulk expression of the control group, per gene and cell type (Supplemental Methods). Note different scales are used between genes in order to account for variable ranges of expression. Blue-orange shading represents log fold change in expression compared to controls. *denotes $p<0.05$. Nuclei number for Mast cells in PVneg was too small to calculate expression changes.

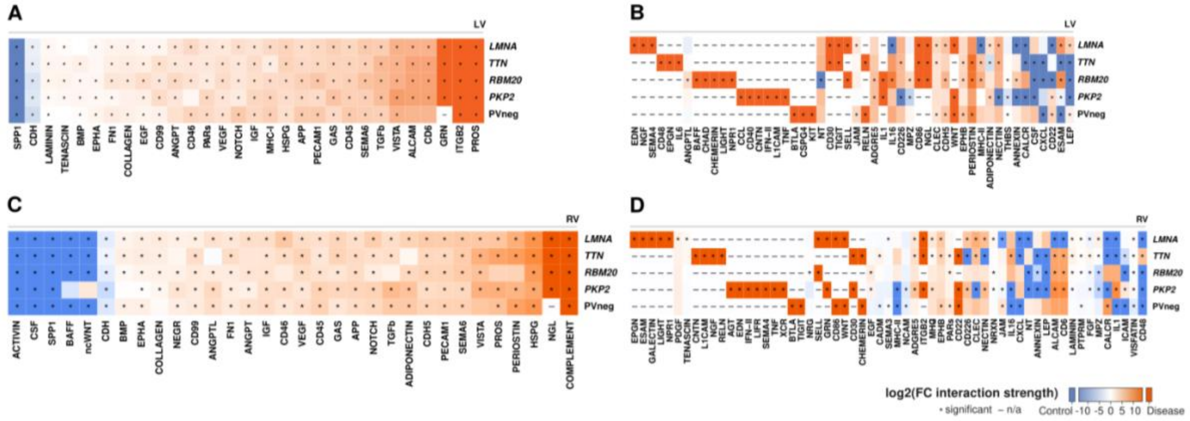

Figure S40: **Altered cell-cell interactions**

Heatmaps depict common (**A: LV, C:RV**) and unique (**B: LV, D: RV**) signaling pathways that are significantly expressed in diseased hearts. Changes in interaction strength (log2(fold-change)), scaled by color intensity (red, increased; blue, decreased). *denotes significance; adjusted *p*-values≤0.05; n/a denotes expression not detected in control or disease.

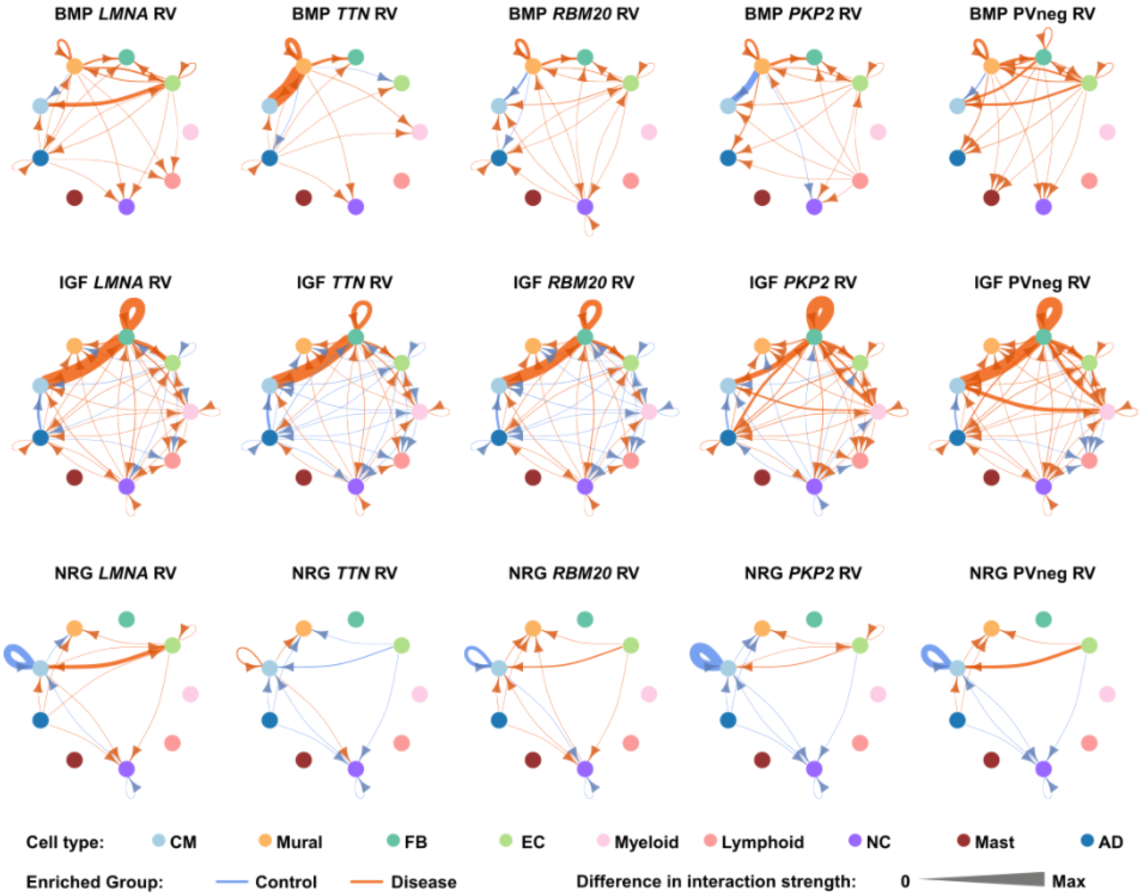

Figure S41: Schematic representation cell-cell communications for BMP, IGF, and NRG pathways in diseased RVs.

Circle plots of significant (adjusted *p*-value≤0.05) cell-cell communications depict differentially regulated bone morphogenic protein (BMP), insulin growth factor (IGF) and neuregulin (NRG) pathways and interactions in disease RVs. The line thickness denotes interaction strength of signals from sending and receiving cell types, with color (orange, increased; blue, decreased) scaled from zero to maximum in diseased versus controls. Arrows indicate directionality.

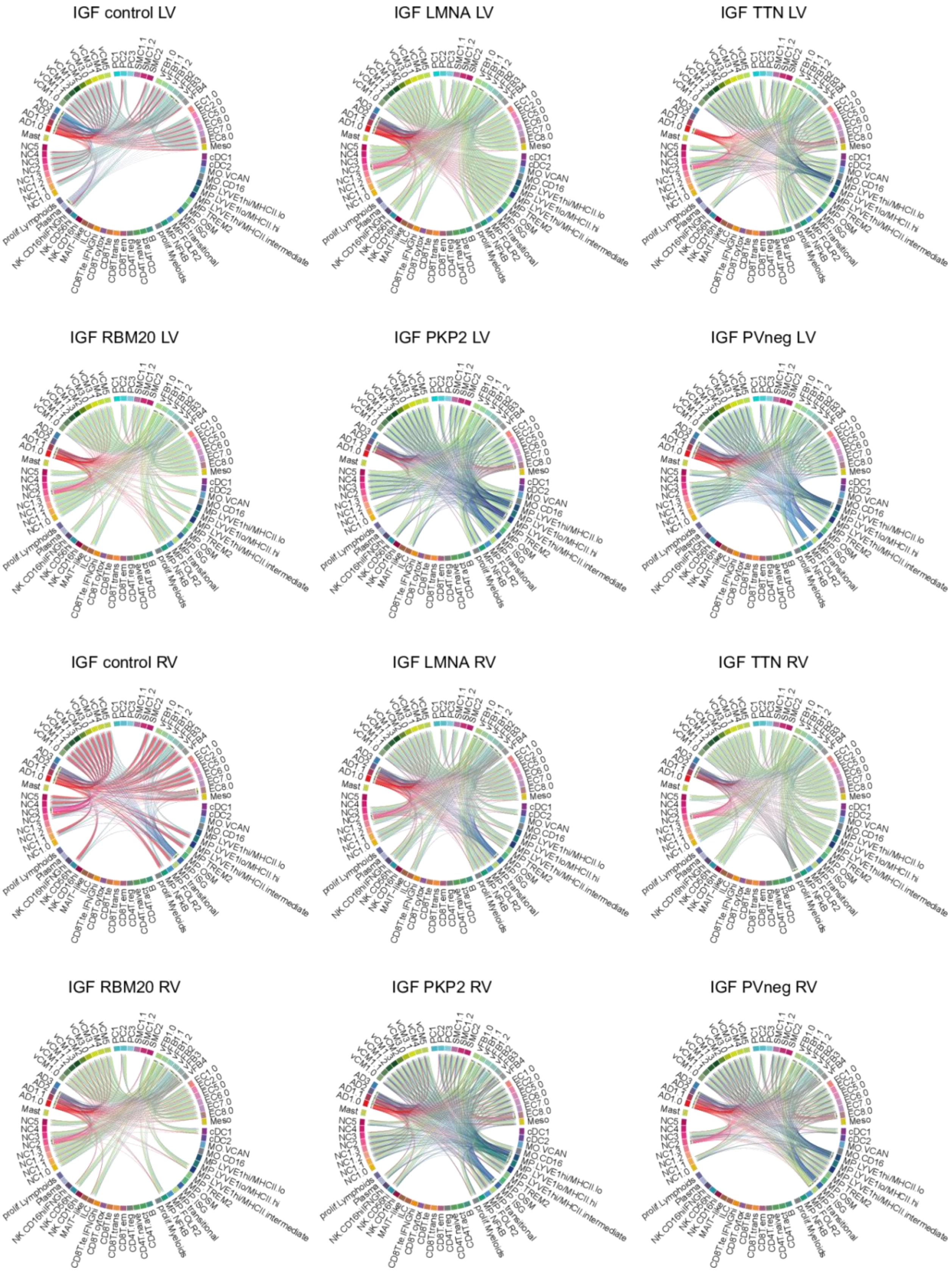
IGF control LV
IGF LMNA LV
IGF TTN LV
IGF RBM20 LV
IGF PKP2 LV
IGF PVneg LV
IGF control RV
IGF LMNA RV
IGF TTN RV
IGF RBM20 RV
IGF PKP2 RV
IGF PVneg RV

Figure S42: Representation of cell-cell interactions for the IGF pathway in LVs and RVs with different genotypes

Chord plots of significant (adjusted *p*-value≤0.05) cell-cell communications depict the differentially regulated insulin growth factor (IGF) pathway and interactions in disease LVs and RVs. The line thickness denotes interaction strength of signals from sending and receiving cell types, with color (orange, increased; blue, decreased) scaled from zero to maximum in diseased versus controls. Arrows indicate directionality.

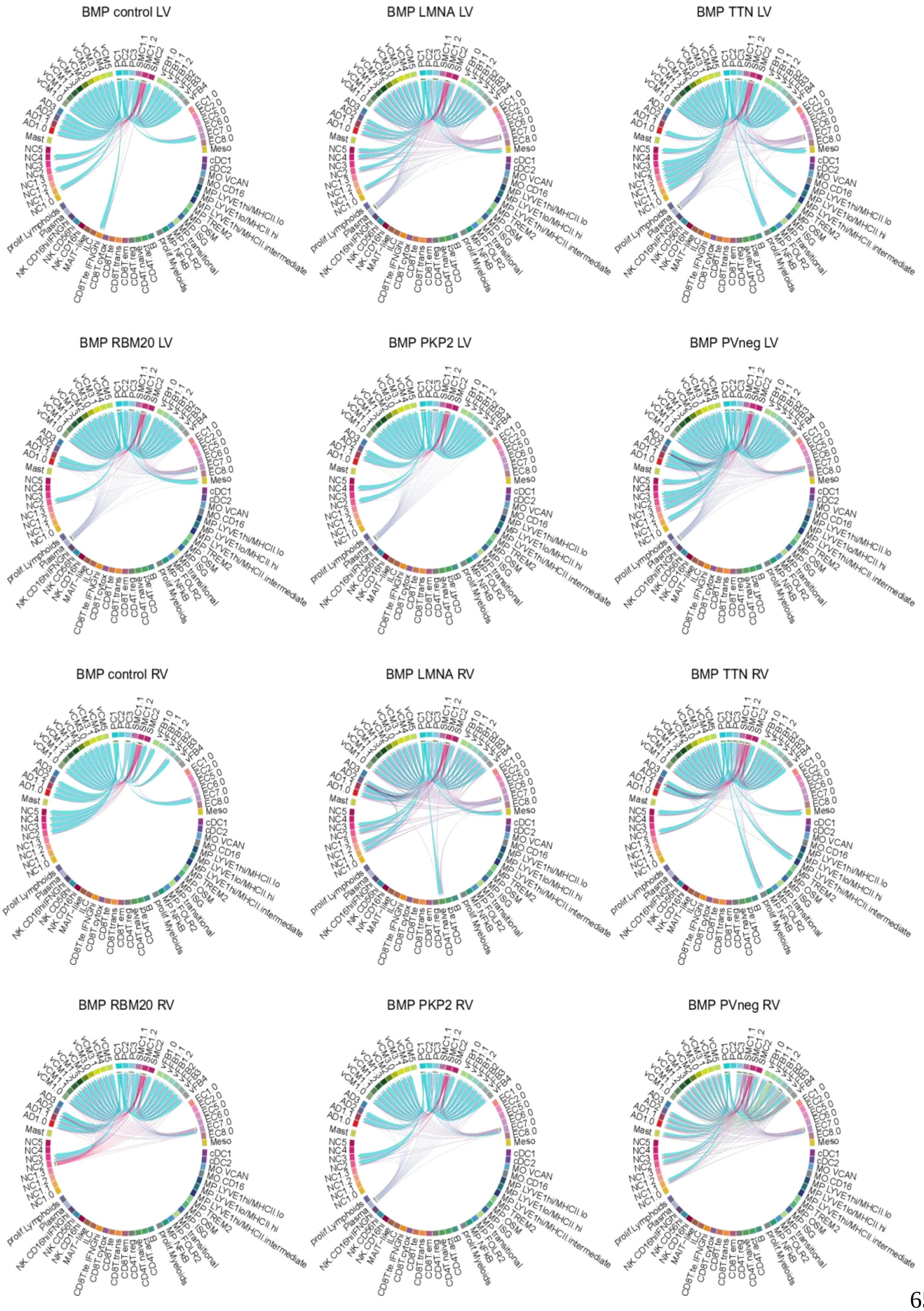
BMP control LV
BMP LMNA LV
BMP TTN LV
BMP RBM20 LV
BMP PKP2 LV
BMP PVneg LV
BMP control RV
BMP LMNA RV
BMP TTN RV
BMP RBM20 RV
BMP PKP2 RV
BMP PVneg RV

Figure S43: Representation of cell-cell interactions for the BMP pathway in LVs and RVs with different genotype

Chord plots of significant (adjusted *p*-value≤0.05) cell-cell communications depict the differentially regulated bone morphogenic protein (BMP) pathway and interactions in disease LVs and RVs. The lines thickness denotes interaction strength of signals from sending and receiving cell types, with color (orange, increased; blue, decreased) scaled from zero to maximum in diseased versus controls. Arrows indicate directionality.

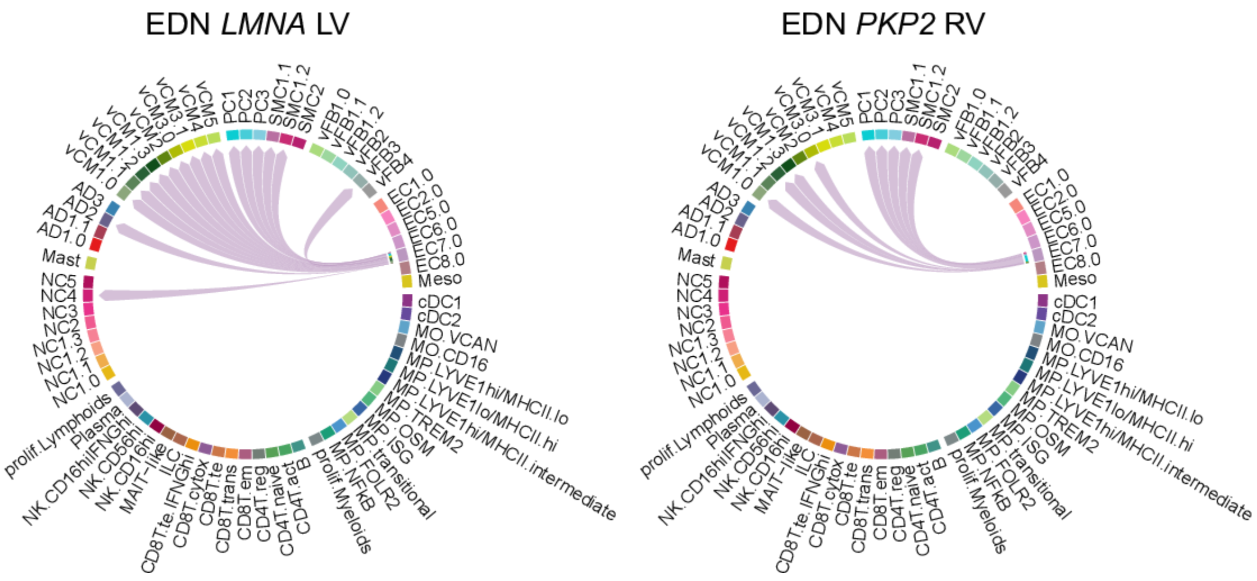

Figure S44: Representation of cell-cell interactions for the EDN pathway in *LMNA* LVs and *PKP2* RVs

Chord plots of significant (adjusted *p*-value≤0.05) cell-cell communications depict the differentially regulated neuregulin (NRG) pathway and interactions in disease LVs and RVs. The lines thickness denotes interaction strength of signals from sending and receiving cell types, with color (orange, increased; blue, decreased) scaled from zero to maximum in diseased versus controls. Arrows indicate directionality.

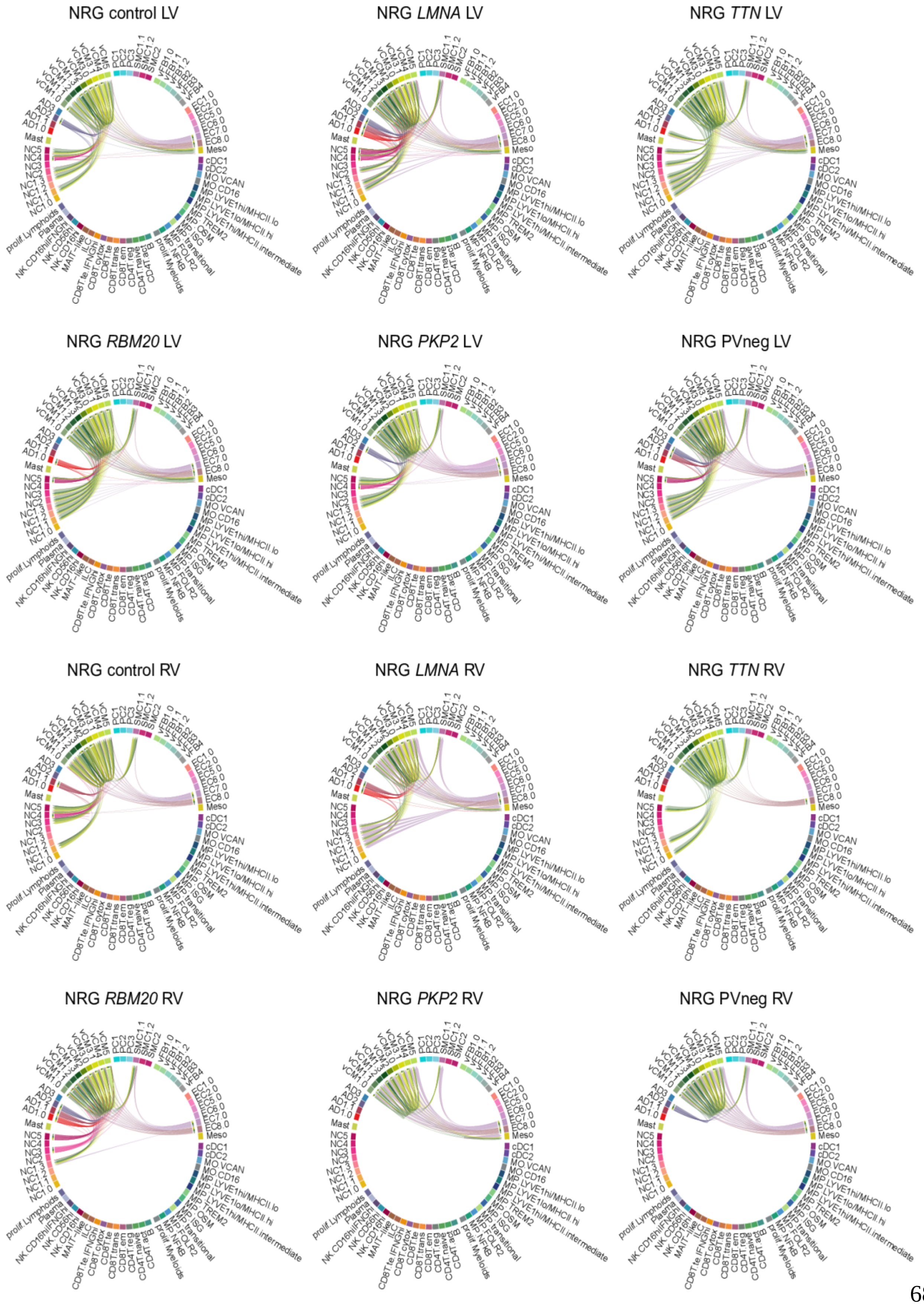
NRG control LV
NRG *LMNA* LV
NRG *TTN* LV
NRG *RBM20* LV
NRG *PKP2* LV
NRG PVneg LV
NRG control RV
NRG *LMNA* RV
NRG *TTN* RV
NRG *RBM20* RV
NRG *PKP2* RV
NRG PVneg RV

Figure S45: Representation of cell-cell interactions for the NRG pathway in LVs and RVs with different genotypes

Chord plots of significant (adjusted *p*-value≤0.05) cell-cell communications depict the differentially regulated endothelin (EDN) pathway and interactions in disease LVs and RVs. The line thickness denotes interaction strength of signals from sending and receiving cell types, with color (orange, increased; blue, decreased) scaled from zero to maximum in diseased versus controls. Arrows indicate directionality.

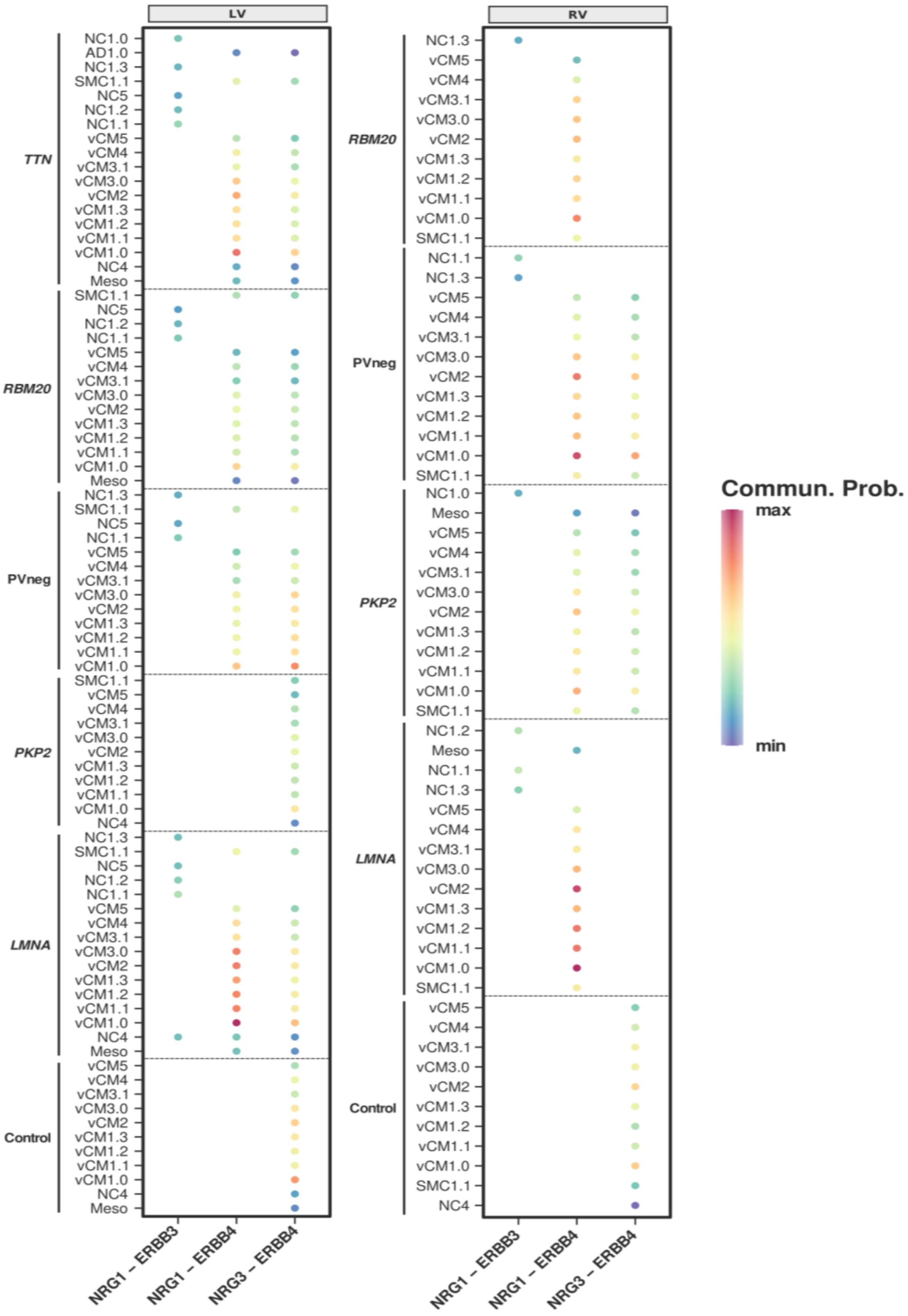

Figure S46: Communication probability dotplots of EC7.0 derived neuregulin (NRG) signaling.

Color of the dots represents the probability of communication for NRG receptor-ligands pairs (x-axis). The specific ligand, expressed by EC7.0, and receptor, expressed by the respective receiving cell-state is shown (y-axis).

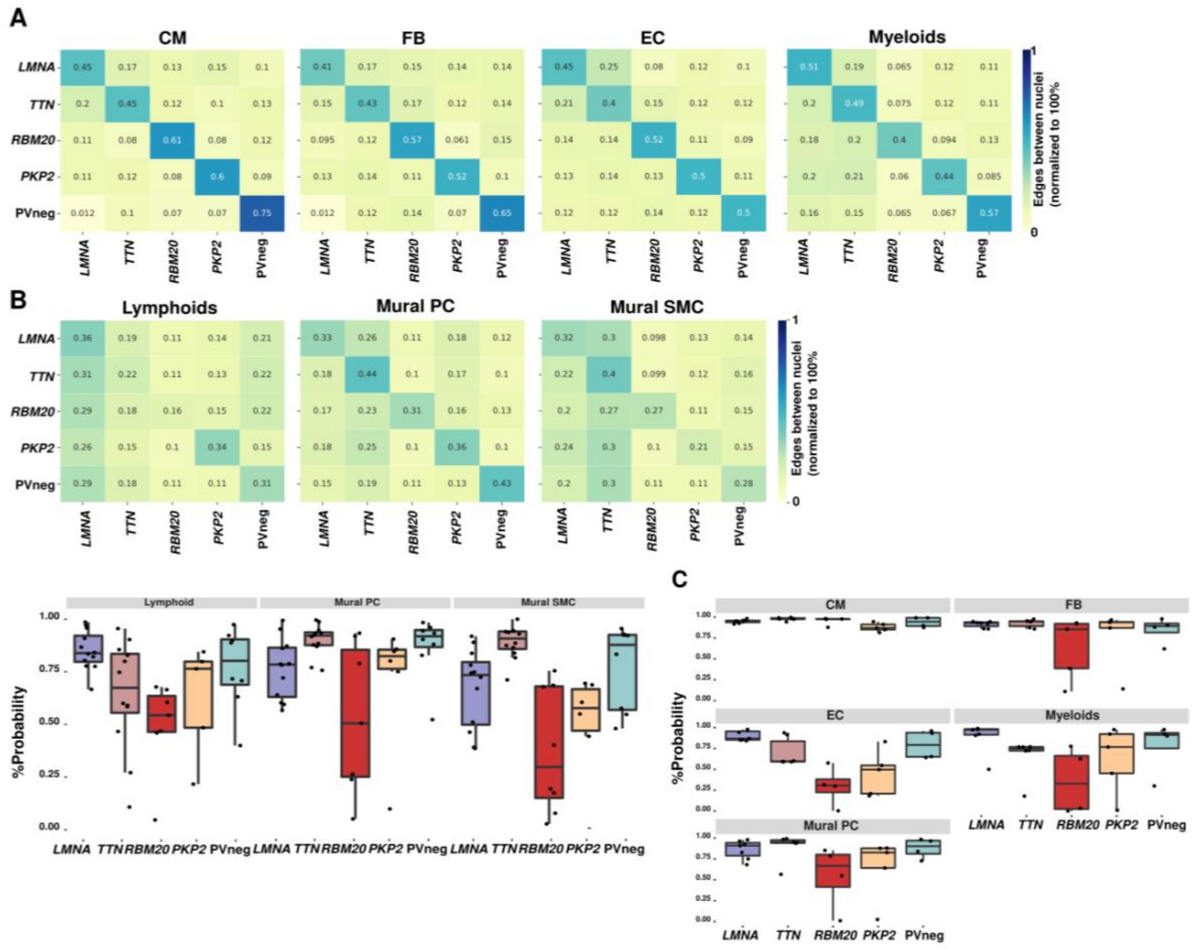

Figure S47: GAT predictions

(**A**) Heatmap of first-order neighbors between nuclei per genotype on the KNN graph for cell types used for aggregated GAT model construction (CM, FB, EC, myeloids). The numbers show the fraction (%) of edges from the KNN graph connecting nuclei from a patient with a particular genotype to nuclei from patients with another genotype. For example in CMs, among *LMNA* patients 45% of edges connected nuclei within the group, while 17% of the edges connected nuclei from *LMNA* and *TTN* patients. (**B**) (Top) Heatmap of first-order neighbors between nuclei per genotype on the KNN graph for cell types **not** used in the aggregated GAT Model (lymphoid, PC, SMC). (Bottom) Genotype prediction probability from graph attention networks (GAT) per cell-type in LV. (**C**) Genotype prediction probability from graph attention networks (GAT) per cell-type in RV. RV mural SMCs and lymphoids produced insufficient observations per patient to train genotype-prediction models.

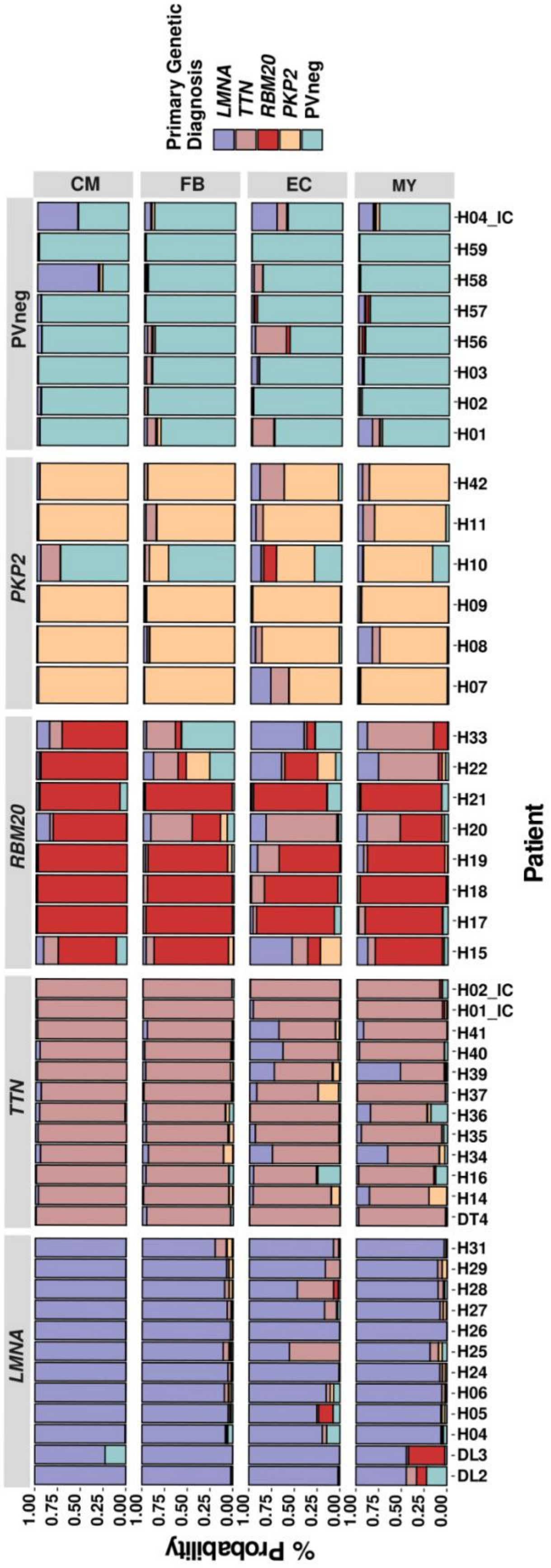

Primary Genetic Diagnosis
LMNA
TTN
RBM20
PKP2
PVneg
CM
FB
EC
MY
PVneg
PKP2
RBM20
TTN
LMNA
H04_IC
H59
H58
H57
H56
H03
H02
H01
H42
H11
H10
H09
H08
H07
H33
H22
H21
H20
H19
H18
H17
H15
H02_IC
H01_IC
H41
H40
H39
H37
H36
H35
H34
H16
H14
DT4
H31
H29
H28
H27
H26
H25
H24
H06
H05
H04
DL3
DL2
Patient
1.00
0.75
0.50
0.25
0.00
% Probability

Figure S48: Genotype prediction per patient sample

Stacked barplots represent the likelihood (% probability) of genotypes per LV cell type, for each patient before aggregation.

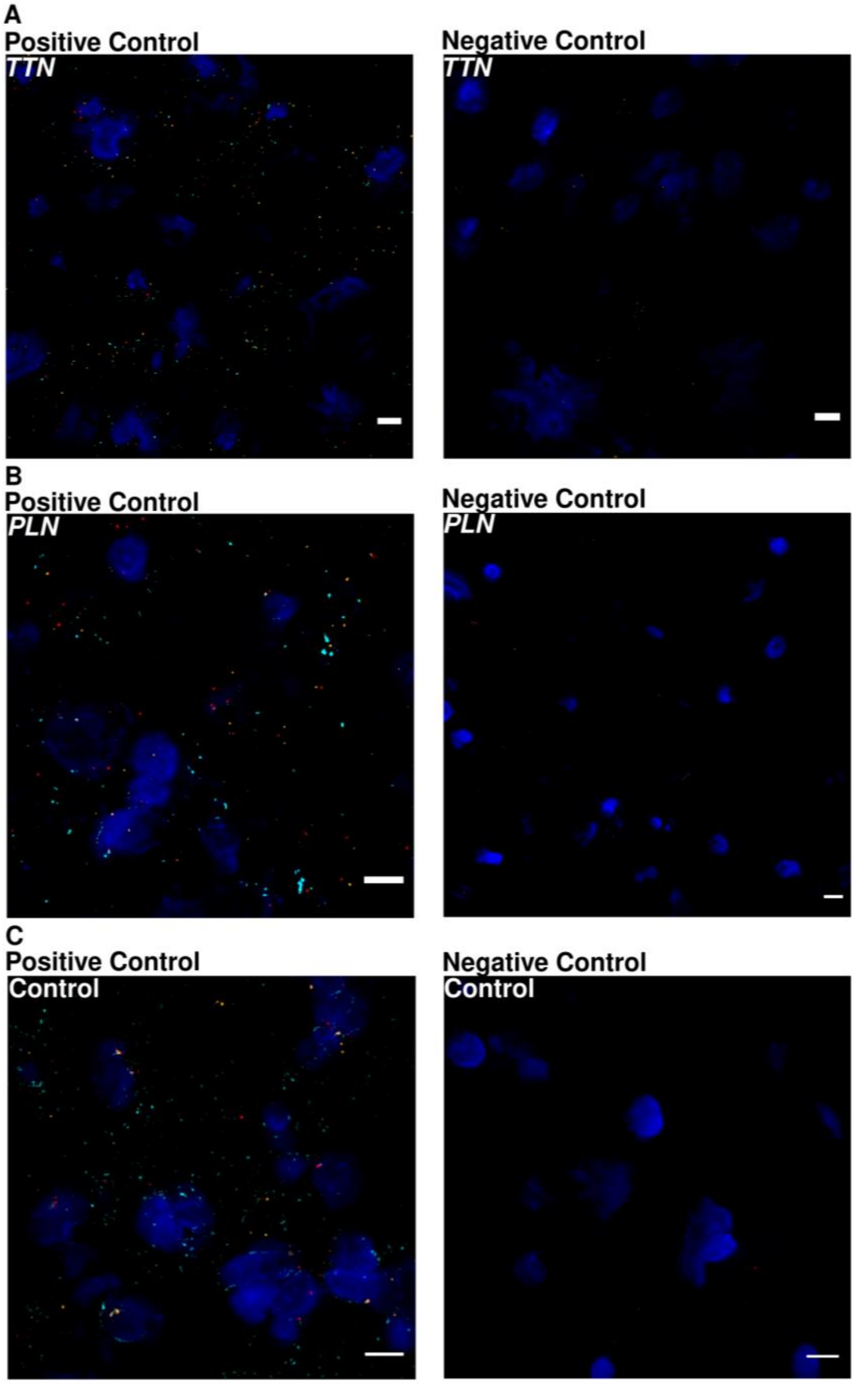

Figure S49*: In situ* hybridization of tissues used in Figs. 2C, 2J, S7D-E, and S13A with positive and negative control probes

(**A**) *TTN* LV (presented in Fig. 2J and S7D) hybridized with positive probe mix or negative (DapB) probe, provided by ACDBio. (**B**) *PLN* (phospholamban) LV (presented in Figs. 2C and S13A), studied as in (A). (**C**) Control RV (presented in Fig. S7E), studied as in (A).

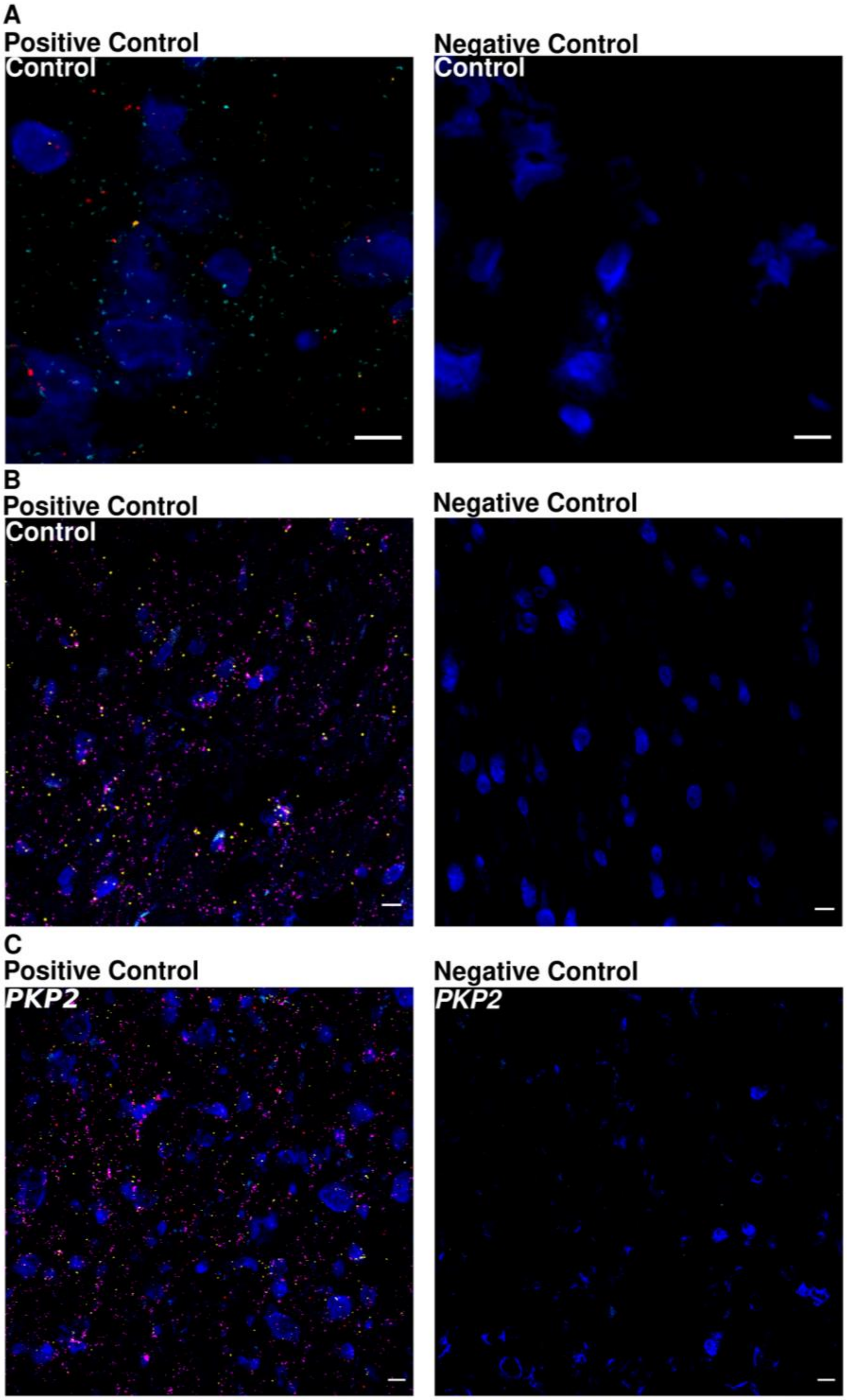

Figure S50: ***In situ*** **hybridization of tissues used in Figs. 2C-D and S6D with positive and negative control probes** (**A**) Control LV (presented in Figs. 2C) hybridized with positive probe mix or negative (DapB) probe, provided by ACDBio. (**B**) Control RVs (presented in Figs. 2D and S6D), studied as in (A). (**C**) PKP2 RVs (presented in Figs. 2D and S6D), studied as in (A).

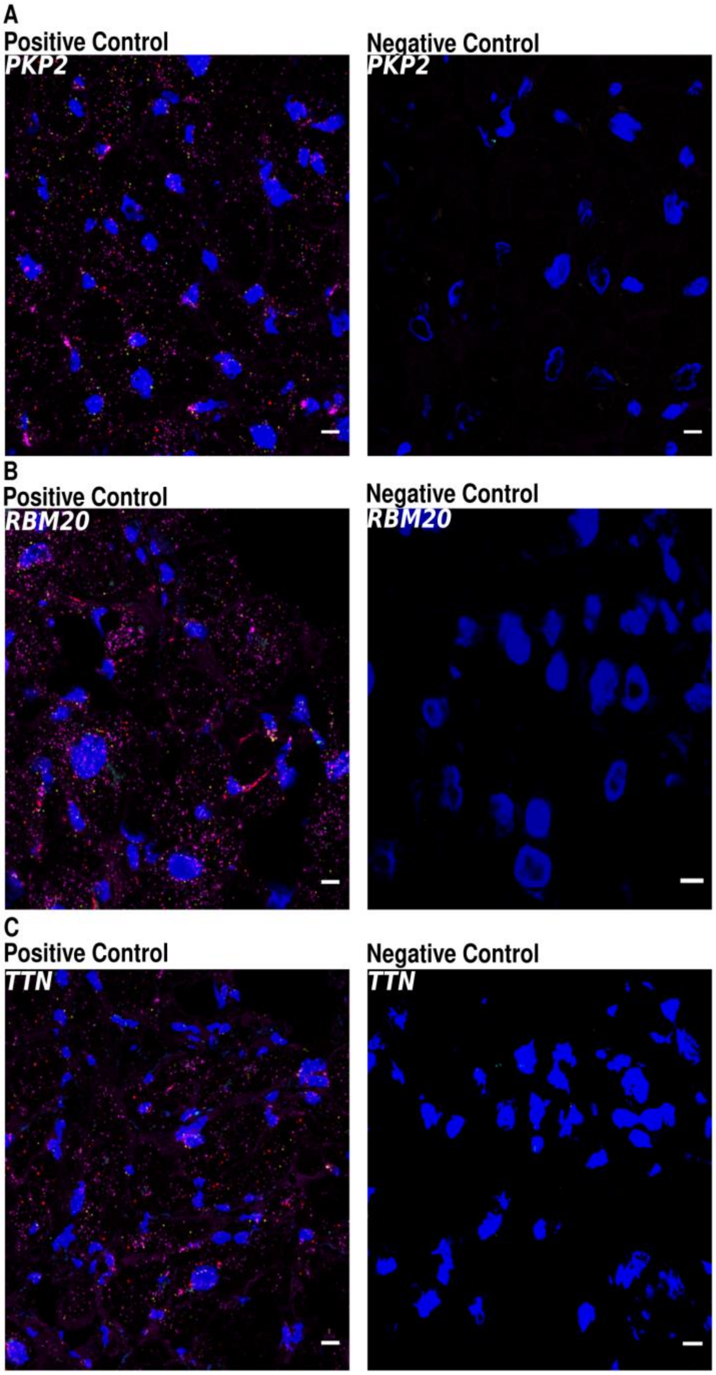

Figure S51: *In situ* hybridization of tissues used in Figs. 4C and S13B with positive and negative control probes (**A**) *PKP2* LV (presented in Fig. S13B) hybridized with positive probe mix or negative (DapB) probe, provided by ACDBio. (**B**) RBM20 LV (presented in Fig. S14C), studied as in (A). (**C**) *TTN* LV (presented in Fig. 4C), studied as in (A).

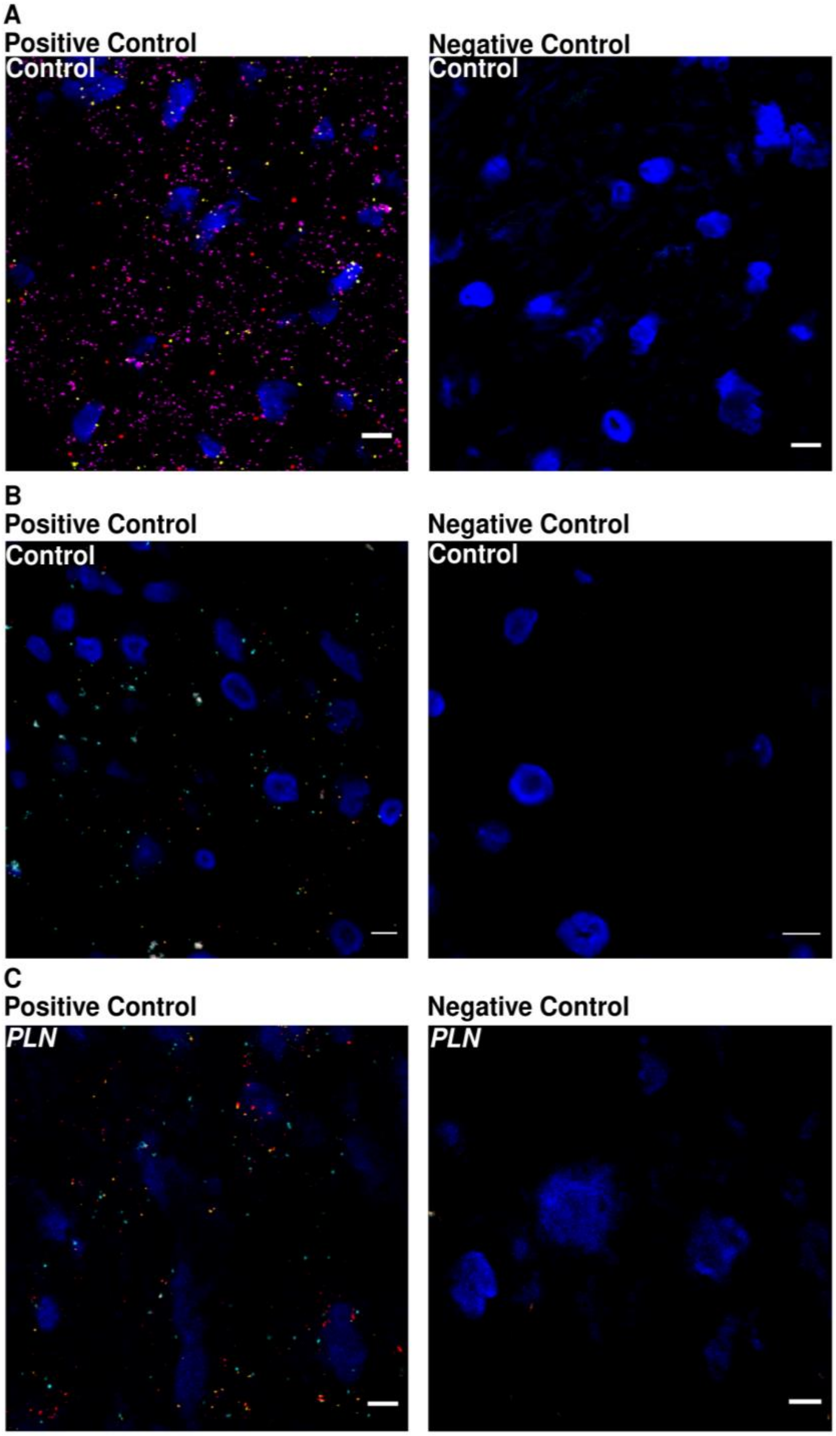

Figure S52: *In situ* hybridization of tissues used in Figs. 2J, 4C, and 5D with positive and negative control probes (**A**) Control LV (presented in Fig. 4C) hybridized with positive probe mix or negative (DapB) probe, provided by ACDBio. (**B**) Control LV (presented in Fig. 2J), studied as in (A). (**C**) *PLN* LV (presented in Fig. 5D), studied as in (A).

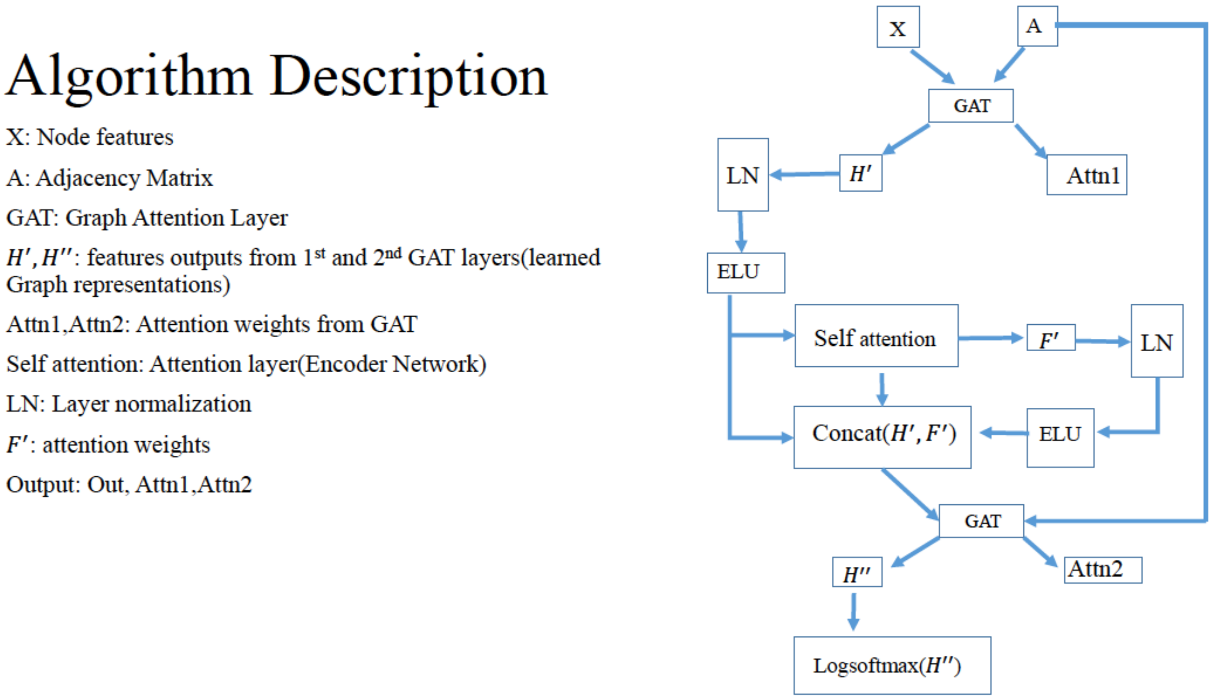

Figure S53: Schematic representation of Graph Attention Network (GAT) architecture.

	**Accuracy**	**F1 macro**
**Classical machine learning models**		
a. Random Forest	0.39	0.15
b. XGBOOST	0.34	0.14
c. KNN	0.26	0.13
**Neural network-based models**		
i) FFNN on count matrix	0.34	0.27
ii) SCANVI	0.4	0.21
iii) FFNN on graph embeddings	0.43	0.28
**Approach described in this manuscript**		
GAT	0.87	0.91

Table S71: Accuracy and F1 macro for alternative modeling strategies

Index to Tables S1 to S71

S1: Clinical_Metadata_Patient _Information.xlsx
S2: Sample_Information.xlsx
S3: Cell_Type_Abundance_LV.xlsx
S4: Cell_Type_Abundance_RV.xlsx
S5: Cell_States_Abundance_and_CLR_LV_RV.txt
S6: CM_Marker_Genes_Cell_States.xlsx
S7: LV_CM_Upregulated_Genes_Disease_Genotype_All_States.xlsx
S8: LV_CM_Upregulated_Genes_Controls_All_States.xlsx
S9: RV_CM_Upregulated_Genes_Disease_Genotypes_All_States.xlsx
S10: RV_CM_Upregulated_Genes_Controls_All_States.xlsx
S11: LV_CM_Cell_States DEGs (Folder)
S12: RV_CM_Cell_States DEGs (Folder)
S13: RNAscope_Quantification.xlsx
S14: Fibroblasts_Marker_Genes_Cell_States.xlsx
S15: Hydroxyproline_Table.xlsx
S16: LV_FB_Upregulated_Genes_Controls_All_States.xlsx
S17: LV_FB_Upregulated_Genes_Disease_Genotypes_All_States.xlsx
S18: RV_FB_Upregulated_Genes_Controls_All_States.xlsx
S19: RV_FB_Upregulated_Genes_Disease_Genotypes_All_States.xlsx
S20: LV_FB_Cell_States DEGs (Folder)
S21: RV_FB_Cell_States DEGs (Folder)
S22: MC_Marker_Genes_Cell_States.xlsx
S23: LV_MC_Upregulated_Genes_Disease_Genotypes_All_States.xlsx
S24: LV_MC_Upregulated_Genes_Controls_All_States.xlsx
S25: RV_MC_Upregulated_Genes_Disease_Genotypes_All_States.xlsx
S26: RV_MC_Upregulated_Genes_Controls_All_States.xlsx
S27: LV_MC_States DEGs (Folder)
S28: RV_MC_States DEGs (Folder)
S29: EC_Marker_Genes_Cell_States.xlsx
S30: LV_EC_Upregulated_Genes_Disease_Genotypes_All_States.xlsx
S31: LV_EC_Upregulated_Genes_Controls_All_States.xlsx
S32: RV_EC_Upregulated_Genes_Disease_Genotypes_All_States.xlsx
S33: RV_EC_Upregulated_Genes_Controls_All_States.xlsx
S34: LV_EC_Cell_States DEGs (Folder)
S35: RV_EC_Cell_States DEGs (Folder)
S36: Gene_Sets_EC.xlsx
S37: Myeloids_Marker_Genes_Cell_States.xlsx
S38: DEGs_LYVE1MP.csv
S39: DEGs_cDC.csv
S40: LV_Myeloids_Upregulated_Genes_Disease_Genotypes_All_States.xlsx
S41: LV_Myeloids_Upregulated_Genes_Controls_All_States.xlsx
S42: RV_Myeloids_Upregulated_Genes_Disease_Genotypes_All_States.xlsx
S43: RV_Myeloid_Upregulated_Genes_Controls_All_States.xlsx
S44: LV_Myeloids_Cell_States DEGs (Folder)

S45: RV_Myeloids_Cell_States DEGs (Folder)
S46: Lymphoids_Marker_Genes_Cell_States.xlsx
S47: LV_Lymphoids_Upregulated_Genes_Disease_Genotypes_All_States.xlsx
S48: LV_Lymphoids_Upregulated_Genes_Controls_All_States.xlsx
S49: RV_Lymphoids_Upregulated_Genes_Disease_Genotypes_All_States.xlsx
S50: RV_Lymphoids_Upregulated_Genes_Controls_All_States.xlsx
S51: LV_Lymphoid_Cell_States DEGs (Folder)
S52: RV_Lymphoid_Cell_States DEGs (Folder)
S53: NC_Marker_Genes_Cell_States.xlsx
S54: LV_NC_Upregulated_Genes_Disease_Genotypes_All_States.xlsx
S55: LV_NC_Upregulated_Genes_Controls_All_States.xlsx
S56: RV_NC_Upregulated_Genes_Disease_Genotypes_All_States.xlsx
S57: RV_NC_Upregulated_Genes_Controls_All_States.xlsx
S58: LV_NC_Cell_state DEGs (Folder)
S59: RV_NC_Cell_state DEGs (Folder)
S60: AD_Marker_Genes_Cell_States.xlsx
S61: LV_AD_Upregulated_Genes_Disease_Genotypes_All_States.xlsx
S62: LV_AD_Upregulated_Genes_Controls_All_States.xlsx
S63: RV_AD_Upregulated_Genes_Disease_Genotypes_All_States.xlsx
S64: RV_AD_Upregulated_Genes_Controls_All_States.xlsx
S65: LV_AD_Cell_state DEGs (Folder)
S66: RV_AD_Cell_state DEGs (Folder)
S67: GWAS.xlsx
S68: Cellchat_heatmap_table_supplement.csv
S69: Aggregation_All_Patients.xlsx
S70: Aggregated_Scores.xlsx
S71: Accuracy and F1 macro for alternative modeling strategies
